# Cethrene’s Catch-22: Is Trading Magnetism for Bistability Inevitable?

**DOI:** 10.1021/acs.orglett.5c04279

**Published:** 2025-12-09

**Authors:** Pauline Pfister, Daniel Čavlović, Tamara Trajkovic, Olivier Blacque, Michal Juríček

**Affiliations:** Department of Chemistry, 27217 University of Zurich, Winterthurerstrasse 190, 8057 Zurich, Switzerland

## Abstract

Cethrenes represent a promising platform for realizing all-organic magnetic photoswitches—provided that three requirements are met. The open form needs a thermally accessible triplet state to unlock magnetic properties, its energy relative to the closed form should lie within an accessible range, and the energy barrier between the two forms must be sufficiently high to achieve bistability. However, fulfilling the first and second criteria appear to compromise the third. Can this Catch-22 be solved? [*Oxford English Dictionary* defines a Catch-22 as a difficult situation or problem which cannot be resolved because the conditions necessary for its resolution are paradoxical or conflicting, with allusion to Joseph Heller’s 1961 novel of the same name.]

## Graphical abstract

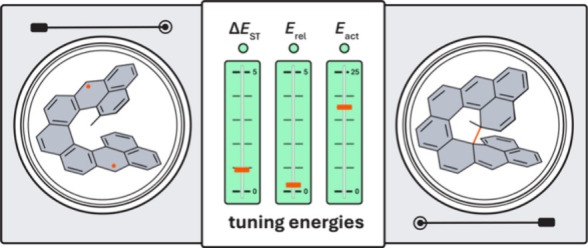

The realization of an all-organic system capable of switching between magnetic and nonmagnetic forms at ambient temperature would constitute a key milestone in a field long dominated by metal-based materials.^3,4^ Organic radicals,^5^ which benefit from inherently weak spin–orbit couplings and low hyperfine interactions, have already demonstrated promising magnetic behavior.^6,7^ However, in contrast to metal-based systems, where spin states can be reversibly modulated by tuning the metal’s coordination environment,^8,9^ realizing magnetic bistability in organic molecules presents a fundamentally different challenge. It typically^10^ requires covalent bond cleavage to generate radical centers.^11^ Since electrons naturally favor bonding, the open-shell form is often short-lived, rapidly reverting to the closed-shell form.^12^ This raises a central question: what design principles permit the construction of a robust, magnetically bistable organic switch? Addressing this challenge requires fulfilling three criteria:

1. **Paramagnetic state.** One form must be paramagnetic and the other diamagnetic. Typically, this means the paramagnetic form either has a triplet ground state or a thermally accessible triplet excited state at the operating temperature.
2. **Energetic proximity.** The two forms of the switch must be energetically accessible. Since bond cleavage is involved, the targeted bond must be sufficiently weakened (destabilized), while the resulting diradical form must be stabilized to prevent rapid recombination. One effective strategy for weakening covalent bonds is the introduction of strain,^13^ while stabilization of the diradical form can be achieved through various effects, including delocalization of unpaired electrons^14,15^ and aromatic stabilization.^16–18^

3. **Sufficient activation barrier.** The activation energy for interconversion must be high enough to ensure bistability at room temperature.

To realize controlled switching, the system must respond to a suitable external stimulus. Out of several alternatives, including mechanical force^19^ or pH changes,^20^ light represents a particularly versatile trigger for bond breaking. Although optical methods face certain resolution limits,^21^ they offer distinct advantages: light is minimally invasive, its energy can be tuned, and it enables spatial control in three dimensions.^22^

The seminal examples of organic magnetic switches utilizing these design principles rely on the photochemical cleavage of a π-bond accompanied by a conformational change, while preserving the overall atomic connectivity.^23^ This mechanism operates in overcrowded ene systems, where cleavage of the weakened π-bonds is driven by strain release, inducing transition from a folded, closed-shell structure to a twisted, diradical conformer. Depending on the specific system, relatively high activation barriers can be achieved,^24^ resulting in significantly slowed thermal back-isomerization and diradical half-lives of up to 4 h at 55 °C.^25^ These systems exhibit a clear transition^26,27^ between diamagnetic (EPR-silent) and paramagnetic (EPR-active) states, but they require two different triggers—light for the forward reaction and heat for the reverse.

Our group has developed a platform^28^ that provides the foundation for bidirectional, light-driven switching between distinct magnetic states (Figure 1). The key feature of this design is that a σ-bond—rather than a π-bond—undergoes photochemical homolytic cleavage, thereby inducing a change in the overall atomic connectivity. The two formally unpaired electrons generated in this process can recombine through a conjugated framework; however, this pairing entails an energetic penalty—the loss of one Clar sextet—which reduces the system’s overall aromatic stabilization energy. The resulting species is a diradicaloid system with a singlet ground state and a thermally accessible triplet excited state,^29,30^ the magnetically active form of the switch. This design introduces a novel approach to magnetic bistability, where switching is controlled by a photochemical 6π electrocyclic ring-opening/closure.

**Figure 1:**
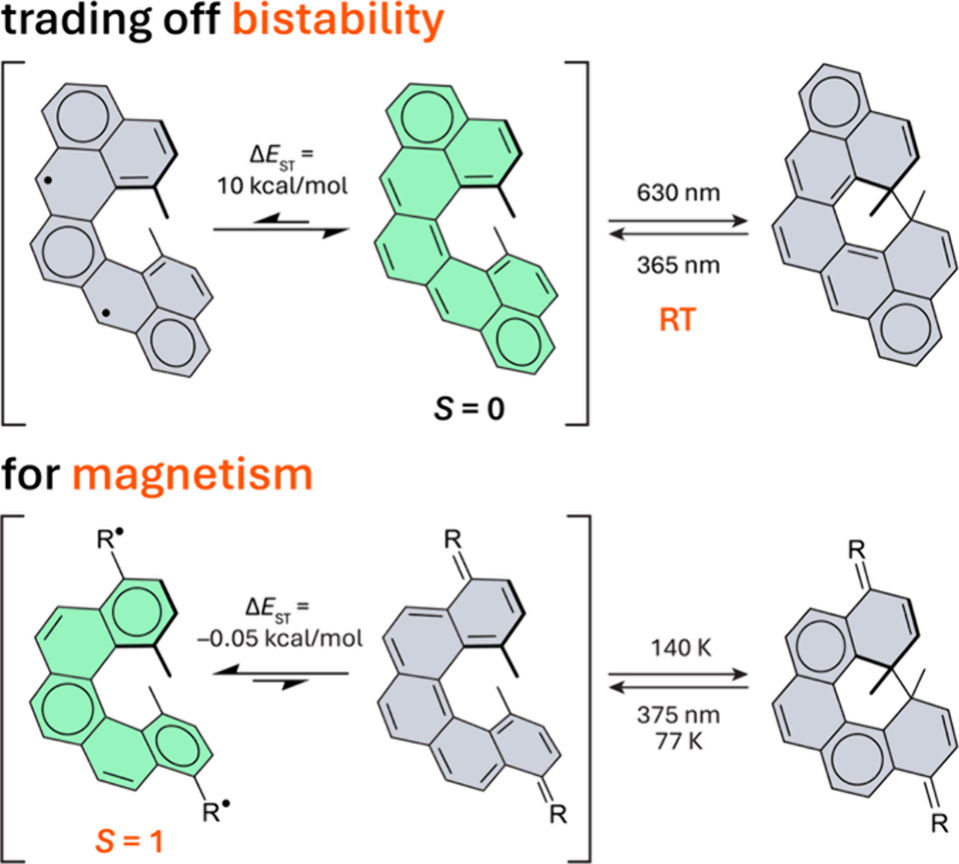
Trading off bistability for magnetism. Illustrated using dimethylcethrene^31^ (top) and Dumele’s [5]­helicene^34,35^ (bottom) as examples of organic photoswitches. Structures corresponding to the ground state are highlighted in green. R = O, C­(CN)_2_, or indanedione.

In our prototype system, dimethylcethrene (Figure 1, top), bistability was demonstrated under ambient conditions. The open form exhibited a lifetime of several hours at room temperature, underscoring the system’s stability.^31^ In contrast, the diradical conformer in earlier magnetic switches based on σ-bond cleavage was detectable only on the microsecond time scale and rarely persisted more than a few seconds.^32,33^ However, in dimethylcethrene the triplet state remained too high in energy to be detected by EPR spectroscopy, preventing the molecule from functioning as a magnetic switch.

Dumele and co-workers identified a [5]­helicene switch based on σ-bond cleavage, in which the triplet-state energy was lowered to the point that it became the ground state, as electron pairing in the open form would otherwise disrupt aromaticity across the entire [5]­helicene core (Figure 1, bottom).^34^ Incorporation of π-acceptor units further reduced the HOMO–LUMO gap, enabling switching with visible light.^35^ While this additional stabilization allowed magnetic switching between EPR-active and EPR-silent states, the enhanced radical character substantially lowered the σ-bond forming barrier, accelerating thermal back-reaction and restricting switch operation to cryogenic temperatures (77 K). Even metal-based spin-crossover systems are often limited to operation below 80 K, as spin relaxation becomes rapid at higher temperatures.^36^

Together, these studies suggest that dimethylcethrene could serve as a magnetic photoswitch exhibiting bistability at room temperature with fully light-driven, bidirectional operation, if the singlet–triplet gap can be further decreased. With the goal of lowering the energy of the excited triplet state, we extended the π-backbone from [5]- to [7]­helicene, yielding dimethylnonacethrene (**DMNC**, Figure 2) with a narrow singlet–triplet gap of ∼1 kcal/mol and increased steric congestion in the fjord region.^37^ For the first time in the cethrene series, the open form was more stable than the closed form, allowing the magnetically active open isomer to be isolated under ambient conditions. However, the closed form *c*-**DMNC** could not be generated upon irradiation, even at 77 K. For comparison, in nonacethrene without methyl substituents (**NC**, R = H, Figure 2), neither the open nor the closed form could be observed because the system undergoes a complex reaction cascade, with these species thought to be intermediates.^38^

**Figure 2:**
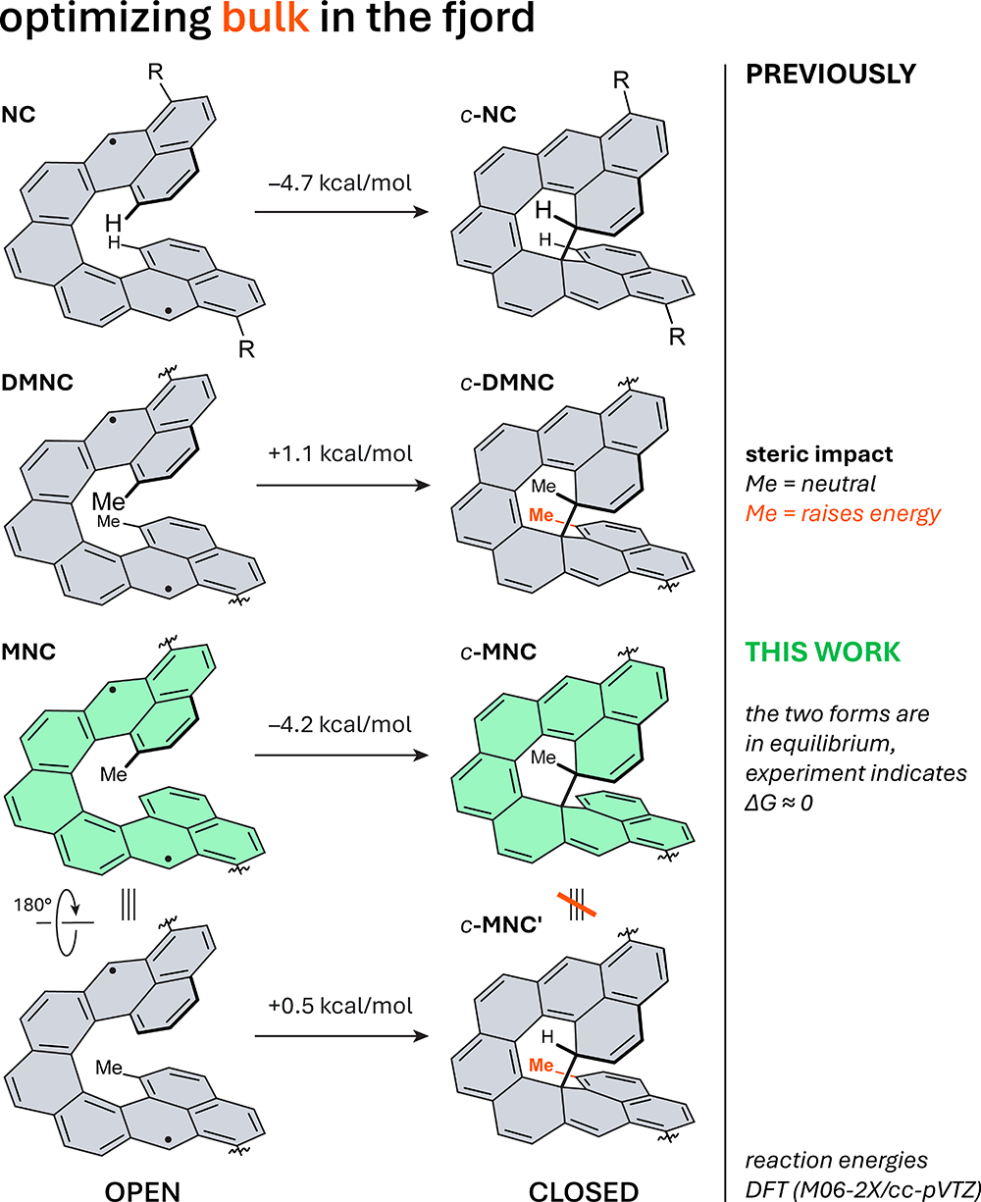
Optimizing steric bulk in the fjord region. Reaction energies (shown above arrows; see SI for details) for the transformation of **NC**, **DMNC**, and **MNC** to their closed forms reveal two types of Me groups, neutral (black) and destabilizing (orange), with respect to the energy of the closed form. *c*-**MNC** is the only closed form observed experimentally. R = 3,5-di-*tert*-butylphenyl.

It was hypothesized that the narrow singlet–triplet gap in **DMNC** either lowers the activation barrier so that the back ring-closing reaction becomes too fast even at cryogenic temperatures (77 K), or, less likely, that it is too high to allow photochemical ring closure due to steric hindrance in the fjord region. In either case, finding the optimal steric congestion appeared crucial. Increasing the bulk of the fjord substituents would likely further destabilize the closed form, whereas their removal reintroduces undesired cascade reactivity. To address this issue, we introduced a single methyl group in the fjord region. The resulting methylnonacethrene (**MNC**, Figure 2) can undergo two types of ring closure, yielding *c*-**MNC** or *c*-**MNC′**.

DFT calculations (Tables S2 and S8) comparing the open and closed forms of **NC**, **MNC**, and **DMNC** reveal that the two methyl groups—nonequivalent in the closed structure—contribute differently to relative molecular stability. Closure of **NC** is comparably exoergic to closure of **MNC** to *c*-**MNC** (−4 to −5 kcal/mol), while closure of **DMNC** is comparably endoergic to closure of **MNC** to *c*-**MNC′** (∼+1 kcal/mol). This suggests that destabilization arises primarily from the methyl group pointing toward the backbone (in *c*-**MNC′**) rather than from the one incorporated into the new ring and pointing away (in *c*-**MNC**). Although different functionals produce different results regarding relative stabilities, the trend of *c*-**MNC′** being around +5 kcal/mol higher in energy than *c*-**MNC** is consistent across all of them (Table S3). Fluctuations in the calculation results can be traced back to the fact that DFT, including broken-symmetry approaches, is not always a reliable method for calculating systems with a high radical character.^23,39^

This outcome is advantageous. Unlike *c*-**MNC′**, whose tertiary sp^3^ carbon atom is susceptible to hydrogen abstraction by oxidants, *c*-**MNC** with a quaternary sp^3^ carbon atom is not prone to cascade reactivity. This feature motivated the synthesis of the **MNC** derivative presented here. **MNC** provides a platform to raise the reaction barrier via steric modification of the fjord substituent, while keeping the open and closed forms similar in energy, a key to realizing a magnetic switch.

The synthesis of **MNC** followed established routes for **NC** ^38^ and **DMNC** ^37^ (Scheme 1). To make the two fjord positions nonequivalent (H vs Me), 3,6-dibromophenanthrene was selectively monoformylated to give monocarbaldehyde **1** (76%), which was used to install the side chain without the Me group via a Wittig reaction. A subsequent Heck coupling introduced the methyl-substituted chain, affording intermediate **5** in 53% yield over the two steps. Two-fold photocyclodehydrogenation furnished **6**, bearing the [7]­helicene backbone, in 30% yield. Ester hydrolysis, halogenation, and Friedel–Crafts cyclization provided diketone **7** in 56% yield over three steps. Subsequent reduction and dehydration gave the dihydro-precursor **8** in 67% yield, representing the most stable isomer of the product that would be formed directly upon dehydration. This is a typical behavior in systems of this type.^40^ Since oxidation of **8** with *p*-chloranil afforded a poorly soluble product that could not be characterized, two 3,5-di-*tert*-butylphenyl groups (R) improving solubility were introduced via Grignard addition to **7** and, after dehydration, yielded the substituted dihydro-precursor **9** in 58% yield, again as the most stable isomer.

**Scheme 1:**
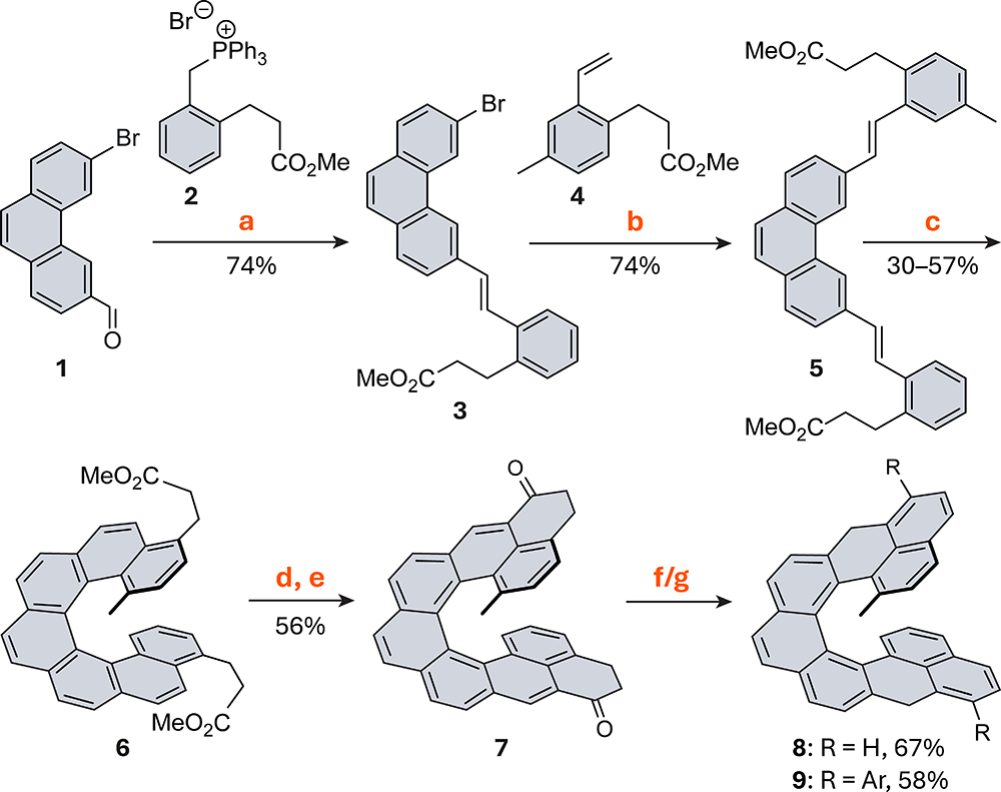
Synthesis of MNC Dihydro-Precursors^a^

a Reaction conditions: (a) NaOMe, THF, r.t., 12 h; (b) Pd­(OAc)_2_, tetrabutylammonium bromide, PPh_3_, K_2_CO_3_, DMF, 80 °C, 16 h; (c) *hv*, propylene oxide, toluene, 1.5 h; (d) LiOH, THF/H_2_O (3/1), 80 °C, 4 h; (e) TiCl_4_, CH_2_Cl_2_, −50 to −30 °C, 5.5 h; (f) **8**: NaBH_4_, DCM/MeOH (1/1), r.t., 30 min, then *p*-toluenesulfonic acid, toluene, 95 °C, 5 min; (g) **9**: ArMgBr, CeCl_3_, LiCl (0.5 M in THF), –78 °C, 16 h. Ar = 3,5-di-*tert*-butylphenyl.

To generate **MNC**, oxidation of **9** was performed with varying equivalents of *p*-chloranil in benzene-*d*_6_ and toluene-*d*_8_ directly in an NMR tube. The reaction was monitored by NMR spectroscopy. Within 30 min, the solution changed from yellow to deep red, accompanied by complete consumption of the starting material (Figure 3, top and Figure S1). NMR spectra showed signals of a single species, identified by 2D NMR spectroscopy (see the Supporting Information) as the closed form of **MNC** (*c*-**MNC**), representing the first successful characterization of a nonoxidized, closed nonacethrene derivative. Even after 14 days of exposure to excess *p*-chloranil, no significant spectral changes were observed (Figure S2), indicating that no further cascade reactions occur after the 6π electrocyclic ring closure.

**Figure 3:**
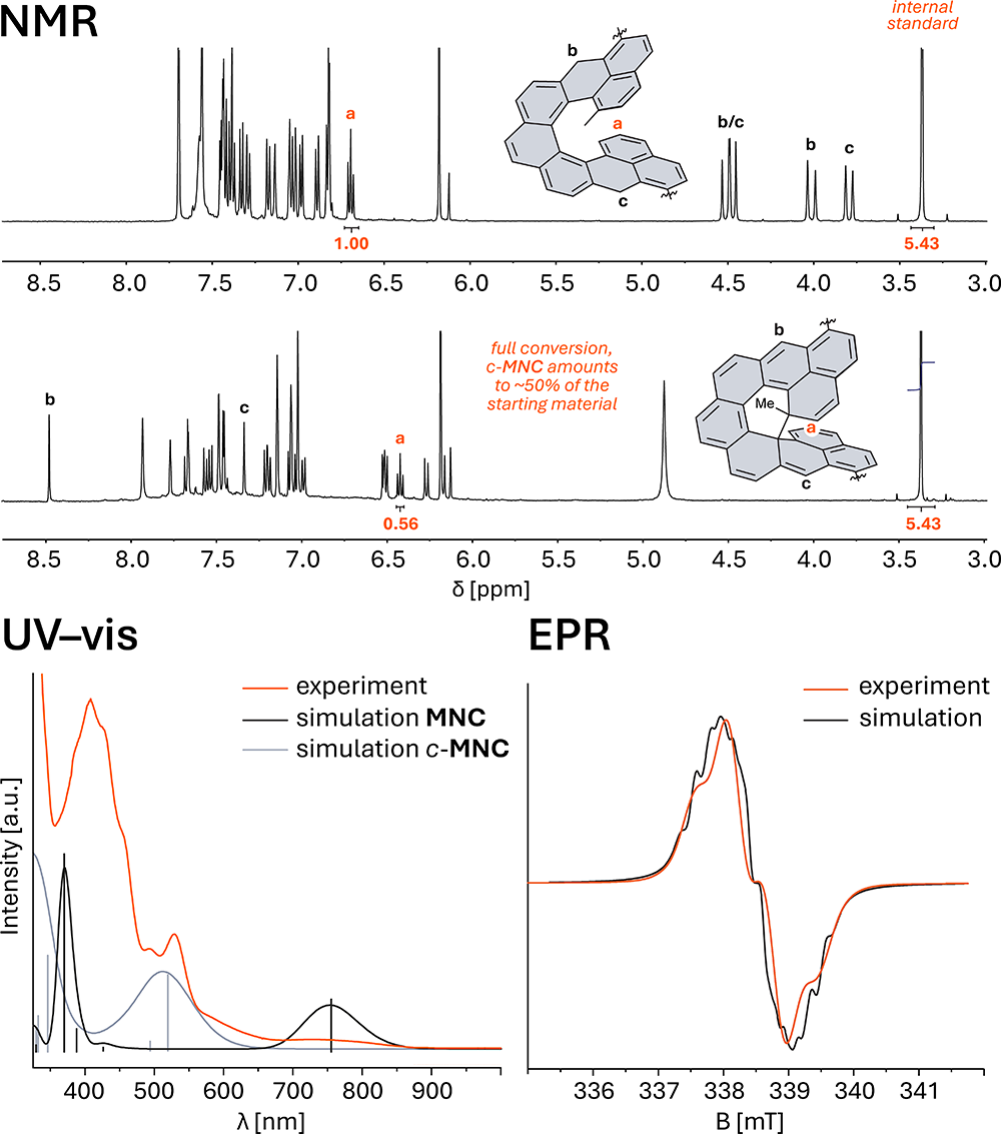
Analysis of reaction species during oxidation of **9**. NMR: ^1^H NMR spectrum of **9** before (top) and 30 min after the addition of 1.1 equiv of *p*-chloranil (bottom) in toluene-*d*_8_ at room temperature. 1,3,5-Trimethoxybenzene (signals at 3.37 and 6.29 ppm) was used as an internal standard. UV–vis and EPR: Comparison of the simulated (black and gray) and experimental (orange) spectra of **MNC**/*c*-**MNC** mixture (*c* = 0.31 mM (UV–vis) and *c* = 3.7 mM (EPR) in toluene). Parameters are shown in Table S11, and Figures S22 and S23.

Using 1,3,5-trimethoxybenzene as an internal standard revealed full consumption of the staring material but only ∼50% was converted into *c*-**MNC**, with no detectable NMR-active side products (Figure 3, top and Figure S3). Therefore, the progress was monitored by EPR spectroscopy. After ∼30 min, a quintet signal appeared, consistent with a monoradical formed by hydrogen abstraction from **9**, as supported by simulation using DFT-calculated hyperfine coupling constants (Figures S20 and S21). Upon standing for several hours, this signal broadened and evolved into a spectrum matching that simulated for the triplet state of diradicaloid **MNC**, indicating reaction completion (Figure 3, bottom right and Table S2; Figure S30 shows the triplet-state spin-density plots).

The crude reaction mixture was purified by filtration over a short plug of silica to remove residual *p*-chloranil and its reduced form, affording a red solid in 65% isolated yield. The UV–vis spectrum of the isolated material displayed a broad band at 746 nm, characteristic^41^ of a narrow HOMO–LUMO gap of **MNC**, and an additional band at 531 nm, consistent with the computed spectrum of *c*-**MNC** (TD-DFT/M06-2X-D3/cc-pVTZ, Figure 3, bottom left). These results indicate that **MNC** and *c*-**MNC** coexist in the mixture. As noted above, the relative energies of the open and closed forms vary slightly from negative to positive depending on the functional used (Tables S2 and S3), reflecting their energetic proximity and suggesting a possible equilibrium.

To investigate the thermal equilibrium, temperature-dependent NMR and UV–vis experiments were performed. As shown in Figure 4, the ^1^H NMR spectrum of the mixture in toluene-*d*_8_ at room temperature exhibits sharp signals for diamagnetic *c*-**MNC**, with no detectable signals for **MNC**. This behavior is consistent with related systems. For comparison, the Chichibabin hydrocarbon reported by von Delius et al., which has a singlet–triplet gap of ∼2 kcal/mol, shows no ^1^H NMR signals at room temperature,^42^ while only broadened signals^43^ are observed for the octazethrene derivative reported by Wu et al. with a gap of ∼4 kcal/mol.^44^ Given the narrow ∼1 kcal/mol singlet–triplet gap estimated for **MNC**, the absence of observable signals at room temperature is likewise expected. Upon heating to 363 K, the sharp signals of *c*-**MNC** exhibit pronounced broadening, indicating a transition from slow to intermediate exchange on the NMR time scale and supporting an equilibrium between the paramagnetic and diamagnetic species. In contrast, upon cooling to 193 K, the resonances of the diamagnetic species remain well resolved, consistent with slow exchange, while the slight broadening likely reflects reduced solubility. Freezing of rotation of the solubilizing aryl groups is also observed.

**Figure 4:**
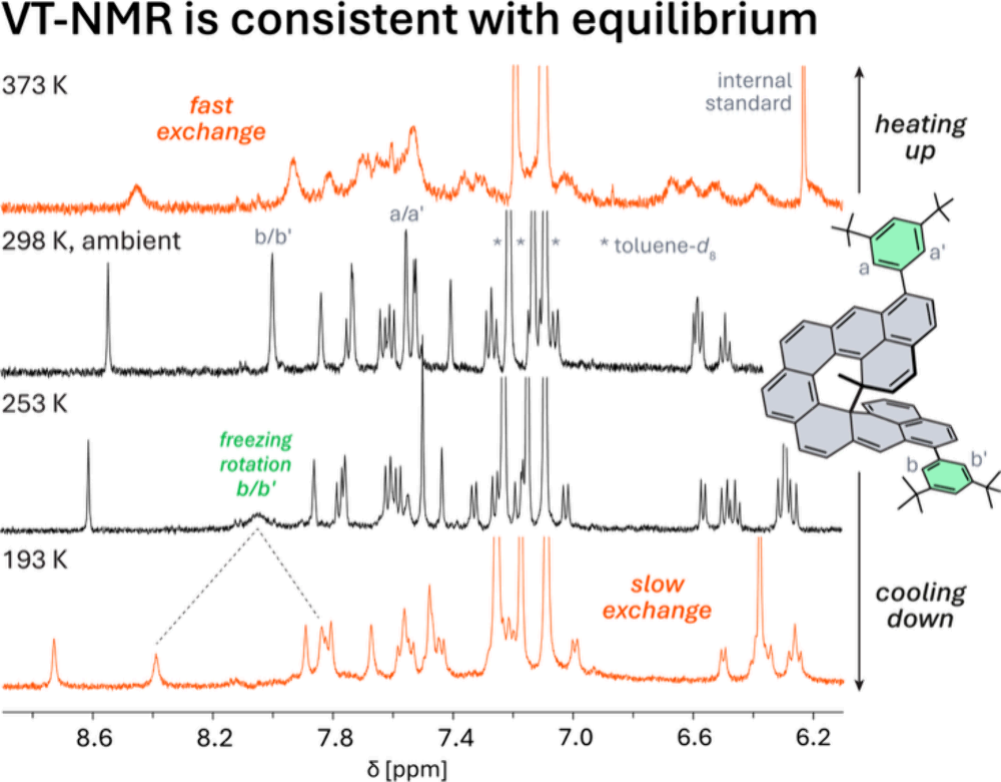
Probing the equilibrium between **MNC** and *c*-**MNC**. Variable-temperature (VT) ^1^H NMR spectra of **9** with an internal standard (1,3,5-trimethoxybenzene) in toluene-*d*_8_. Residual-solvent and internal-standard signals are partially cut off for clarity.

Variable-temperature UV–vis spectra showed no significant changes in band intensities, consistent with an energetically balanced equilibrium, though a slight decrease in overall absorbance at higher temperatures indicates minor decomposition (Figures S24 and S25).

So far, **MNC** meets two of the three criteria discussed in the introduction: it has a small singlet–triplet gap (0.9 kcal/mol), rendering it EPR-active at room temperature, and the open and closed forms are close enough in energy that both can be observed simultaneously. Additionally, the introduction of a methyl group prevents the reaction cascade seen with pristine **NC**. Nevertheless, irradiating a solution of **MNC** in toluene under inert conditions with various wavelengths (505, 405, and 365 nm) and monitoring the process by UV–vis spectroscopy only led to partial decomposition (Figures S26–S28).

One possible explanation is that the activation barrier is insufficient to allow the equilibrium to be shifted by irradiation. We therefore computed the barrier from **MNC** to *c*-**MNC** using DFT, obtaining values of 15.5–18.1 kcal/mol depending on the functional used (Tables S4 and S5). For comparison, the calculated barrier for closure of parent **NC** is slightly smaller (14.3–17.9 kcal/mol, likely within computational error; Tables S6 and S7), while the experimentally measured barrier for dimethylcethrene is 20 kcal/mol,^31^ and Feringa’s twisted-to-folded switch has a barrier of 25 kcal/mol.^25^ Although the barrier in **MNC** is lower than in these two examples, it remains within a range where the two forms could, in principle, be observed separately, provided that photoswitching selectively triggers electrocyclic ring opening and closing. The sharp ^1^H NMR signals observed at room temperature further support a relatively high barrier; if it were very small, signal broadening, as seen at higher temperatures, would be expected.

So, what is the *catch*? Previous studies have suggested that a high radical character lowers the reaction barrier.^37,45,46^ In the case of **MNC**, however, steric effects in the fjord region appear to raise the barrier sufficiently to make switching possible. In other words, the original Catch-22 seems to have been resolved—yet switching still does not occur. Possible reasons for the lack of switching include alternative decay pathways following excitation, such as an excited-state minimum in the funnel region of the potential-energy surface that does not efficiently promote switching in one direction,^47^ competing decay pathways^48^ that proceed faster than the switching process and lead to unwanted side reactions, or tunneling processes that facilitate rapid back-reactions. The simultaneous observation of **MNC** and *c*-**MNC** was crucial for these insights. Their coexistence allowed us to directly assess the role of the reaction barrier. Attempts to shift the equilibrium thermally or photochemically were unsuccessful, demonstrating that simply increasing the barrier is insufficient to achieve switching in this case. These factors underscore that a full understanding of this and related systems is necessary before efficient molecular magnetic photoswitches can be rationally designed. These endeavors will be continued in our laboratory.

## Supplementary Material



## Data Availability

The data underlying this study are available in the published article, in its Supporting Information, and openly available in the public repository Zenodo at https://zenodo.org/record/17350538. An earlier version of this work is available as a preprint on *ChemRxiv*.^49^

## Notes

Supporting figures, synthetic protocols, and analytical characterization of the prepared compounds, copies of NMR and HRMS spectra, summary of EPR, UV–vis, XRD, and DFT results, including Cartesian coordinates of optimized geometries (PDF)
