# Supplementary material for: Cethrene’s Catch-22: Is Trading Magnetism for Bistability Inevitable?
Source: Org Lett. 2025 Dec 9;27(50):13798–803. doi: 10.1021/acs.orglett.5c04279 (PMC12723680; doi:10.1021/acs.orglett.5c04279)
Supplement: Supplementary file 1 [file ol5c04279_si_001.pdf]

Supporting Information for

Cethrene's Catch-22: Is Trading Magnetism for Bistability Inevitable?

Pauline Pfister, Daniel Čavlović, Tamara Trajkovic, Olivier Blacque, Michal Juríček*

Department of Chemistry, University of Zurich, Winterthurerstrasse 190, 8057 Zurich, Switzerland

Table of Contents

1. Supporting Figures S2
2. Synthesis S5
3. Assignment of the NMR Resonances S16
4. Copies of the NMR Spectra S19
5. Copies of the HRMS Spectra S36
6. EPR Spectroscopy S45
7. UV–Vis–NIR Spectroscopy and Irradiation Studies S48
8. X-Ray Crystallography S51
9. DFT Calculations S52
10. Cartesian Coordinates S70
11. References S96

The raw data are openly available in the public repository Zenodo at https://zenodo.org/record/17350538 (DOI: 10.5281/zenodo.17350538).

1. Supporting Figures

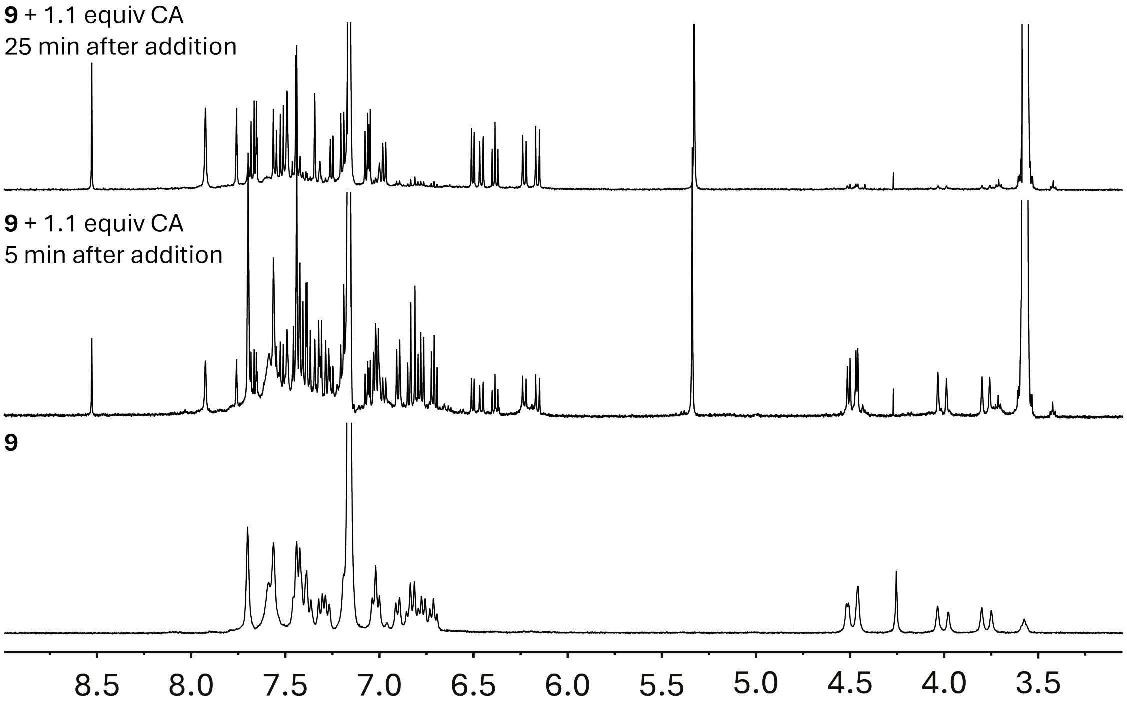

Figure S1. ^{1}H NMR spectra of **9** in benzene-d_6 before (bottom) and after addition of 1.1 equivalents of CA (middle, 5 min and top, 25 min) (*x*-axis in δ[ppm]). CA = *p*-chloranil.

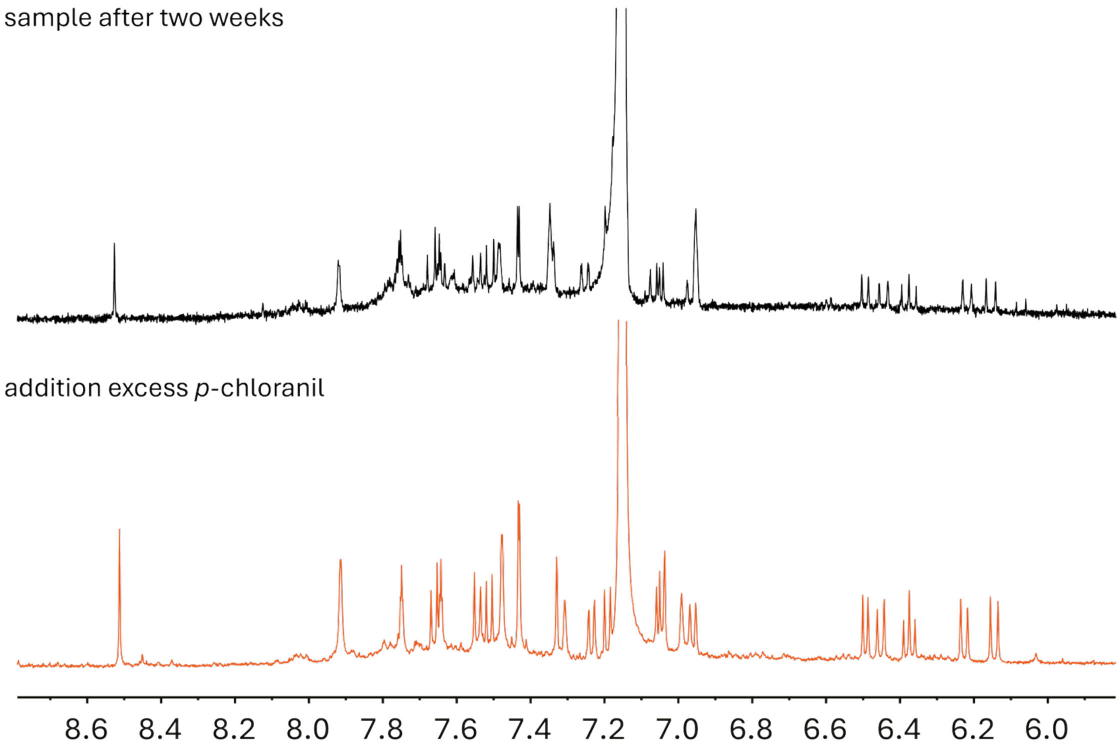

Figure S2. ^{1}H NMR spectra of **9** in benzene-d_6 after addition of excess (~100 equivalents) of CA (bottom) and the same sample measured two weeks after the addition (top) (*x*-axis in δ[ppm]). CA = *p*-chloranil.

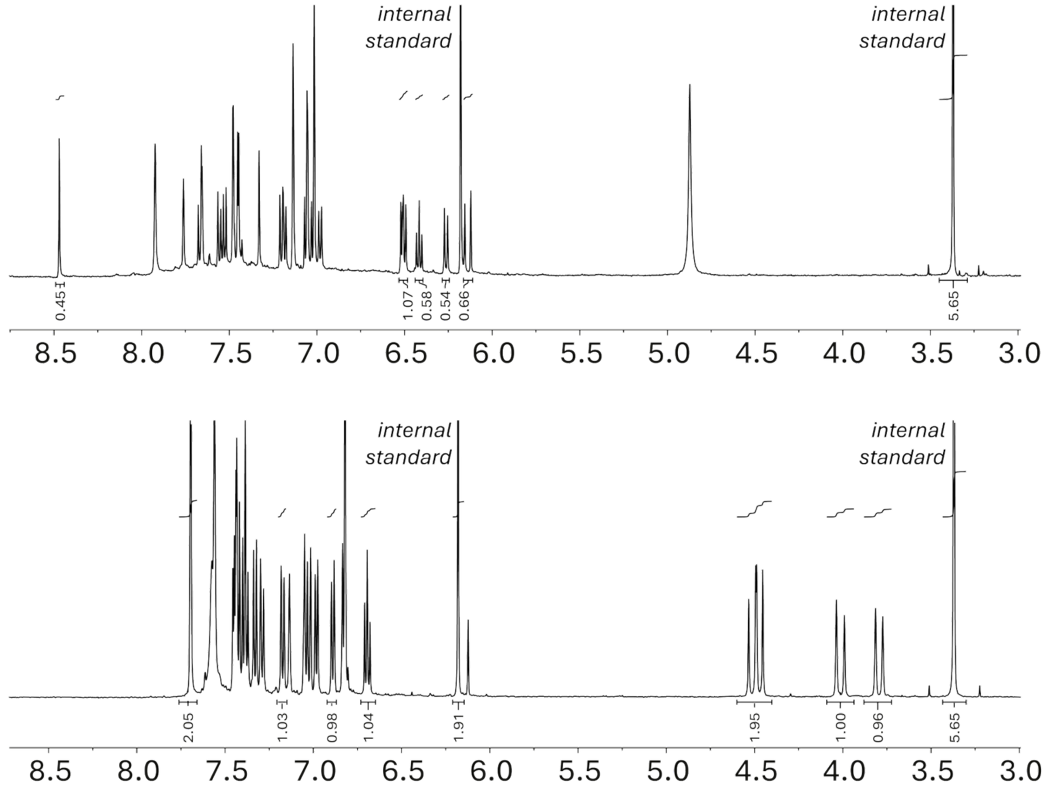

Figure S3. Supplementary figure to Figure 3. ^{1}H NMR spectra of **9** before (top) and after addition (bottom) of 1.1 equivalents of *p*-chloranil in benzene-d_6, indicating that *c*-**MNC** amounts to 50% of the starting material. 1,3,5-Trimethoxybenzene was used as an internal standard (*x*-axis in δ[ppm]).

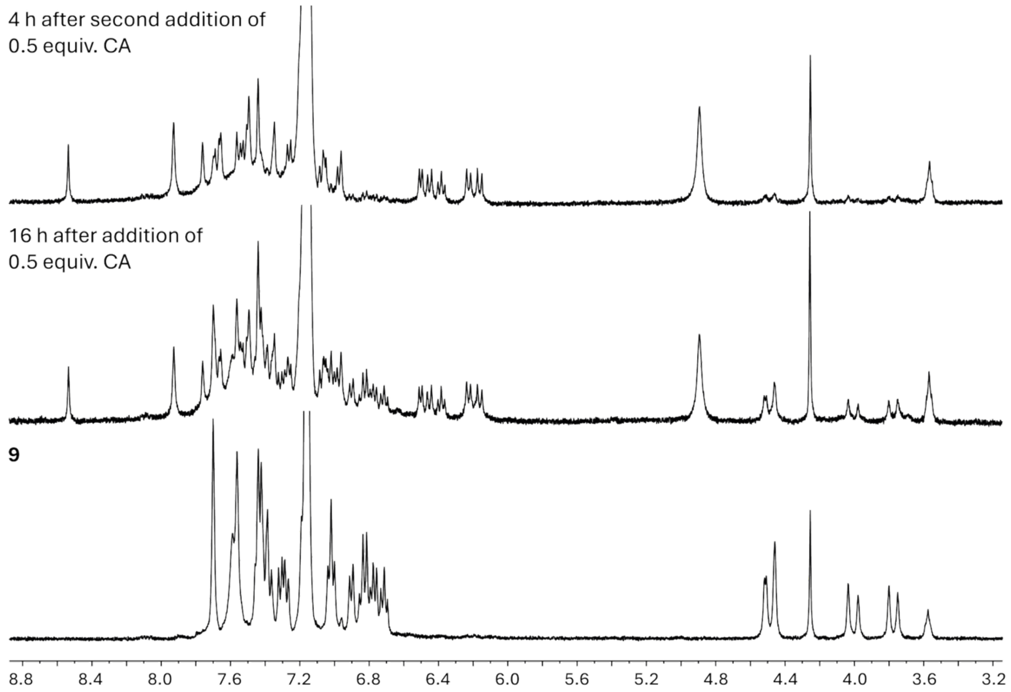

Figure S4. ^{1}H NMR spectra of **9** in benzene-d_6 before (bottom) and 16 h after the addition of 0.5 equivalents of *p*-chloranil (middle). The top spectrum shows the reaction progress after addition of another 0.5 equivalents of *p*-chloranil 4 h after the second addition (*x*-axis in δ[ppm]).

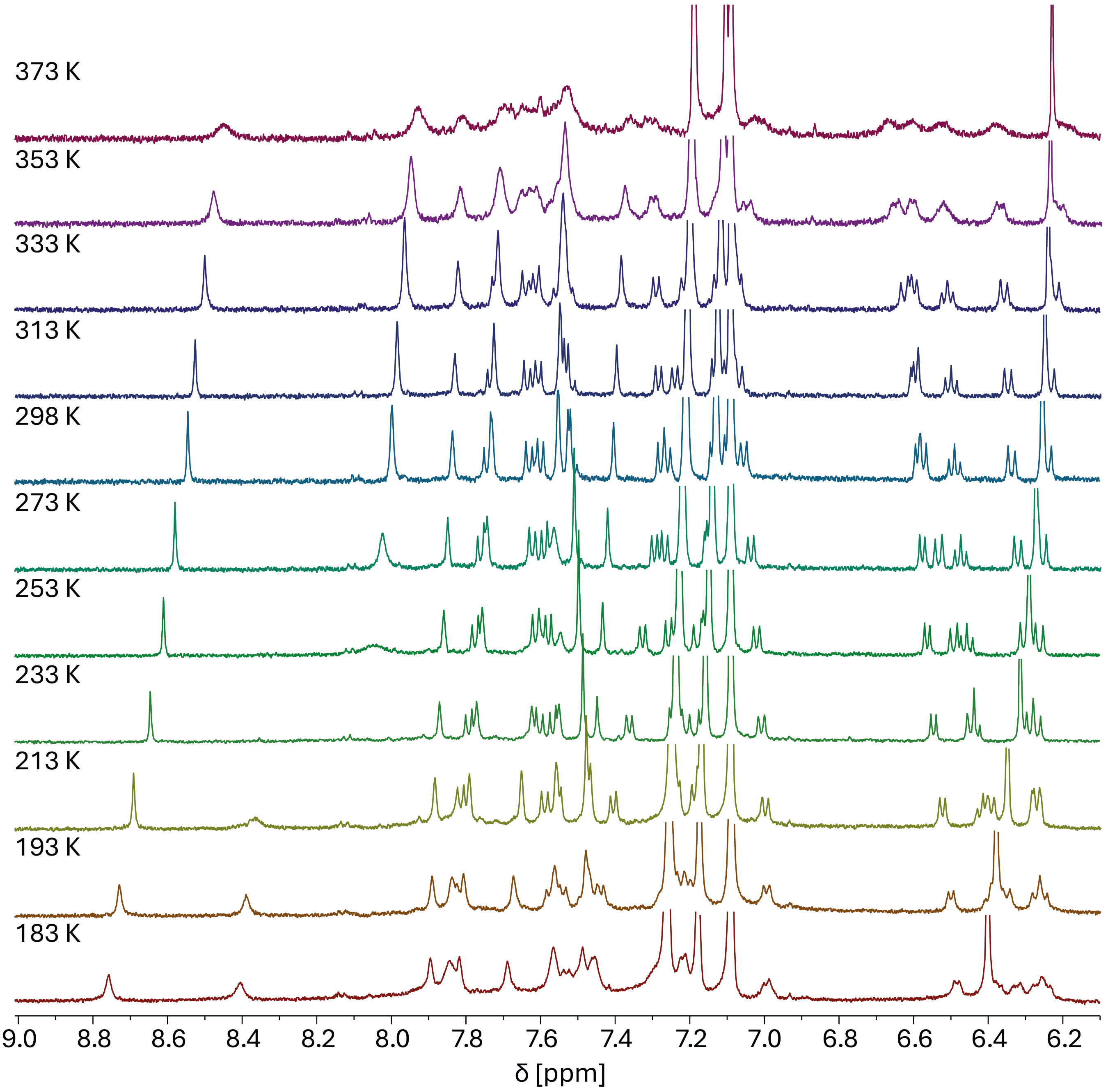

Figure S5. Supplementary figure to Figure 4. ^{1}H NMR spectra of *c*-**MNC** in toluene-d_8 at variable temperatures (183–373 K). The room-temperature measurements after cooling and heating look the same as the first room-temperature measurement before heating/cooling. The signal associated with reduced *p*-chloranil is absent. Raw data of these spectra are uploaded on Zenodo. The link can be found on the first page of the Supporting Information.

2. Synthesis

Materials and Instrumentation

All chemicals and solvents were purchased from commercial sources and were used without further purification unless stated otherwise. The reactions and experiments that are sensitive to dioxygen were performed using Schlenk techniques and deoxygenated solvents under nitrogen atmosphere. All reactions that require heating reported here were heated with metal heating mantles. The NMR experiments were performed on Bruker NMR spectrometers operating at 400, 500, or 600 MHz proton frequencies. Standard pulse sequences were used, and the data were processed using twofold zero-filling in the indirect dimension for all 2D experiments. Chemical shifts (δ) are reported in parts per million (ppm) relative to the solvent residual peak (^{1}H and ^{13}C NMR, respectively): $CDCl_3$ (δ = 7.26 and 77.16 ppm), CD_2Cl_2 (δ = 5.32 and 53.84 ppm), $(CD_3)_2CO$ (δ = 2.05 and 29.84 ppm), C_6D_6 (δ = 7.16 and 128.06 ppm), and toluene-d_8 (δ = 6.97 and 20.43 ppm).[1] Structural assignments were made with additional information from gCOSY, gHSQC, gNOESY, and gHMBC experiments. High-resolution mass spectra (HRMS) were measured as HR-electrospray ionization (ESI)-MS, HR-atmospheric pressure chemical ionization (APCI)-MS (on a timsTOF Pro, Bruker/Vanquish, Thermo Scientific and QExactive and Ultimate 3000, Thermo Scientific), or HR-electron ionization (EI)-MS (on a double-focusing (BE geometry) magnetic sector mass spectrometer DFS, Thermo Scientific). The EPR spectra were recorded in a nitrogen-saturated toluene solution on an X-band bench-top EPR spectrometer Magnettech ESR5000 from Bruker (9.48 GHz) using the following instrumental parameters: magnetic field 336–341 mT, scan time 60 s, modulation amplitude 0.01 mT, microwave power 1 dB. The UV–vis measurements were performed on an Agilent 8453 spectrophotometer equipped with Unisoku CoolSpeK USP-203-B thermostat under dry nitrogen atmosphere and using spectroscopy-grade toluene. Purification by recycling gel permeation chromatography (GPC) was performed with a LC-20AR from Shimadzu with chloroform as solvent. Light-promoted reactions were performed in an immersion-well photoreactor equipped with a quartz cooling system and quartz immersion tube containing a 150 W medium pressure mercury lamp. The lamp is a DQ-lamp produced by Noblelight® with the wavelengths of the emission spectrum shown below (Figure S6). The immersion tube containing the lamp has a diameter of 3 cm and is surrounded by a water-cooling system during the reaction, with the entire assembly immersed in the reaction solution.

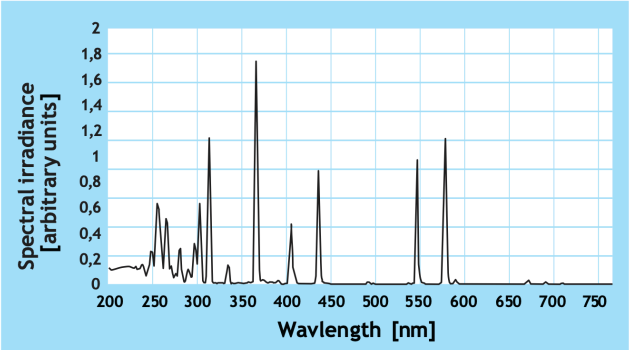

Figure S6. Emission spectrum of the DQ-medium pressure lamp used for the light-promoted reactions. Image source: https://www.noblelight.com/media/media/hng/doc_hng/products_and_solutions_1/uv_lamps_and_systems_1/Medium_Pressure_UV_Lamps_B114E_190805.pdf

The raw NMR, EPR, UV–vis, and IR data are available free of charge on a public repository Zenodo (DOI: 10.5281/zenodo.17350538).

Methyl 3-(2-formyl-4-methylphenyl)propanoate (10)

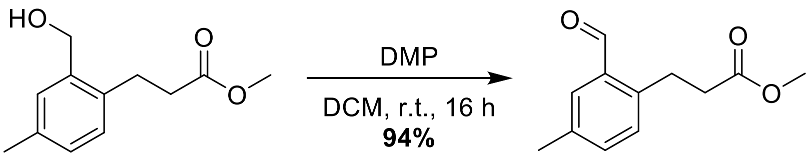

Methyl 3-(2-(hydroxymethyl)-4-methylphenyl)propanoate (727 mg, 3.49 mmol, 1.00 equiv) was dissolved in CH_2Cl_2 (25 mL), and Dess–Martin periodinane (DMP; 1.48 g, 3.49 mmol, 1.00 equiv) was added. The mixture was stirred for 16 h and was afterwards quenched with a saturated solution of $NaHCO_3$ (100 mL). The organic phase was washed with a saturated solution of $Na_2S_2O_3$ (100 mL) and water (100 mL), and was dried over $MgSO_4$. After evaporating the solvent *in vacuo*, the crude product was purified via column chromatography over silica gel using CH_2Cl_2/EtOAc (1:1) as an eluent. The product was isolated as a yellow oil.

Yield: 678 mg (3.29 mmol, 94%)

^{1}H NMR (298 K, 400 MHz, $CDCl_3$): δ = 10.17 (s, 1H), 7.61 (d, $^4J_{HH}$ = 2.0 Hz, 1H), 7.33 (dd, $^3J_{HH}$ = 7.7 Hz, $^4J_{HH}$ = 2.0 Hz, 1H), 7.21 (d, $^3J_{HH}$ = 7.7 Hz, 1H), 3.65 (s, 3H), 3.31 (t, $^3J_{HH}$ = 7.6 Hz, 2H), 2.64 (t, $^3J_{HH}$ = 7.6 Hz, 2H), 2.40 (s, 3H) ppm.

^{13}C{^{1}H} NMR (298 K, 101 MHz, $CDCl_3$): δ = 193.0, 173.3, 140.0, 137.0, 134.8, 134.1, 133.8, 131.3, 51.7, 35.6, 27.8, 21.0 ppm.

HRMS (ESI): *m/z* [*M* + H]$^+$ Calcd for $C_{12}H_{15}O_3$ 207.1021; Found 207.1018.

IR: 2952, 1734, 1687, 1568, 1436, 1241, 1194, 1156, 1035, 932, 830, 777, 667, 552, 428 cm^{-1}.

Methyl 3-(4-methyl-2-vinylphenyl)propanoate (4)

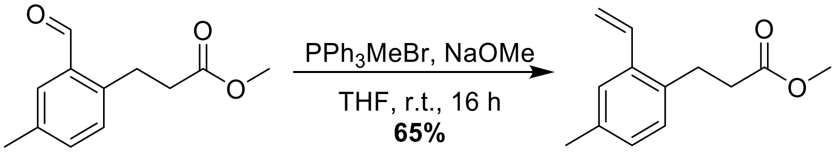

Methyltriphenylphosphonium bromide (1.18 g, 3.29 mmol, 1.00 equiv) was dissolved in a flame-dried flask under nitrogen atmosphere in anhydrous THF (150 mL) before NaOMe (5.4 M in MeOH, 0.73 mL, 3.95 mmol, 1.20 equiv) was added dropwise. The suspension was stirred for 1 h at room temperature. Methyl 3-(2-formyl-4-methylphenyl)propanoate (678 mg, 3.29 mg, 1.00 equiv) dissolved in THF (5 mL) was added and the mixture was stirred for 16 h. The solution was quenched with aqueous HCl (5 M, 100 mL) before the organic phase was washed with water (3 × 100 mL) and brine (100 mL), and was dried over $MgSO_4$. After evaporating the solvent *in vacuo* the crude product was purified via column chromatography over silica gel with CH_2Cl_2/EtOAc (5:2) as an eluent. After evaporation of the solvent, the desired product was obtained as a colorless oil.

Yield: 435 mg (2.13 mmol, 65%)

^{1}H NMR (298 K, 400 MHz, $CDCl_3$): δ 7.31 (d, $^4J_{HH}$ = 1.8 Hz, 1H), 7.06 (d, $^3J_{HH}$ = 7.8 Hz, 1H), 7.03 (dd, $^3J_{HH}$ = 7.8 Hz, $^4J_{HH}$ = 1.8 Hz, 1H), 6.96 (dd, $^3J_{HH}$ = 17.3 Hz, $^3J_{HH}$ = 11.0 Hz, 1H), 5.65 (dd, $^3J_{HH}$ = 17.3 Hz, $^4J_{HH}$ = 1.4 Hz, 1H), 5.30 (dd, $^3J_{HH}$ = 11.0 Hz, $^4J_{HH}$ = 1.4 Hz, 1H), 3.68 (s, 3H), 3.03–2.94 (m, 2H), 2.60–2.51 (m, 2H), 2.33 (s, 3H) ppm.

^{13}C{^{1}H} NMR (298 K, 101 MHz, $CDCl_3$): δ 173.5, 136.5, 136.4, 134.9, 134.4, 129.4, 128.9, 126.7, 115.9, 51.7, 35.4, 28.2, 21.2 ppm.

HRMS (ESI): *m/z* $[M + H]^+$ Calcd for $C_{13}H_{17}O_2$ 205.1228; Found 205.1225.

IR: 2950, 1736, 1494, 1435, 1195, 1161, 988, 912, 819, 657, 541, 424 cm^{-1}.

6-Bromophenanthrene-3-carbaldehyde (1)

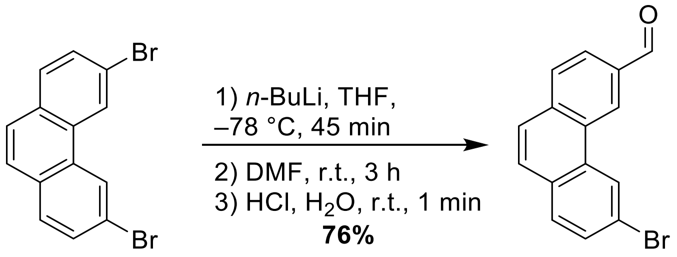

In a flame-dried flask, 3,6-dibromophenantrene (601 mg, 1.79 mmol, 1.00 equiv) was dissolved in dry THF (40 mL) and the solution was cooled to –78 °C. *n*-Butyllithium (0.72 mL, 1.9 M in Et_2O, 1.79 mmol, 1.00 equiv) was added and the mixture was stirred for 45 min. DMF (0.21 mL, 2.69 mmol, 1.50 equiv) was added, the mixture was warmed to room temperature and was stirred for another 3 h. The solution was poured into aqueous HCl (2 M, 50 mL) and the aqueous phase was extracted with CH_2Cl_2 (3 × 50 mL). The combined organic phases were dried over $MgSO_4$ and the residue was purified via column chromatography over silica gel using CH_2Cl_2 as an eluent. After evaporation of the solvent, the desired product was obtained as a colorless solid.

Yield: 386 mg (1.35 mmol, 76%)

^{1}H NMR (298 K, 400 MHz, $CDCl_3$): δ 10.28 (s, 1H), 9.07 (d, $^4J_{HH}$ = 0.8 Hz, 1H), 8.89 (dd, $^4J_{HH}$ = 1.7 Hz, $^4J_{HH}$ = 0.8 Hz, 1H), 8.11 (dd, $^3J_{HH}$ = 8.2 Hz, $^4J_{HH}$ = 1.5 Hz, 1H), 8.01 (d, $^3J_{HH}$ = 8.2 Hz, 1H), 7.86 (d, $^3J_{HH}$ = 8.2 Hz, 1H), 7.83–7.78 (m, 2H), 7.76 (dd, $^3J_{HH}$ = 8.2 Hz, $^4J_{HH}$ = 1.7 Hz, 1H) ppm.

^{13}C{^{1}H} NMR (298 K, 101 MHz, $CDCl_3$): δ 192.3, 136.3, 134.6, 132.0, 130.9, 130.8, 130.5, 129.84, 129.78, 129.2, 127.2, 127.0, 125.9, 125.8, 122.1 ppm.

The spectral data are in accordance with those previously reported in the literature.[2]

Methyl (*E*)-3-(2-(2-(6-bromophenanthren-3-yl)vinyl)phenyl) propanoate (3)

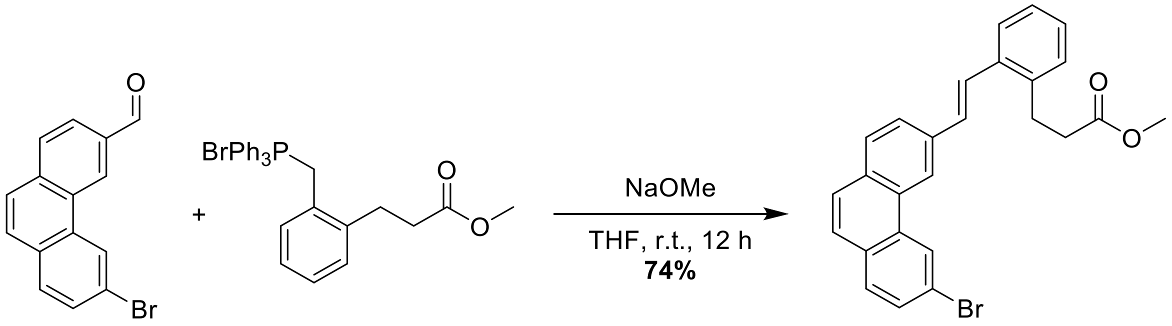

In a flame-dried flask, finely ground (2-(3-methoxy-3-oxopropyl)benzyl)triphenylphosphonium bromide (857 mg, 1.65 mmol, 1.00 equiv) was mixed with anhydrous THF (20 mL) and sodium methoxide (5 M in MeOH, 0.39 mL, 1.98 mmol, 1.20 equiv) was added. The suspension was stirred for 1 h at room temperature. 6-Bromophenanthrene-3-carbaldehyde (470 mg, 1.65 mmol, 1.00 equiv) was added at once as a ground powder and the mixture was stirred for 12 h. The orange color was slowly fading over time. The solution was quenched with aqueous HCl (5 M, 50 mL) before CH_2Cl_2 was added (100 mL) and the organic phase was washed with water (3 × 30 mL) and brine (50 mL), and was dried over $MgSO_4$. The crude product was purified via column chromatography over silica gel, first using CH_2Cl_2/EtOAc (2.5:1) and then CH_2Cl_2 as eluents. The product was obtained as a mixture of *E*- and *Z*-isomers.
The isomeric mixture was dissolved in toluene (20 mL). Iodine was added and the solution was stirred for three days at 100 °C to obtain the pure *E*-product. The iodine was quenched with a saturated solution of $Na_2S_2O_3$ and the solvent was evaporated under reduced pressure. The desired product was isolated as a colorless oil.

Yield: 547 mg (1.23 mmol, 74%)

^{1}H NMR (298 K, 400 MHz, $CDCl_3$): δ 8.85 (d, $^3J_{HH}$ = 1.8 Hz, 1H), 8.59 (s, 1H), 7.88 (s, 1H), 7.83–7.78 (m, 3H), 7.69 (dd, $^3J_{HH}$ = 8.5 Hz, $^4J_{HH}$ = 1.8 Hz, 1H), 7.66 (d, $^3J_{HH}$ = 8.8 Hz, 1H), 7.59 (d, $^3J_{HH}$ = 16.1 Hz, 1H), 7.35–7.23 (m, 4H), 3.72 (s, 3H), 3.20 (dd, $^3J_{HH}$ = 8.7 Hz, $^3J_{HH}$ = 7.1 Hz, 2H), 2.69 (dd, $^3J_{HH}$ = 8.7 Hz, $^3J_{HH}$ = 7.1 Hz, 2H) ppm.
In the section Copies of NMR spectra on page S23, there is also a ^{1}H NMR spectrum of the mixture of the isomers for comparison.

^{13}C{^{1}H} NMR (298 K, 101 MHz, $CDCl_3$): δ 173.5, 138.5, 136.2, 136.1, 131.9, 131.8, 130.99, 130.96, 130.2, 123.0, 129.6, 129.6, 129.1, 128.1, 127.2, 127.1, 126.7, 126.4, 126.1, 125.7, 124.9, 121.9, 121.0, 51.9, 35.5, 28.8 ppm.

HRMS (ESI): *m*/*z* [*M* + Na]$^+$ Calcd for $C_{26}H_{21}BrNaO_2$ 467.0623; Found 467.0620.

IR: 2947, 1731, 1590, 1434, 1253, 1236, 1195, 1157, 1101, 1075, 1029, 957, 878, 780, 631, 594, 544, 499, 423 cm^{-1}.

Dimethyl 3,3'-(((1*E*,1'*E*)-phenanthrene-3,6-diylbis(ethene-2,1-diyl))bis(2,1-phenylene))di-propionate methyl (5)

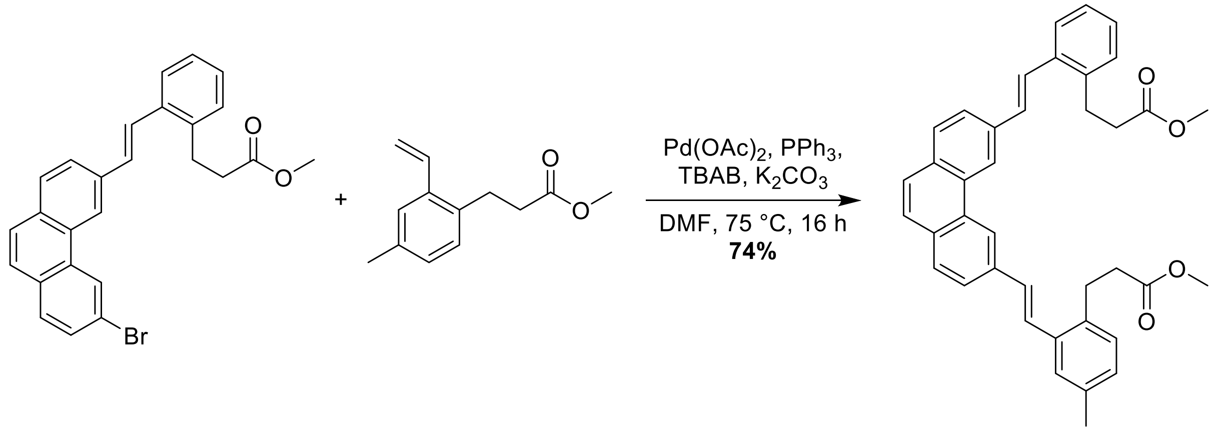

In a flame-dried flask, methyl (*E*)-3-(2-(2-(6-bromophenanthren-3-yl)vinyl)phenyl)propanoate (33 mg, 74 µmol, 1.0 equiv), palladium(II) acetate (1.7 mg, 7.4 µmol, 0.10 equiv), triphenylphosphine (2.9 mg, 0.011 mmol, 0.15 equiv), tetrabutylammonium bromide (TBAB; 24 mg, 74 µmol, 1.0 equiv), potassium carbonate (10 mg, 74 µmol, 1.0 equiv), and methyl 3-(4-methyl-2-vinylphenyl)propanoate (15 mg, 74 µmol, 1.0 equiv) were dissolved in deoxygenated DMF (10 mL) under nitrogen atmosphere. The mixture was stirred at 75 °C for 16 h. The solution was poured into aqueous HCl (5 M, 20 mL), and CH_2Cl_2 (30 ml) was added. The organic phase was washed with water (4 × 100 mL) and an aqueous LiCl solution (5 %, 30 mL), and the solvent was removed *in vacuo*. The crude product was purified via column chromatography over silica gel using CH_2Cl_2 as an eluent. Subsequently, the product was purified via recycling gel permeation chromatography (GPC) with $CHCl_3$ as an eluent. The product was isolated as a yellow oil.

Yield: 31 mg (0.055 mmol, 74 %). It is important not to exceed 80 °C during the reaction to avoid dehalogenation of the starting material.

^{1}H NMR (298 K, 400 MHz, $CDCl_3$): δ 8.89–8.82 (m, 2H), 7.98–7.92 (m, 4H), 7.82 (dd, $^3J_{HH}$ = 7.3 Hz, $^3J_{HH}$ = 1.5 Hz, 1H), 7.80–7.77 (m, 2H), 7.69 (d, $^3J_{HH}$ = 6.8 Hz, 1H), 7.67–7.63 (m, 2H), 7.44 (d, $^4J_{HH}$ = 3.1 Hz, 1H), 7.40 (d, $^4J_{HH}$ = 3.1 Hz, 1H), 7.39–7.35 (m, 1H), 7.35–7.31 (m, 2H), 7.22 (d, $^3J_{HH}$ = 7.8 Hz, 1H), 7.18–7.12 (m, 1H), 3.75 (s, 3H), 3.74 (s, 3H), 3.31–3.20 (m, 4H), 2.79–2.70 (m, 4H), 2.41 (s, 3H) ppm.

^{13}C{^{1}H} NMR (298 K, 101 MHz, $CDCl_3$): δ 173.6, 173.5, 138.5, 136.5, 136.3, 136.13, 136.00, 135.98, 135.91, 135.6, 132.12, 132.08, 131.4, 131.0, 130.7, 129.7, 129.6, 129.2, 128.9, 128.0, 127.1, 126.9, 126.8, 126.7, 126.5, 126.4, 126.2, 124.4, 124.3, 122.1, 122.0, 51.8, 35.7, 35.5, 28.8, 28.5, 21.3 ppm.
Two signals are not resolved due to signal overlap.

HRMS (ESI): *m/z* [*M* + H]$^+$ Calcd for $C_{39}H_{36}O_4$ 569.2692; Found 569.2689.

IR: 2948, 1730, 1603, 1434, 1195, 1158, 958, 839, 750, 634, 613, 553, 522, 427 cm^{-1}.

Dimethyl 3,3'-(1-methylbenzo[1,2-*c*:4,3-*c*']diphenanthrene-4,15-diyl)dipropionate (6)

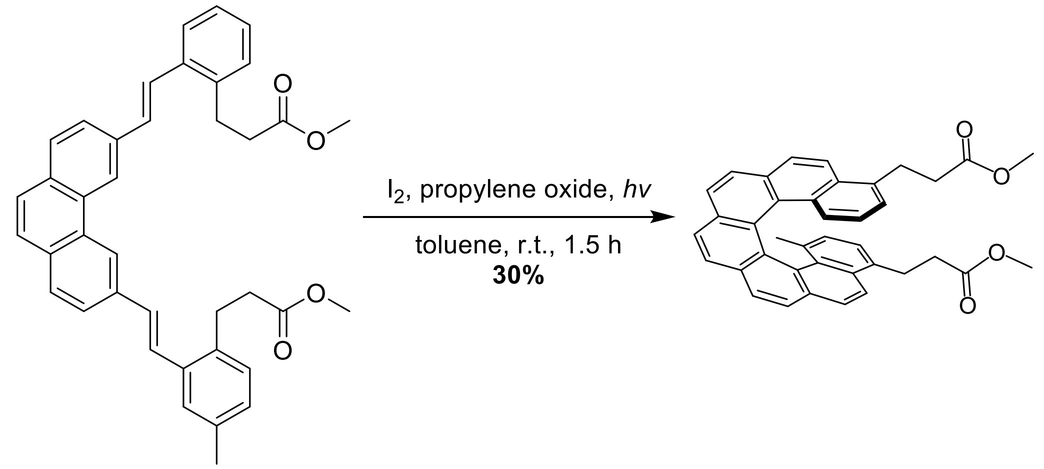

Iodine (271 mg, 1.04 mmol, 2.20 equiv), propylene oxide (16.6 mL, 237 mmol, 500 equiv), and methyl 3-(2-((*E*)-2-(6-((*E*)-2-(3-methoxy-3-oxopropyl)-5-methylstyryl)phenanthren-3-yl)vinyl)phenyl)propanoate (270 mg, 0.475 mmol, 1.00 equiv) were dissolved in toluene (650 mL). The solution was bubbled with argon for 30 min before it was irradiated in an immersion-well photoreactor equipped with a quartz cooling system and quartz immersion tubes containing 150 W medium-pressure mercury lamp for 1.5 h. The solution was quenched with a saturated aqueous $Na_2S_2O_3$ (50 mL), washed with water (2 × 50 mL) and brine (50 mL), and was dried over $MgSO_4$. The crude product was purified via column chromatography over silica gel using CH_2Cl_2/EtOAc (80:1 to 60:1). After evaporation of the solvent, the desired product was obtained as a yellow solid.

Yield: 152 mg (0.269 mmol, 57%). Yields were varying between 30 and 57%.

^{1}H NMR (298 K, 400 MHz, $CDCl_3$): δ 8.05–7.99 (m, 2H), 7.98 (s, 1H), 7.96–7.90 (m, 2H), 7.83 (d, $^3J_{HH}$ = 8.1 Hz, 1H), 7.69–7.63 (m, 2H), 7.60–7.54 (m, 2H), 6.81 (d, $^4J_{HH}$ = 2.7 Hz, 1H), 6.79 (s, 1H), 6.67 (d, $^3J_{HH}$ = 7.32 Hz, 1H), 6.30–6.24 (m, 2H), 3.75 (s, 3H), 3.75 (s, 3H), 3.23–3.05 (m, 3H), 3.04–2.93 (m, 1H), 2.66–2.47 (m, 4H), 0.76 (s, 3H) ppm.

^{13}C{^{1}H} NMR (298 K, 101 MHz, $CDCl_3$): δ 173.72, 173.68, 134.8, 132.3, 132.2, 131.7, 131.6, 131.4, 130.5, 130.35, 130.30, 129.6, 129.4, 127.9, 127.4, 127.2, 127.1, 126.9, 126.80, 126.76, 126.54, 126.53, 126.45, 126.0, 125.9, 125.1, 124.9, 124.6, 123.8, 122.9, 122.7, 122.3, 51.88, 51.86, 35.6, 35.3, 28.6, 28.2, 21.2 ppm.

HRMS (ESI) *m/z* [*M* + Na]$^+$ Calcd for $C_{39}H_{32}NaO_4$ 587.2193; Found 587.2200.

IR: 2948, 1730, 1434, 1194, 1165, 842, 818, 776, 670, 613, 521, 449 cm^{-1}.

Melting point: 84–86 °C

1-Methyl-4,5,16,17-tetrahydrobenzo[*no*]benzo[8,9]anthra[1,2-*a*]tetraphene-6,15-dione (7)

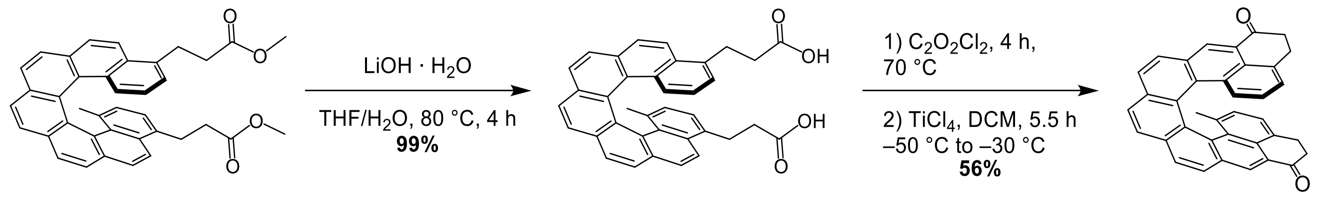

Dimethyl 3,3'-(1-methylbenzo[1,2-*c*:4,3-*c*']diphenanthrene-4,15-diyl)dipropionate (105 mg, 0.186 mmol, 1.00 equiv) was dissolved in a solvent mixture of THF and water (30 mL, 3:1). LiOH monohydrate (109 mg, 1.86 mmol, 10 equiv) was added and the mixture was stirred at 80 °C for 4 h. The solvent was concentrated to 5 mL under reduced pressure and aqueous HCl (5 M, 10 mL) was added. The yellow precipitate was filtered off, washed with water, and dried *in vacuo* for 30 min. The product was used without further purification in the next step.

^{1}H NMR (298 K, 400 MHz, acetone-d_6): δ 8.18–8.13 (m, 2H), 8.12–8.03 (m, 3H), 7.96 (d, $^3J_{HH}$ = 8.2 Hz, 1H), 7.80 (d, $^3J_{HH}$ = 8.5 Hz, 2H), 7.72 (d, $^3J_{HH}$ = 8.8 Hz, 2H), 6.91 (d, $^4J_{HH}$ = 6.6 Hz, 1H), 6.80 (d, $^3J_{HH}$ = 7.3 Hz, 1H), 6.74 (d, $^3J_{HH}$ = 8.5 Hz, 1H), 6.34–6.26 (m, 2H), 3.25–2.96 (m, 4H), 2.69–2.51 (m, 4H), 2.09 (s, 2H), 0.74 (s, 3H) ppm.

The crude acid (99 mg, 0.18 mmol, 1.00 equiv) was dissolved in oxalyl chloride (10 mL) under nitrogen atmosphere and stirred for 4 h at 70 °C before the excess of oxalyl chloride was evaporated under reduced pressure and the residue was dried under high vacuum for 30 min. Afterwards, it was redissolved in anhydrous CH_2Cl_2 (60 mL), cooled to –50 °C, and titanium tetrachloride (0.20 mL, 1.8 mmol, 10 equiv) was added. The mixture turned black, was let to warm up to –30 °C, and stirred for 5.5 h at this temperature. The mixture was poured into water, the organic phase was washed with water (3 × 40 mL) and brine (1 × 40 mL), and dried over $MgSO_4$. The solvent was removed *in vacuo* and the crude product was purified via column chromatography over silica gel using CH_2Cl_2 as eluent. After evaporation of the solvent, the desired product was isolated as a yellow solid.

Yield: 51.2 mg (0.102 mmol, 56% over two steps)

^{1}H NMR (298 K, 400 MHz, $CDCl_3$): δ 8.42–8.36 (m, 2H), 8.09–7.99 (m, 4H), 7.98–7.90 (m, 2H), 6.86 (dd, $^3J_{HH}$ = 7.0 Hz, $^4J_{HH}$ = 1.1 Hz, 1H), 6.74 (d, $^3J_{HH}$ = 7.3 Hz, 1H), 6.69 (dd, $^3J_{HH}$ = 8.5 Hz, $^4J_{HH}$ = 1.0 Hz, 1H), 6.35–6.27 (m, 2H), 3.22–2.94 (m, 4H), 2.94–2.71 (m, 4H), 0.73 (s, 3H) ppm.

^{13}C{^{1}H} NMR (298 K, 101 MHz, $CDCl_3$): δ 198.74, 198.57, 133.3, 131.9, 131.7, 131.2, 130.91, 130.89, 130.6, 129.44, 129.38, 129.29, 128.7, 128.5, 128.4, 128.1, 128.0, 127.94, 127.84, 127.8, 127.6, 127.4, 126.5, 126.0, 125.6, 125.5, 124.4, 123.9, 123.6, 121.8, 38.65, 38.57, 28.7, 28.3, 20.8 ppm.
Two signals were not resolved due to signal overlap.

HRMS (ESI) *m/z* [*M* + Na]$^+$ Calcd for $C_{37}H_{24}NaO_2$ 523.1674; Found 523.1662.

IR: 2921, 2852, 1679, 1590, 1485, 1438, 1377, 1316, 1241, 1159, 1136, 839, 816, 760, 679, 606, 580, 541, 520 cm^{-1}.

Melting point: decomposition >280 °C

1-Methyl-7,14-dihydrobenzo[*no*]benzo[8,9]anthra[1,2-*a*]tetraphene (8)

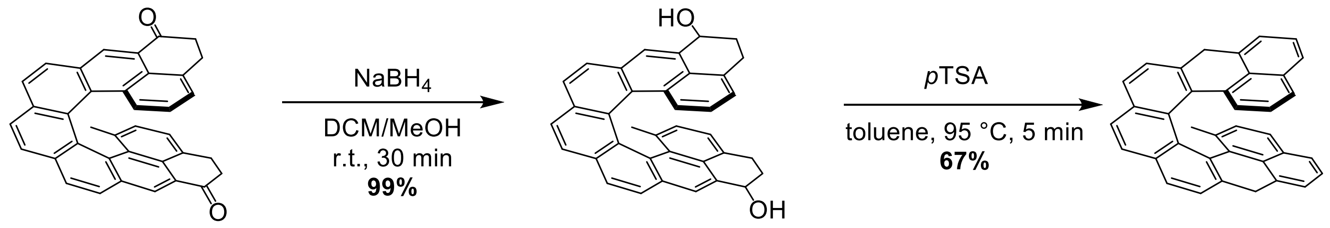

1-Methyl-4,5,16,17-tetrahydrobenzo[*no*]benzo[8,9]anthra[1,2-*a*]tetraphene-6,15-dione (24.0 mg, 48.1 µmol, 1.00 equiv) was dissolved in a solvent mixture of CH_2Cl_2/MeOH (20 mL, 1:1), and the solution was cooled to 0 °C. Sodium borohydride (18 mg, 0.48 mmol, 10 equiv) was added, and the solution was warmed up to room temperature and stirred for 30 min at the same temperature. The mixture was poured into aqueous HCl (5 M, 20 mL), the organic phase was washed with water (3 × 20 mL) and brine (1 × 20 mL), and was dried over $MgSO_4$.

The crude product was dissolved in deoxygenated toluene (15 mL) under nitrogen atmosphere and the solution was heated at 95 °C. *p*-Toluenesulfonic acid (55 mg, 0.29 mmol, 6.0 equiv) was added, and the solution turned from yellow to dark red. The mixture was stirred for 5 min, and was then filtered over a pad of silica gel and washed with toluene (50 mL). The solvent was removed *in vacuo* and the crude product was purified first via column chromatography over silica gel using cyclohexane/EtOAc (20:1) as an eluent and then via recycling gel permeation chromatography (GPC) using $CHCl_3$ as an eluent. After evaporation of the solvent, the desired product was isolated as a yellow solid.

Yield: 15 mg (0.32 mmol, 67% over two steps)

^{1}H NMR (298 K, 500 MHz, CD_2Cl_2): δ 7.74 (d, $^3J_{HH}$ = 7.8 Hz, 1H), 7.67–7.61 (m, 2H), 7.59 (d, $^3J_{HH}$ = 8.4 Hz, 1H), 7.50 (dd, $^3J_{HH}$ = 7.8 Hz, 1H), 7.34 (d, $^3J_{HH}$ = 7.6 Hz, 1H), 7.29 (dd, $^3J_{HH}$ = 8.1 Hz, $^3J_{HH}$ = 6.8 Hz, 1H), 7.21 (d, $^3J_{HH}$ = 7.7 Hz, 1H), 7.18 (d, $^3J_{HH}$ = 7.6 Hz, 1H), 7.13 (d, $^3J_{HH}$ = 6.8 Hz, 1H), 7.10 (d, $^3J_{HH}$ = 6.8 Hz, 1H), 7.03 (d, $^3J_{HH}$ = 8.1 Hz, 1H), 6.67 (d, $^3J_{HH}$ = 7.4 Hz, 1H), 6.57 (d, $^3J_{HH}$ = 8.1 Hz, 1H), 6.50 (d, $^3J_{HH}$ = 8.3 Hz, 1H), 6.41 (d, $^3J_{HH}$ = 8.3 Hz, 1H), 6.30 (dd, $^3J_{HH}$ = 7.7 Hz, $^3J_{HH}$ = 7.7 Hz, 1H), 4.45 (d, $^3J_{HH}$ = 22.9 Hz, 1H), 4.35 (d, $^3J_{HH}$ = 22.9 Hz, 1H), 4.25 (d, $^3J_{HH}$ = 20.6 Hz, 1H), 4.11 (d, $^3J_{HH}$ = 20.6 Hz, 1H), 1.09 (s, 3H) ppm.

^{13}C{^{1}H} NMR (298 K, 126 MHz, CD_2Cl_2): δ 136.8, 134.6, 133.6, 133.6, 132.6, 132.5, 132.2, 132.0, 131.9, 131.7, 131.5, 131.5, 130.9, 130.0, 129.2, 129.0, 128.3, 127.3, 127.1, 126.9, 126.8, 126.6, 126.4, 125.8, 125.5, 125.3, 125.3, 125.3, 124.8, 124.7, 124.5, 122.7, 122.4, 122.2, 36.2, 35.1, 20.1 ppm.

HRMS (APCI): *m*/*z* [*M* + H]$^+$ Calcd for $C_{37}H_{25}$ 469.1956; Found 469.1951.

6,15-bis(3,5-di-*tert*-butylphenyl)-1-methyl-7,14-dihydrobenzo[*no*]benzo[8,9]anthra[1,2-*a*]tetraphene (9)

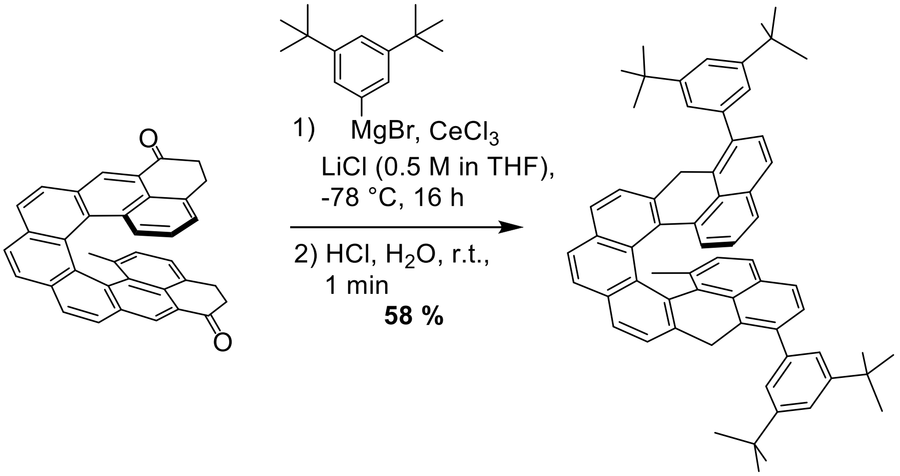

In a flame-dried flask, 1-methyl-4,5,16,17-tetrahydrobenzo[*no*]benzo[8,9]anthra[1,2-*a*]tetraphene-6,15-di-one (15.7 mg, 0.0314 mmol, 1.00 equiv) and $CeCl_3$ (11.6 mg, 0.0470 mmol, 1.50 equiv) were dissolved in a solution of LiCl in THF (0.5 M, 3 mL) and left to react for 1 h at room temperature. Afterwards, the reaction mixture was cooled to –78 °C with a dry ice/acetone bath, to which freshly prepared 1-bromo-3,5-di-*tert*-butylbenzene (0.2 M in THF, 1.3 mL, 5.0 equiv) was added. The reaction mixture was warmed up to room temperature and stirred for 16 h. The mixture was poured into aqueous HCl (5 M), the aqueous phases were extracted with CH_2Cl_2 (3 x 10 mL), and the combined organic phases were dried over anhydrous $MgSO_4$ and concentrated *in vacuo*. Purification of the crude product was done via inert column chromatography under nitrogen atmosphere over silica using deoxygenated cyclohexane/CH_2Cl_2 (1:1) as an eluent. Subsequently, the product was purified via recycling gel permeation chromatography (GPC) with $CHCl_3$ as an eluent to afford the product as a yellow solid.

Yield: 15.3 mg (0.0181 mmol, 58%)

^{1}H NMR (298 K, 500 MHz, CD_2Cl_2): δ 7.71 (d, $^3J_{HH}$ = 7.8 Hz, 1H), 7.65 (d, $^3J_{HH}$ = 8.3 Hz, 1H), 7.61 (d, $^3J_{HH}$ = 7.8 Hz, 1H), 7.58 (d, $^3J_{HH}$ = 8.3 Hz, 1H), 7.54–7.51 (m, 2H), 7.38 (d, $^3J_{HH}$ = 7.8 Hz, 1H), 7.36–7.33 (m, 4H), 7.33–7.30 (m, 2H), 7.25–7.21 (m, 2H), 7.14 (d, $^3J_{HH}$ = 8.2 Hz, 1H), 6.75 (d, $^3J_{HH}$ = 8.0 Hz, 1H), 6.71 (d, $^3J_{HH}$ = 7.3 Hz, 1H), 6.63 (d, $^3J_{HH}$ = 8.2 Hz, 1H), 6.59 (d, $^3J_{HH}$ = 8.2 Hz, 1H), 6.47 (dd, $^3J_{HH}$ = 7.8 Hz, $^3J_{HH}$ = 7.8 Hz, 1H), 4.48–4.33 (m, 2H), 4.15 (d, $^3J_{HH}$= 22.9 Hz, 1H), 3.89 (d, $^3J_{HH}$= 20.6 Hz, 1H), 1.49–1.43 (m, 36H), 1.09 (s, 3H) ppm.

^{13}C{^{1}H} NMR (298 K, 126 MHz, CD_2Cl_2): 151.4, 151.2, 141.7, 141.6, 137.28, 137.26, 136.9, 134.8, 133.5, 132.9, 132.5, 132.4, 132.2, 132.1, 131.8, 131.2, 131.0, 130.5, 129.2, 129.1, 128.9, 128.5, 128.3, 127.9, 127.2, 127.1, 126.94, 126.87, 126.8, 126.5, 126.4, 125.7, 125.6, 125.5, 125.4, 125.0, 124.5, 124.3, 123.9, 122.9, 121.2, 121.1, 35.33, 35.30, 34.5, 34.1, 31.8, 20.2 ppm.
One signal was not resolved due to signal overlap. See Figure S10 for details.

HRMS (APCI): *m/z* [*M* + H]$^+$ Calcd for $C_{65}H_{65}$ 845.5081; Found 845.5088.

IR: 2955, 2923, 2863, 1592, 1592, 1361, 1263, 877, 826, 715, 539 cm^{-1}.

Melting point: 130 °C

(16a*S*,16b*S*)-3,12-Bis(3,5-di-*tert*-butylphenyl)-16a-methyl-16a*H*-dibenzo[*cd*,*ghi*]phenaleno[2,1-*p*]perylene (c-MNC, left) and 6,15-bis(3,5-di-*tert*-butylphenyl)-1-methylbenzo[*no*]benzo[8,9]anthra[1,2-*a*]tetraphene (MNC, right)

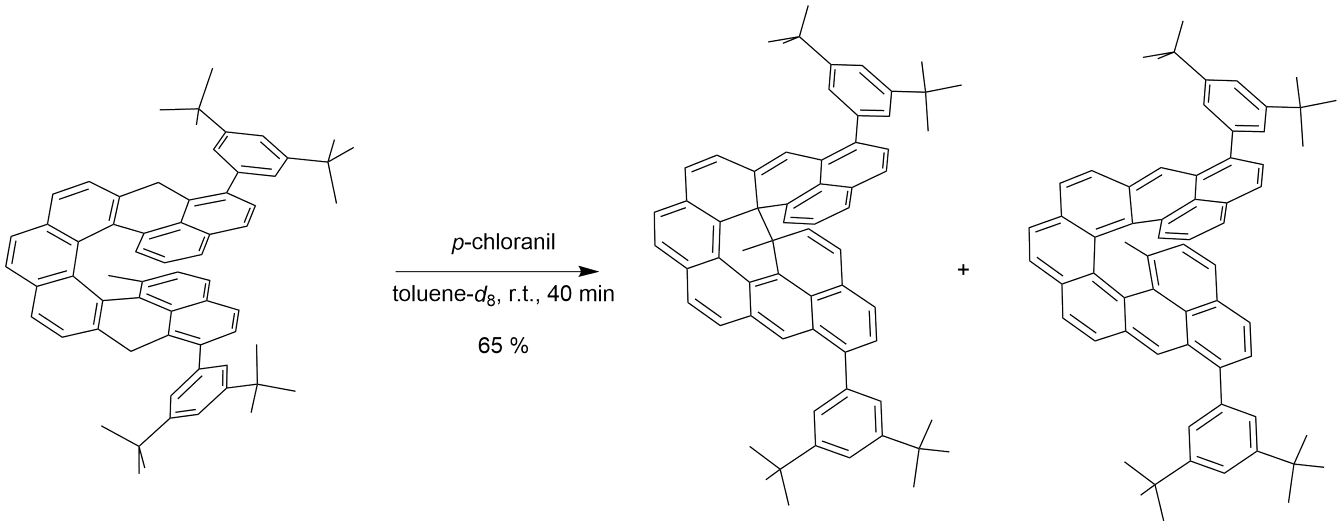

In a J Young-NMR tube under nitrogen atmosphere, 6,15-bis(3,5-di-*tert*-butylphenyl)-1-methyl-7,14-dihydrobenzo[*no*]benzo[8,9]anthra[1,2-*a*]tetraphene (2.87 mg, 3.40 µmol, 1.00 equiv) was dissolved in deuterated toluene (0.35 mL). A solution of *p*-chloranil (2.0 M in toluene-d_8, 0.10 mL, 4.1 µmol, 1.2 equiv) was added. The reaction progress was monitored by NMR spectroscopy at room temperature, during which the solution gradually changed from yellow to red. After 40 min, no starting material was detectable. The mixture was then filtered over silica gel using cyclohexane/CH_2Cl_2 (4:1) as an eluent, with the filtration carried out in a glove box under nitrogen atmosphere. The product was obtained as a red solid, and as a mixture of *c*-**MNC** and **MNC**.

Yield: 1.87 mg (2.22 mmol, 65 %)

^{1}H NMR (*c*-**MNC**, 298 K, 400 MHz, C_6D_6): *δ* 8.53 (s, 1H), 7.95–7.89 (m, 2H), 7.76–7.47 (m, 1H), 7.68 (d, $^3J_{HH}$ = 8.4 Hz, 1H), 7.66–7.64 (m, 1H), 7.55 (d, $^3J_{HH}$ = 8.4 Hz, 1H), 7.52 (d, $^3J_{HH}$ = 8.1 Hz, 1H), 7.50–7.47 (m, 2H), 7.45–7.43 (m, 2H), 7.35 (s, 1H), 7.26 (d, $^3J_{HH}$ = 7.8 Hz, 1H), 7.20 (d, $^3J_{HH}$ = 8.1 Hz, 1H), 7.06 (d, $^3J_{HH}$ = 10 Hz, 1H), 7.05 (d, $^3J_{HH}$ = 7.2 Hz, 1H), 6.99–6.95 (m, 1H), 6.50 (d, $^3J_{HH}$ = 7.2 Hz, 1H), 6.45 (d, $^3J_{HH}$ = 9.7 Hz, 1H), 6.38 (dd, $^3J_{HH}$ = 7.8 Hz, 1H), 6.23 (d, $^3J_{HH}$ = 9.7 Hz, 1H), 6.16 (d, $^3J_{HH}$ = 10 Hz, 1H), 1.65 (s, 3H), 1.42–1.39 (m, 36H) ppm.

^{13}C{^{1}H} NMR (*c*-**MNC**, 298 K, 151 MHz, C_6D_6): *δ* 147.35, 147.35, 141.2, 141.0, 140.8, 137.3, 136.6, 135.6, 135.0, 133.2, 133.0, 132.4, 132.2, 132.0, 131.7, 131.6, 130.8, 129.9, 129.6, 129.5, 128.43, 128.35, 127.9, 127.7, 127.6, 127.4, 127.34, 127.29, 126.70, 126.68, 126.3, 126.2, 125.8, 125.10, 125.09, 124.25, 124.16, 123.6, 122.4, 121.6, 121.2, 53.0, 52.3, 35.3, 35.1, 31.7, 30.2, 26.4 ppm.
One quaternary carbon signal could not be resolved and assigned due to signal overlap. See Figure S11 for details.

HRMS (APCI): *m/z* [*M* + H]$^+$ Calcd for $C_{65}H_{63}$ 843.4933; Found 843.4944.

3. Assignment of the NMR Resonances

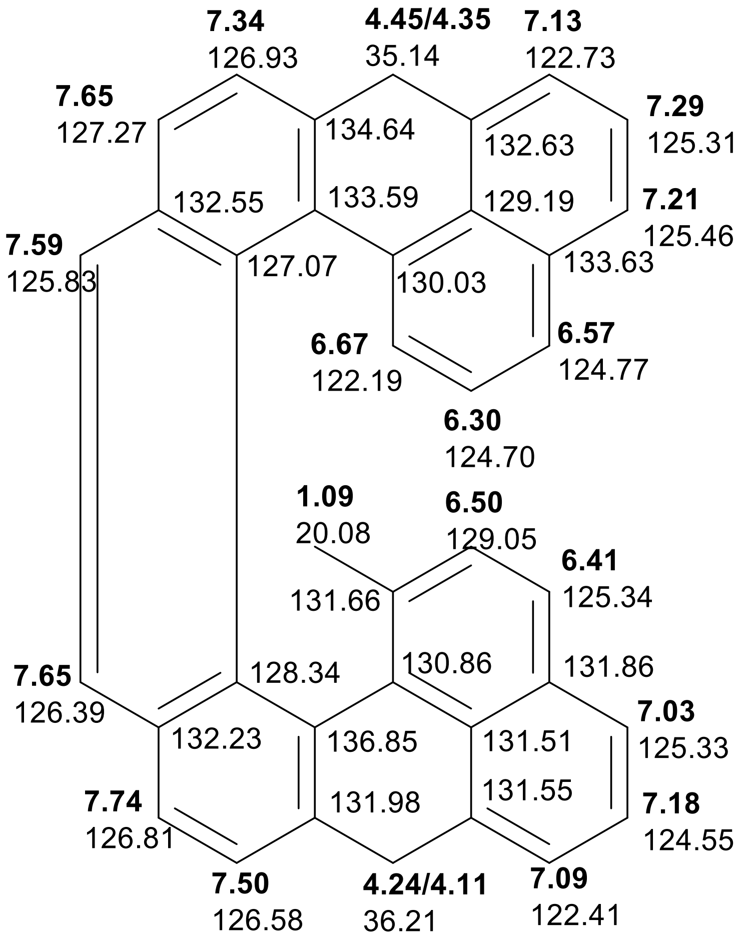

Figure S7. Assignment of ^{1}H (bold) and ^{13}C NMR resonances (in ppm) of 1-methyl-7,14-dihydro-benzo[*no*]benzo[8,9]anthra[1,2-*a*]tetraphene (**8**).

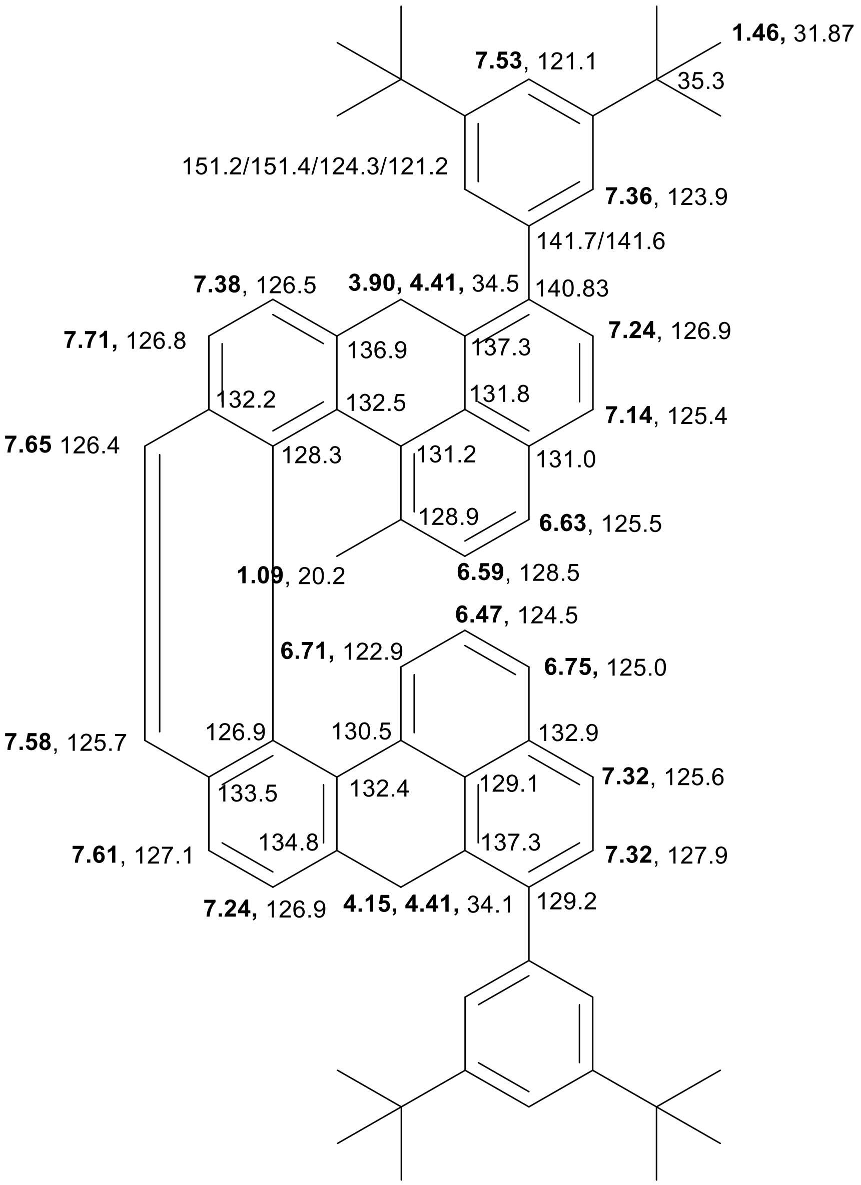

Figure S8. Assignment of ^{1}H (bold) and ^{13}C NMR resonances (in ppm) of 6,15-bis(3,5-di-*tert*-butylphenyl)-1-methyl-7,14-dihydrobenzo[*no*]benzo[8,9]anthra[1,2-*a*]tetraphene (**9**). The signals of the top and bottom di-*tert*-butylphenyl substituents overlap and are therefore not assigned for both substituents individually.

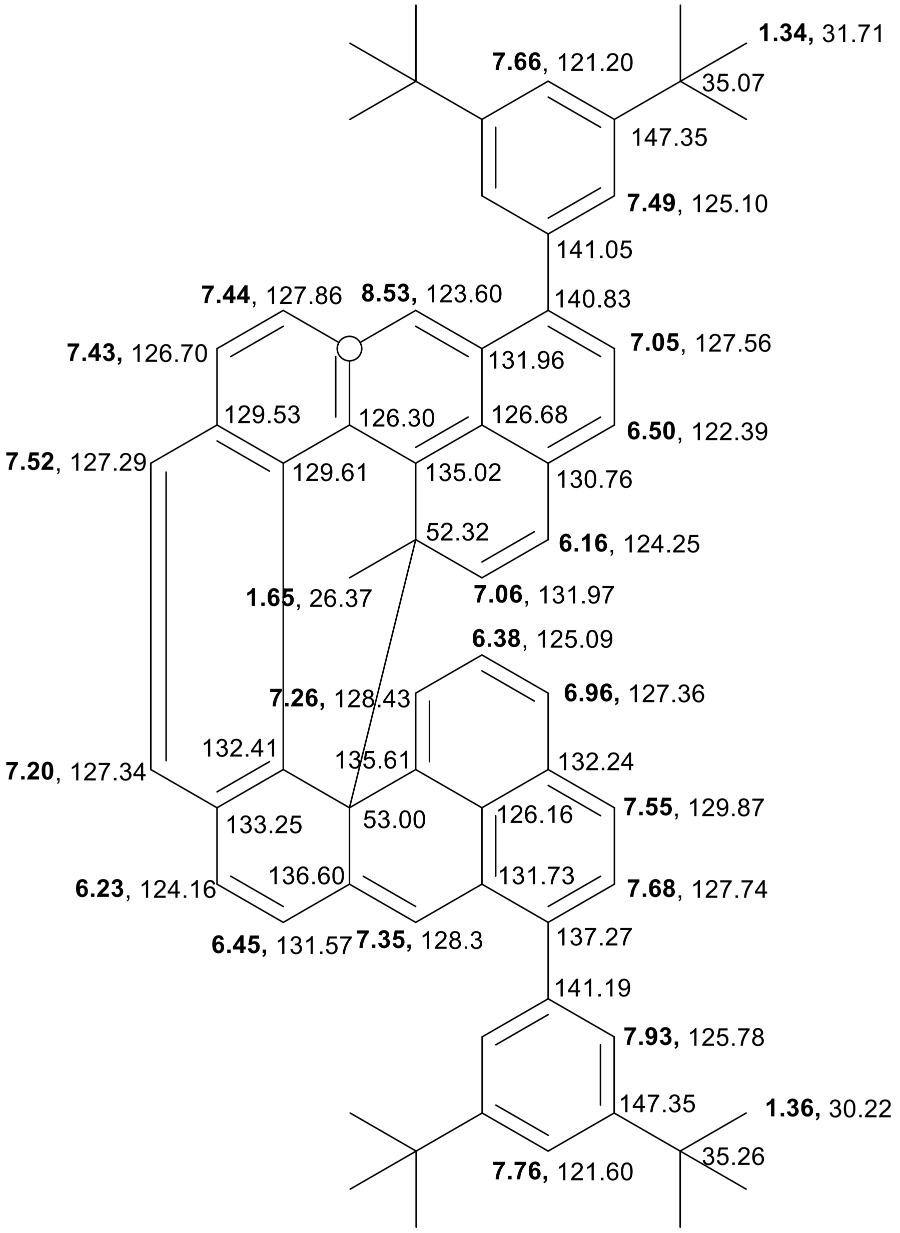

Figure S9. Assignment of ^{1}H (bold) and ^{13}C NMR resonances (in ppm) of 6,15-bis(3,5-di-*tert*-butylphenyl)-1-methylbenzo[*no*]benzo[8,9]anthra[1,2-*a*]tetraphene (*c*-**MNC**). The white circle marks one carbon atom that could not be resolved and assigned due to signal overlap.

4. Copies of the NMR Spectra

Methyl 3-(2-formyl-4-methylphenyl)propanoate (10)

^{1}H NMR / 400 MHz / $CDCl_3$

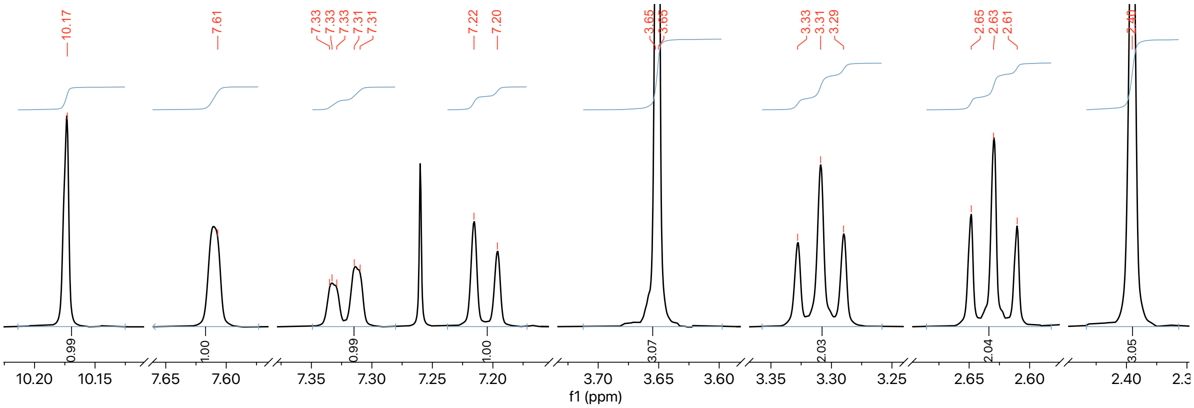

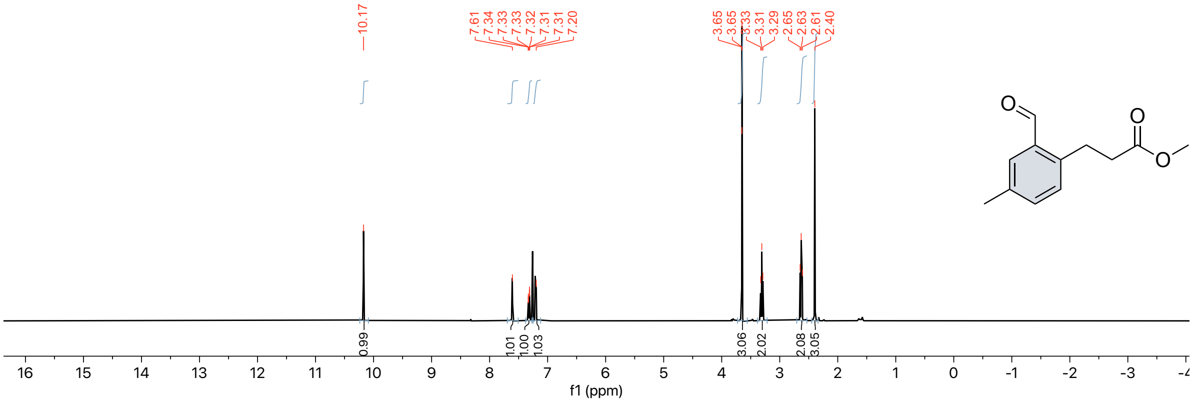

^{13}C NMR / 101 MHz / $CDCl_3$

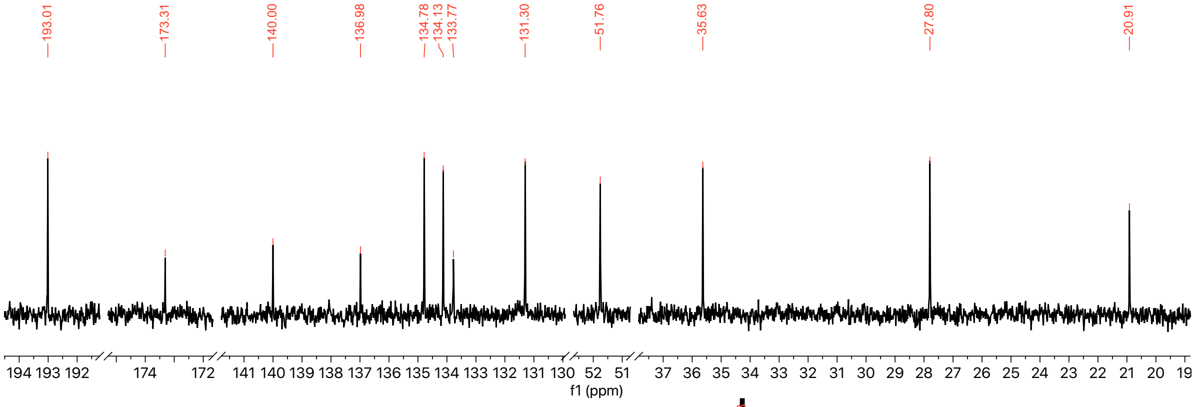

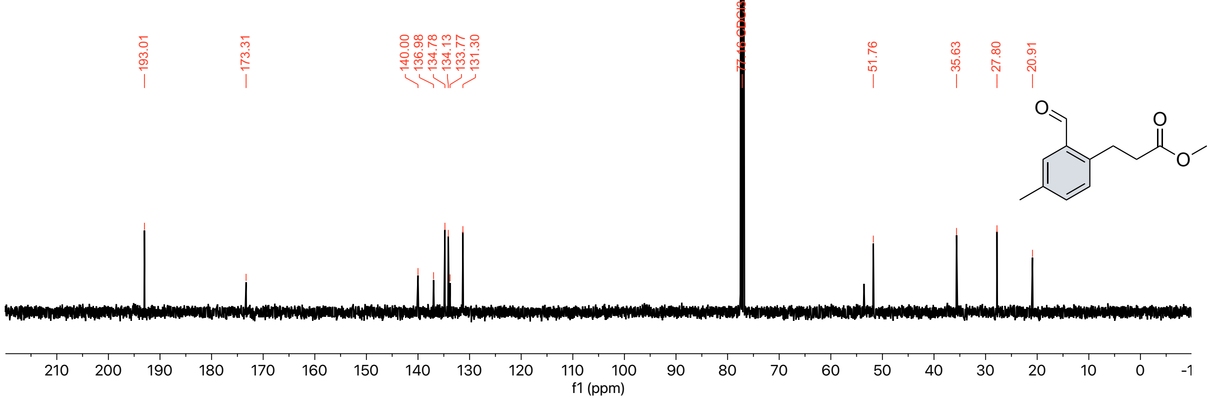

Methyl 3-(4-methyl-2-vinylphenyl)propanoate (4)

^{1}H NMR / 400 MHz / $CDCl_3$

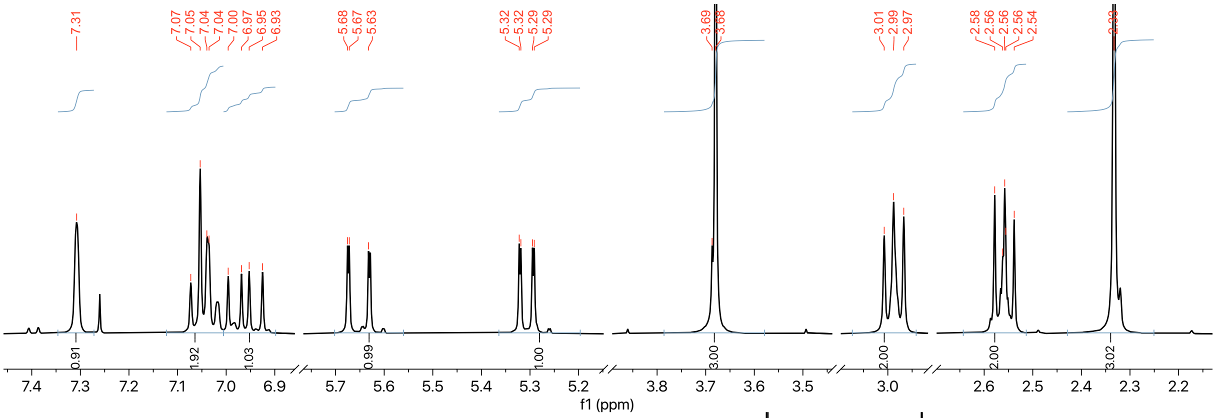

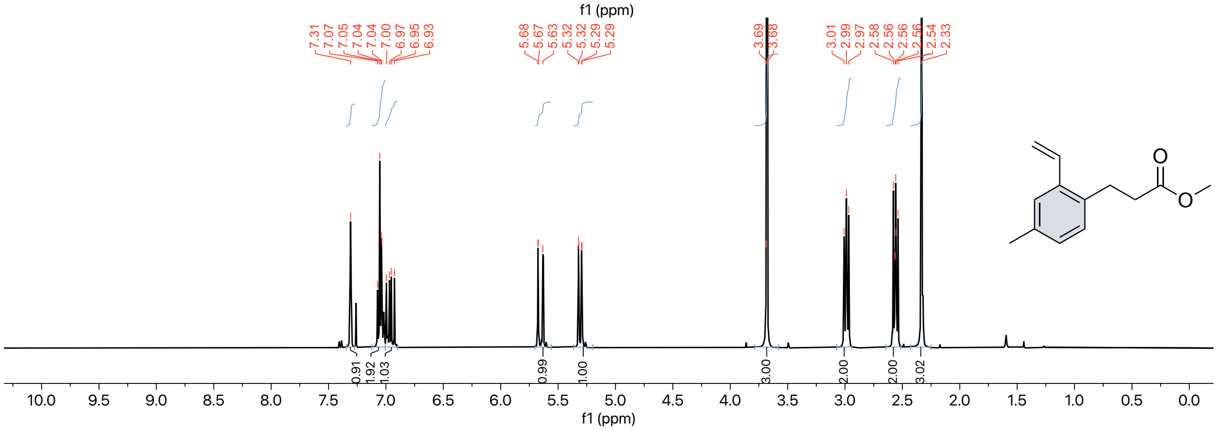

^{13}C NMR / 101 MHz / $CDCl_3$

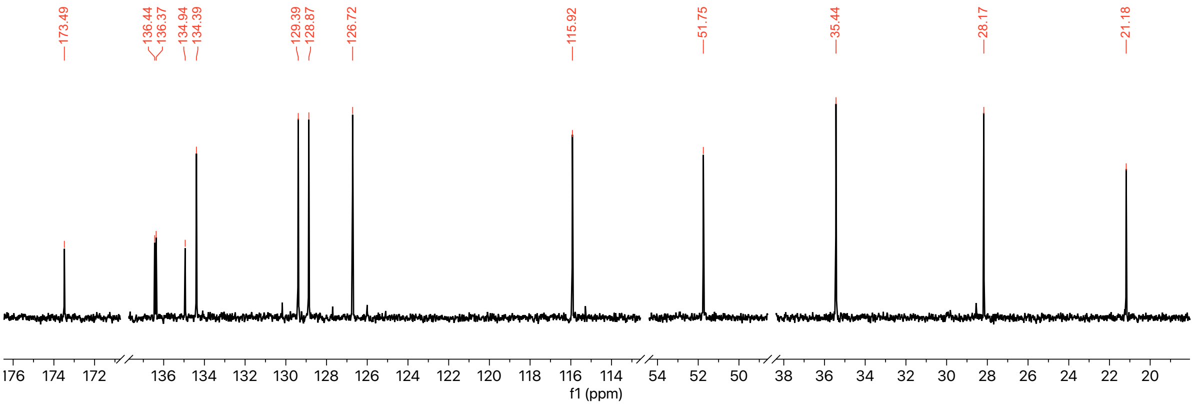

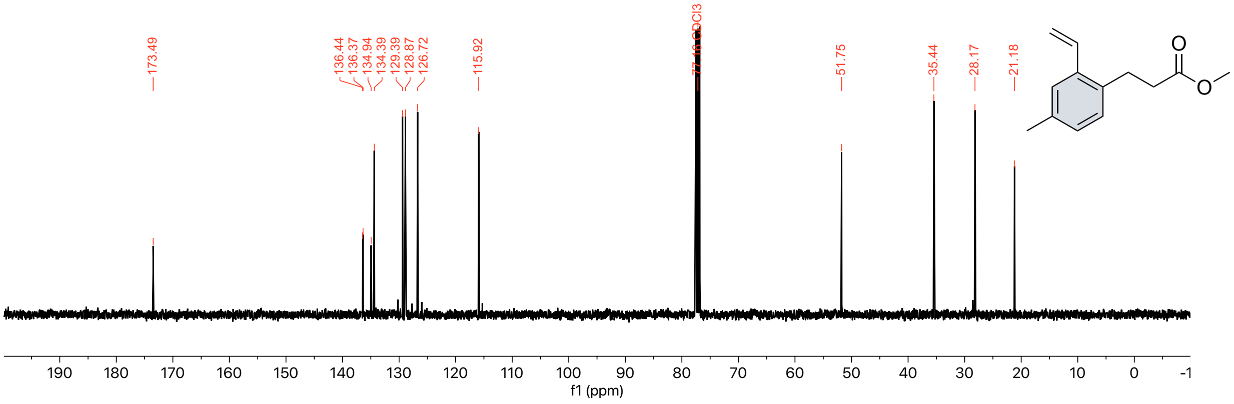

6-Bromophenanthrene-3-carbaldehyde (1)

^{1}H NMR / 400 MHz / $CDCl_3$

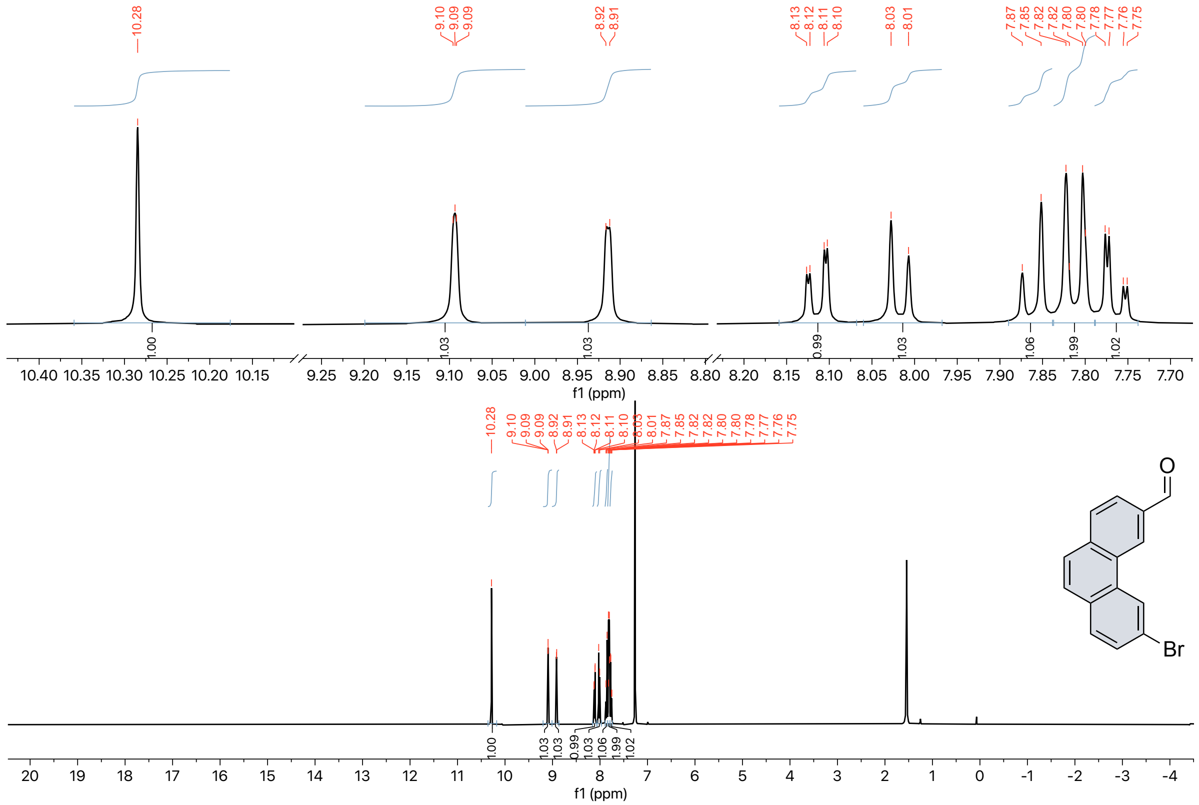

^{13}C NMR / 101 MHz / $CDCl_3$

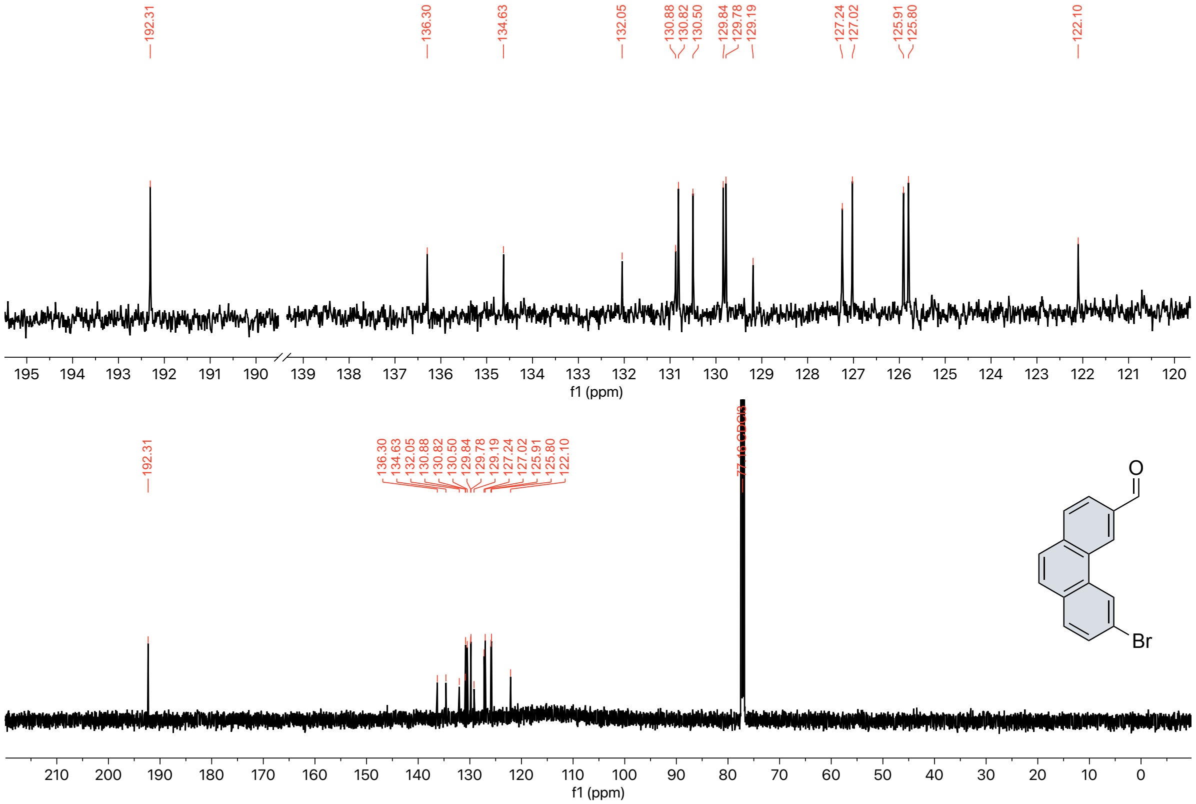

Methyl (*E*)-3-(2-(2-(6-bromophenanthren-3-yl)vinyl)phenyl) propanoate (3)

^{1}H NMR / 400 MHz / $CDCl_3$

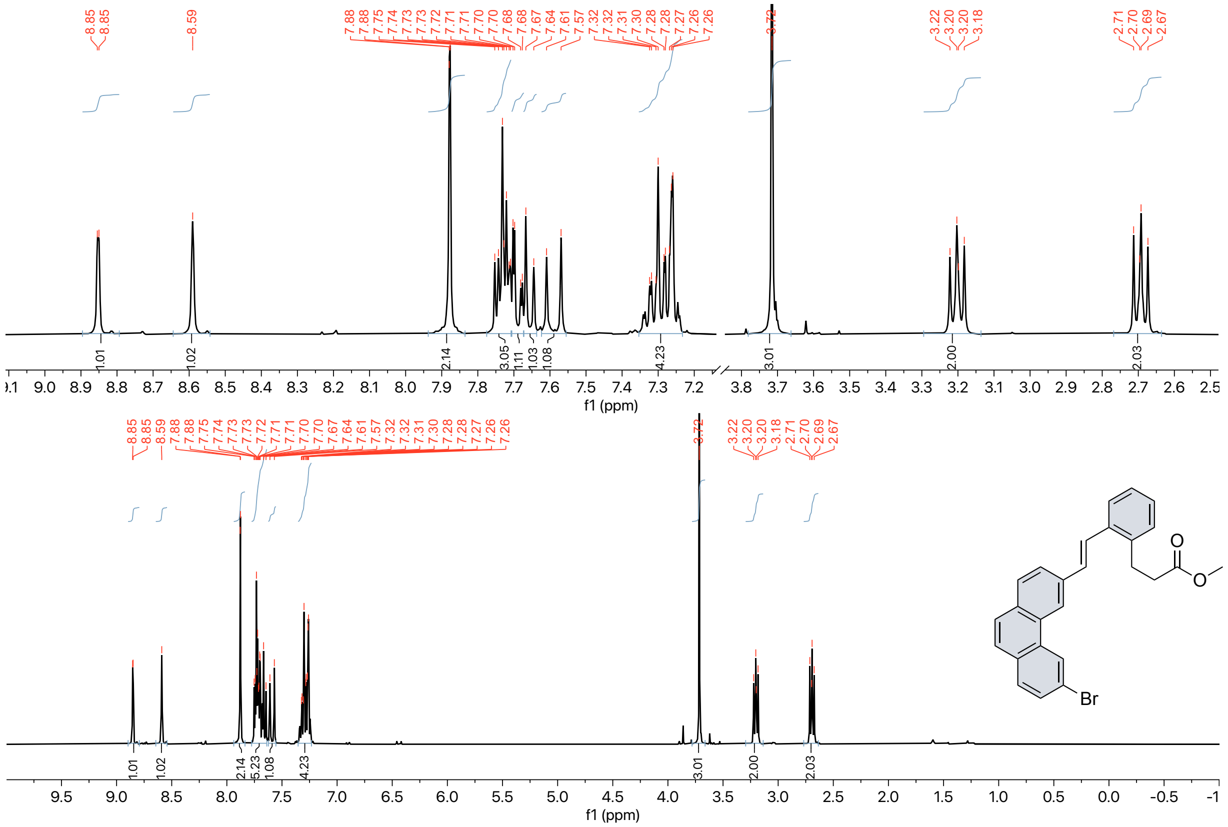

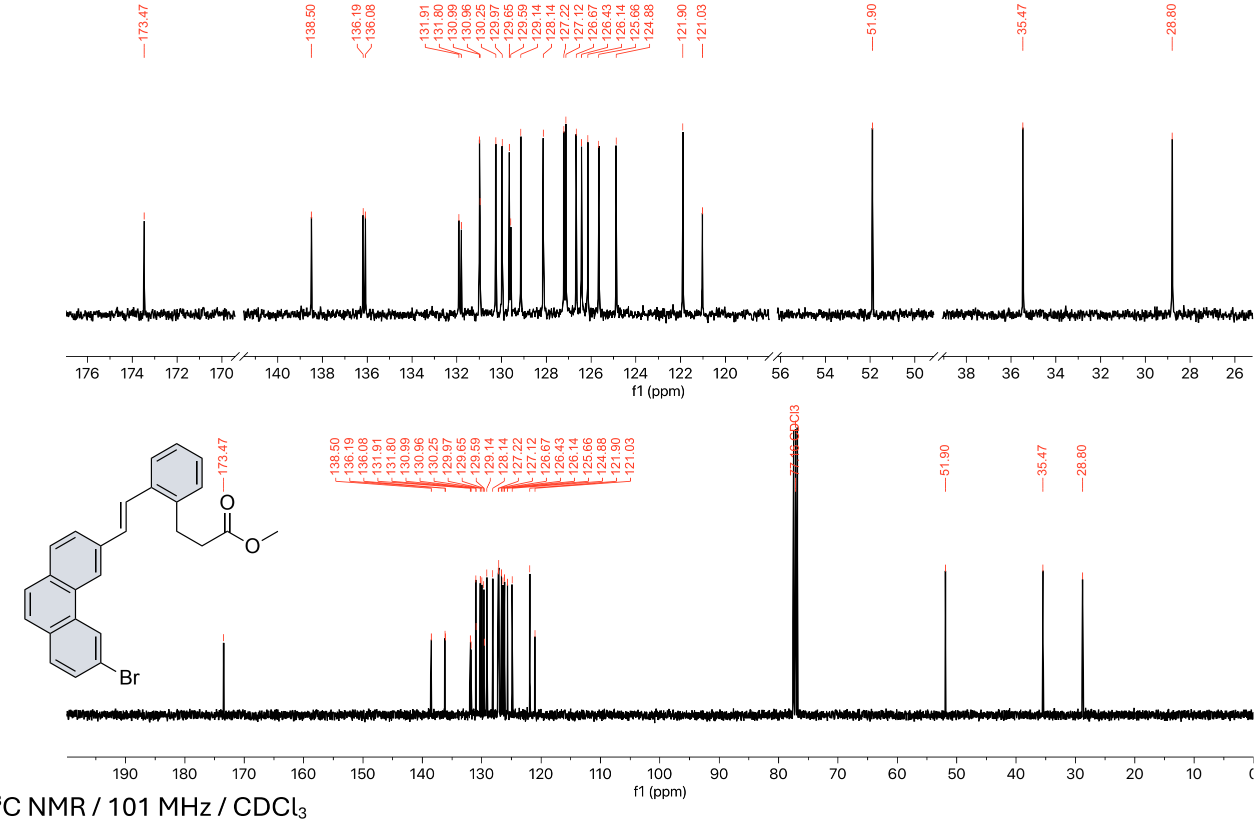

^{13}C NMR / 101 MHz / $CDCl_3$

(*E*)- and (*Z*)-3-(2-(2-(6-bromophenanthren-3-yl)vinyl)phenyl) propanoate (3)

^{1}H NMR / 400 MHz / $CDCl_3$

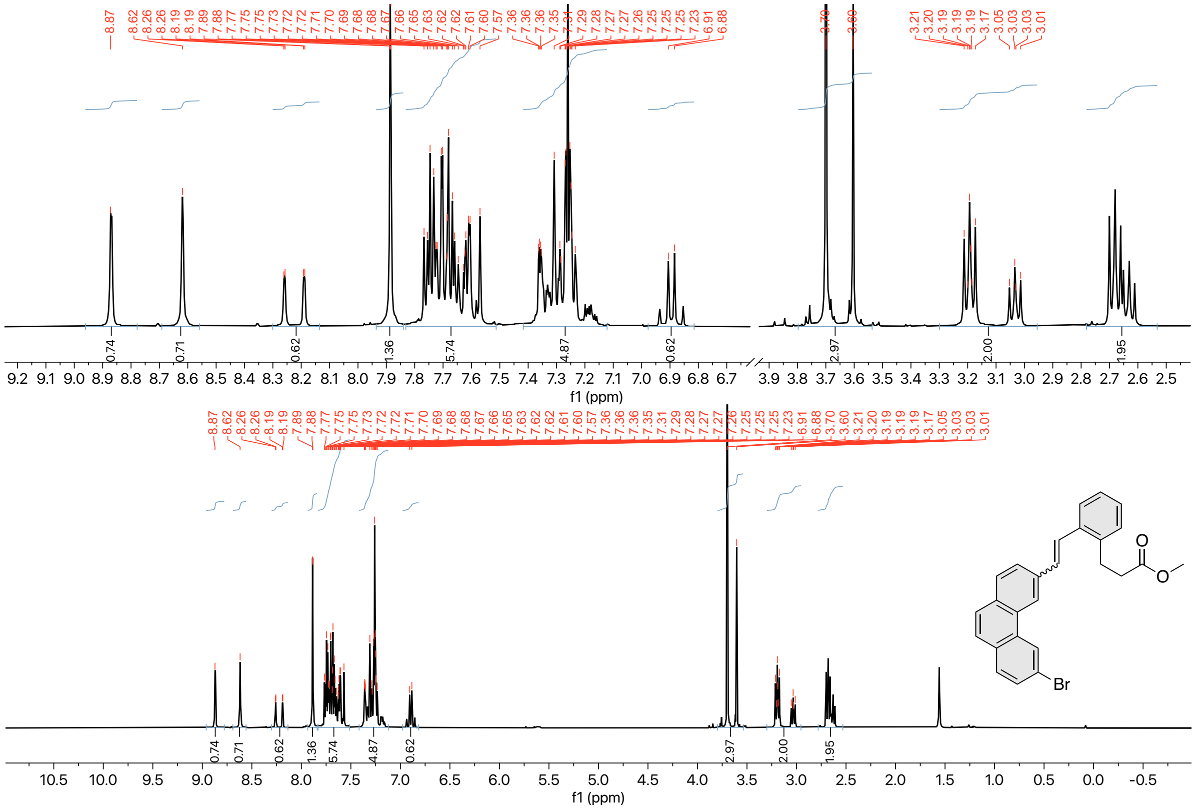

Dimethyl 3,3'-(((1*E*,1'*E*)-phenanthrene-3,6-diylbis(ethene-2,1-diyl))bis(2,1-phenylene))dipropionate methyl (5)

^{1}H NMR / 400 MHz / CDCl$_3$

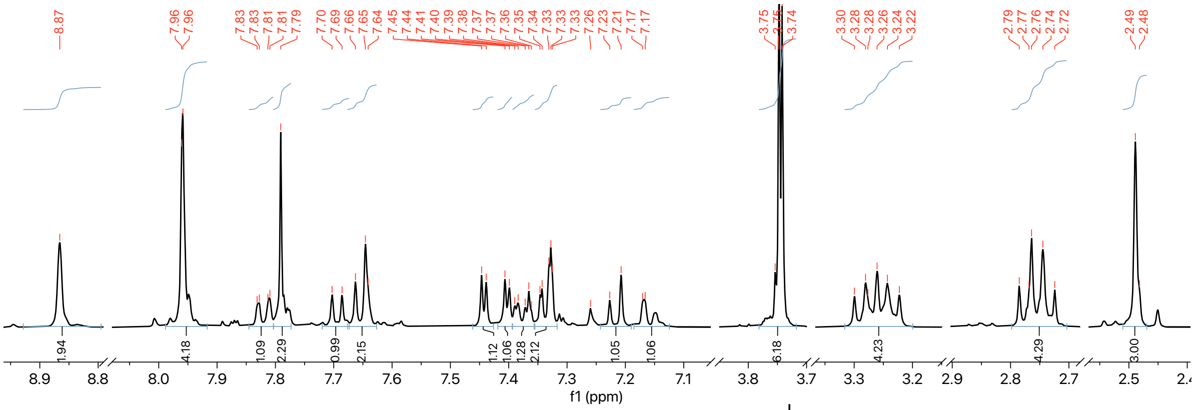

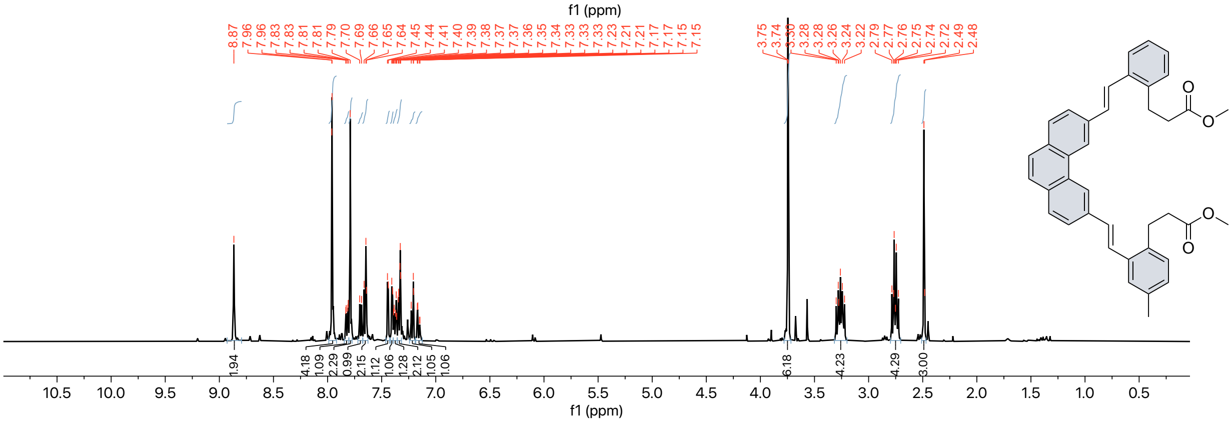

^{13}C NMR / 101 MHz / CDCl$_3$

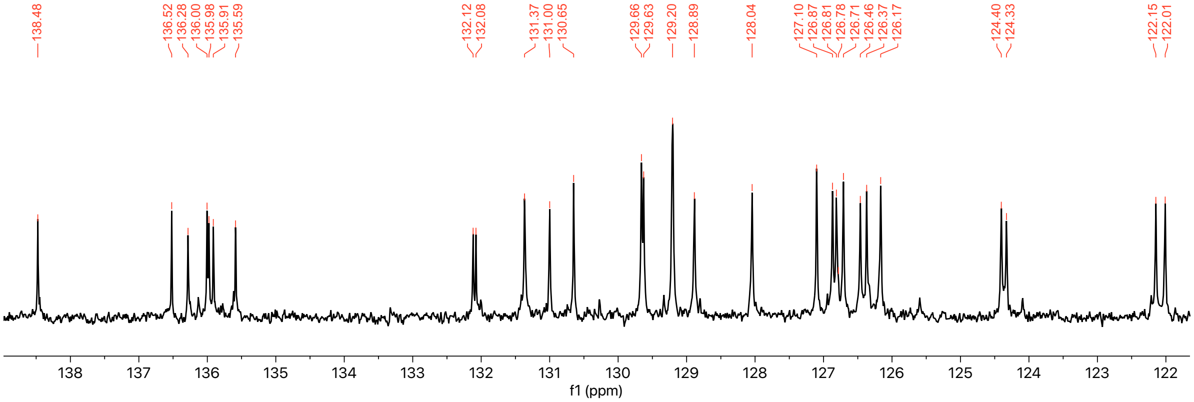

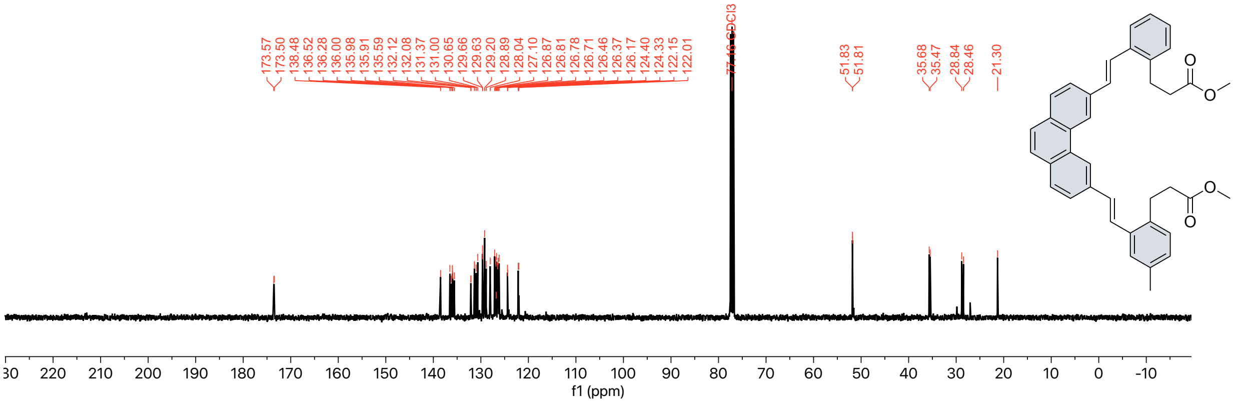

Dimethyl 3,3'-(1-methylbenzo[1,2-*c*:4,3-*c*']diphenanthrene-4,15-diyl)dipropionate (6)

^{1}H NMR / 400 MHz / $CDCl_3$

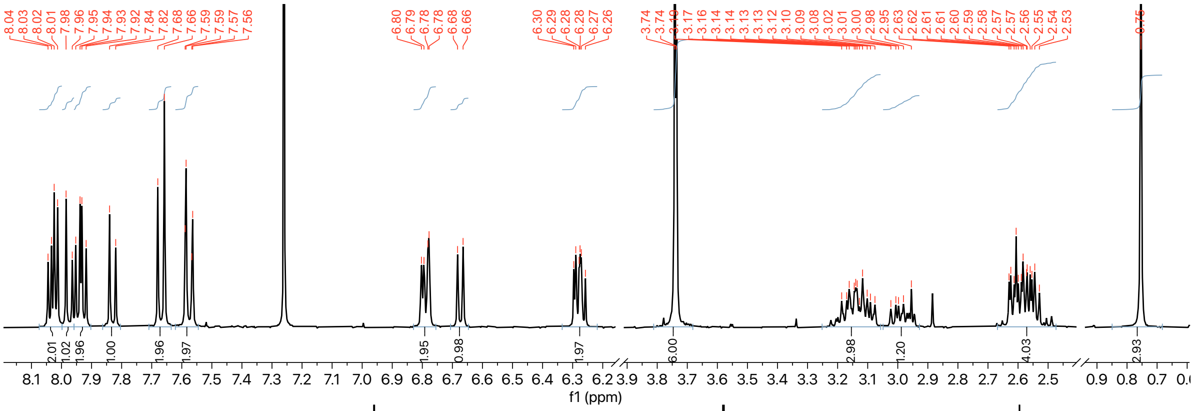

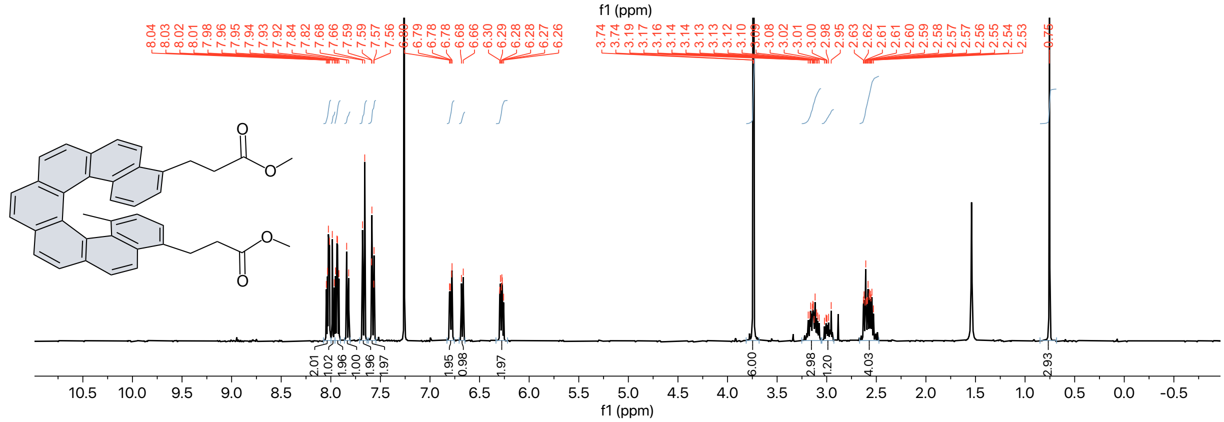

^{13}C NMR / 101 MHz / $CDCl_3$

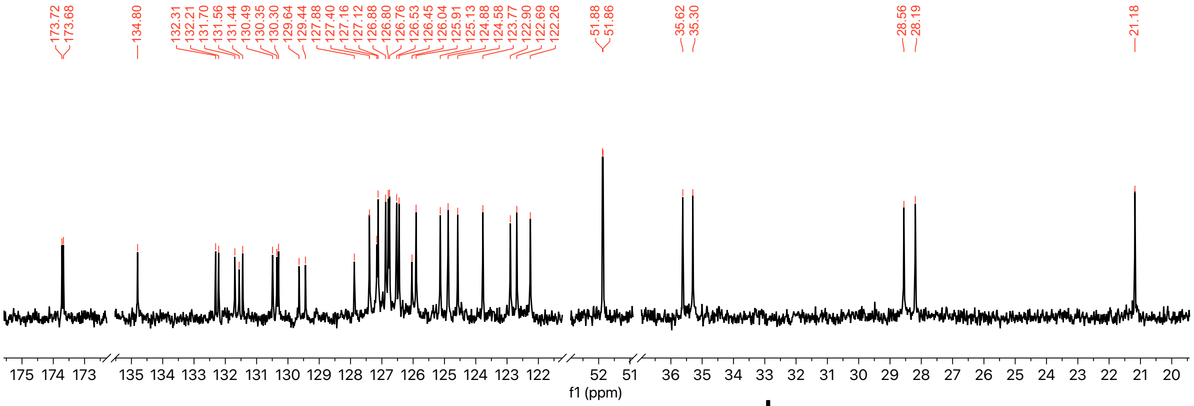

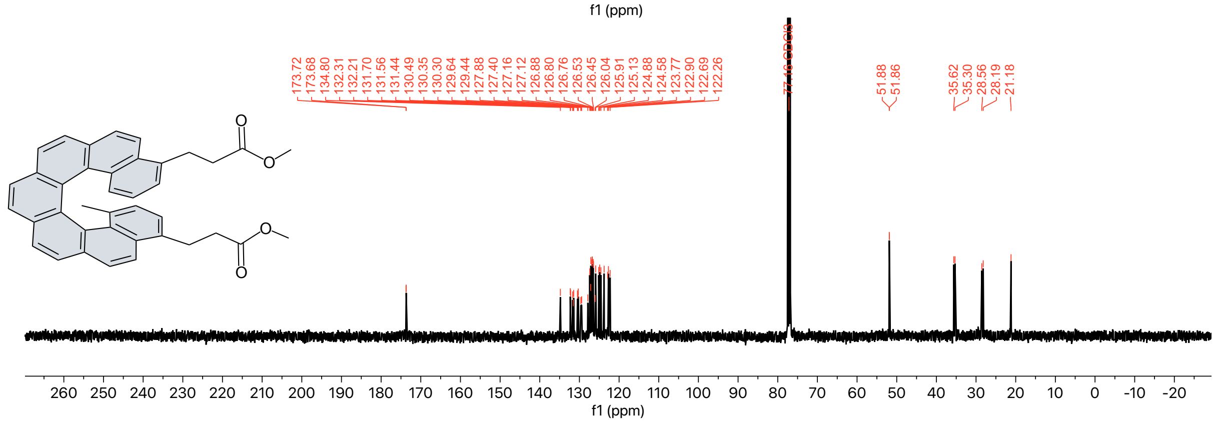

3,3'-(1-Methylbenzo[1,2-*c*:4,3-*c*']diphenanthrene-4,15-diyl)dipropionic acid (11)

^{1}H NMR / 400 MHz / acetone-d_6

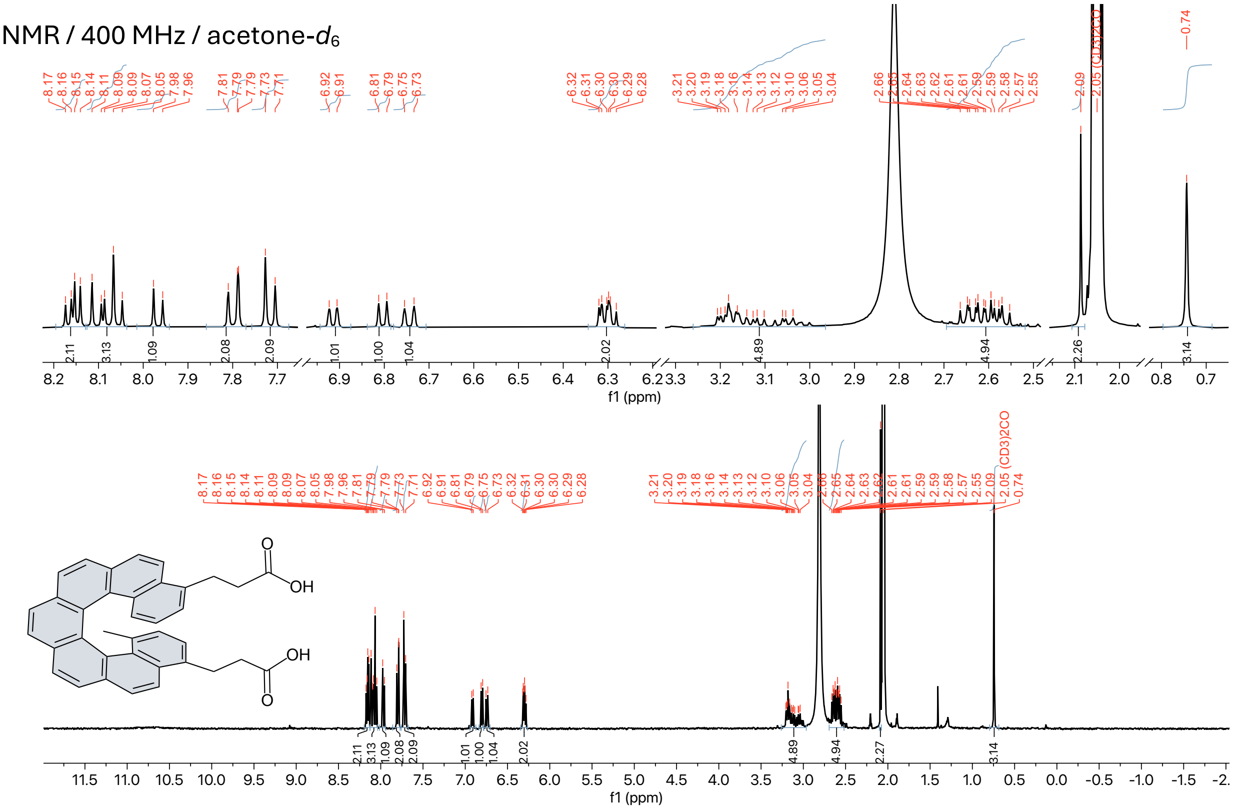

1-Methyl-4,5,16,17-tetrahydrobenzo[*no*]benzo[8,9]anthra[1,2-*a*]tetraphene-6,15-dione (7)

^{1}H NMR / 400 MHz / $CDCl_3$

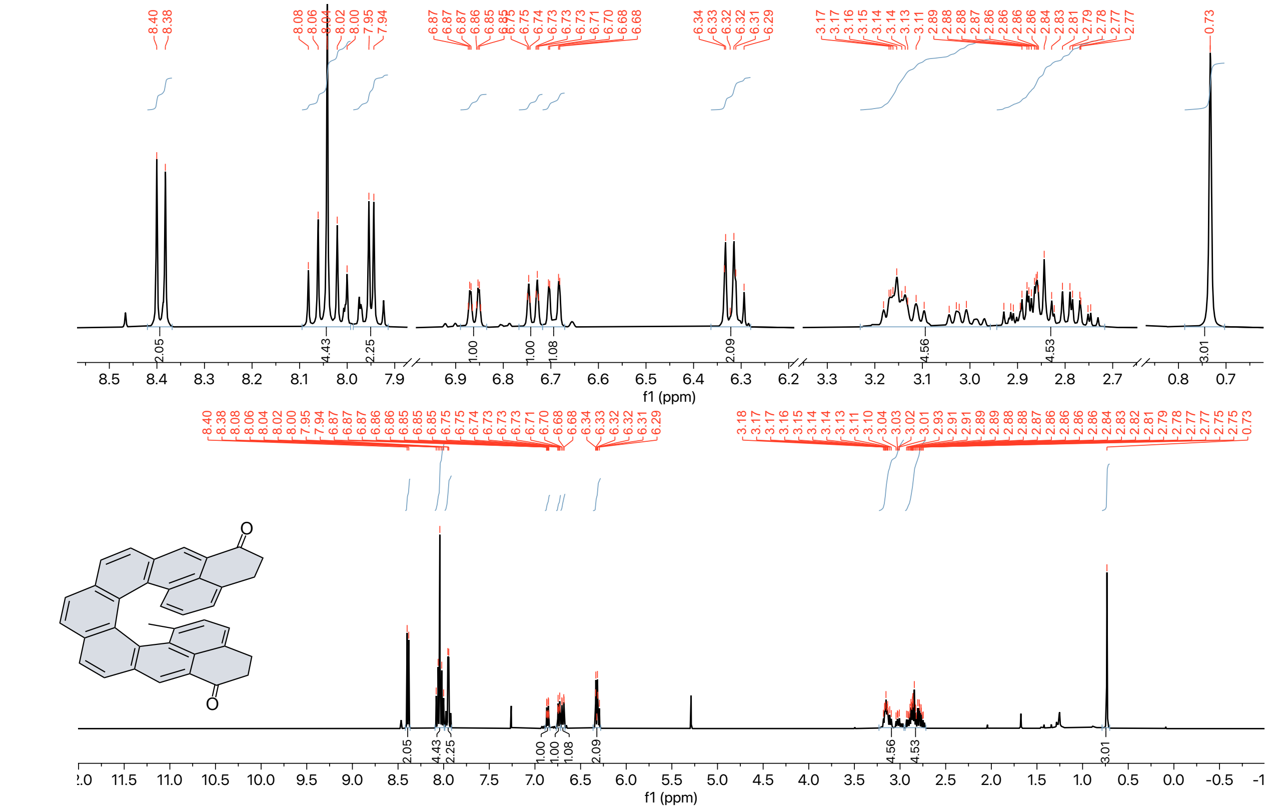

^{13}C NMR / 101 MHz / $CDCl_3$

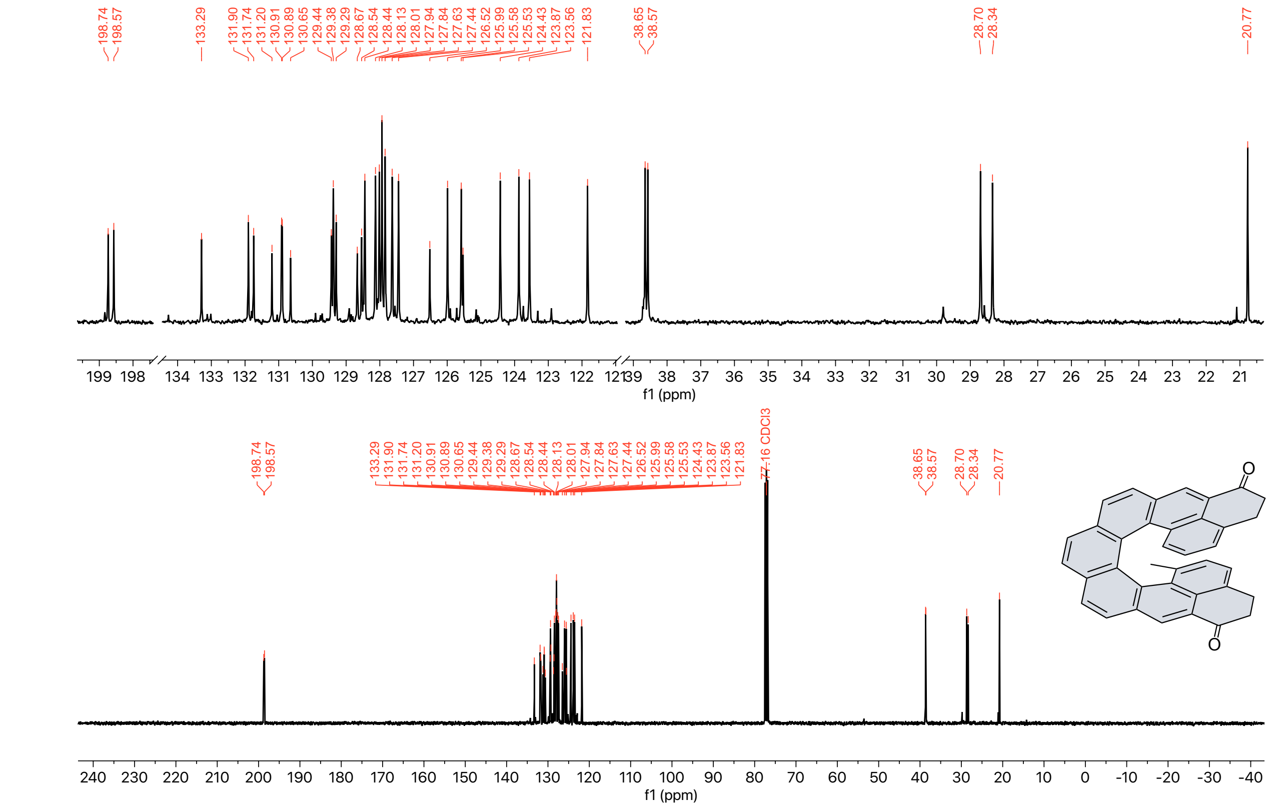

1-Methyl-7,14-dihydrobenzo[*no*]benzo[8,9]anthra[1,2-*a*]tetraphene (8)

^{1}H NMR / 500 MHz / CD_2Cl_2

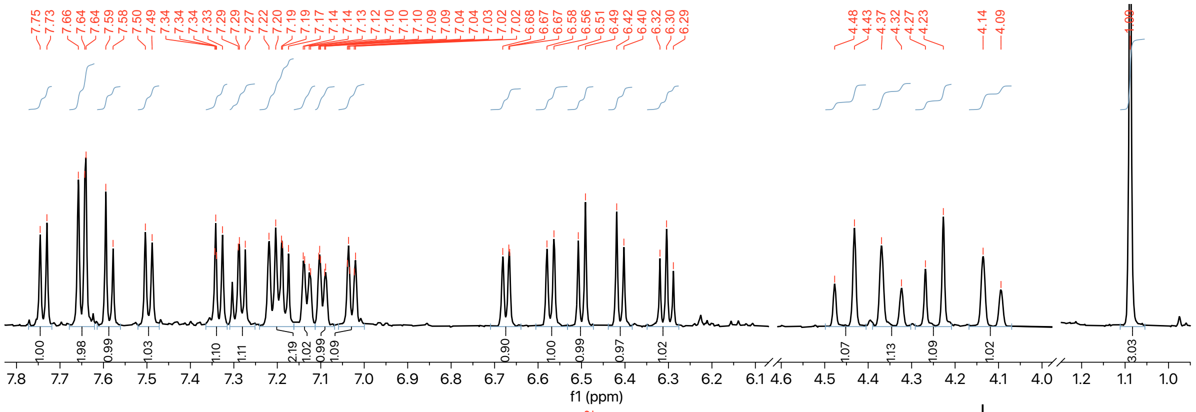

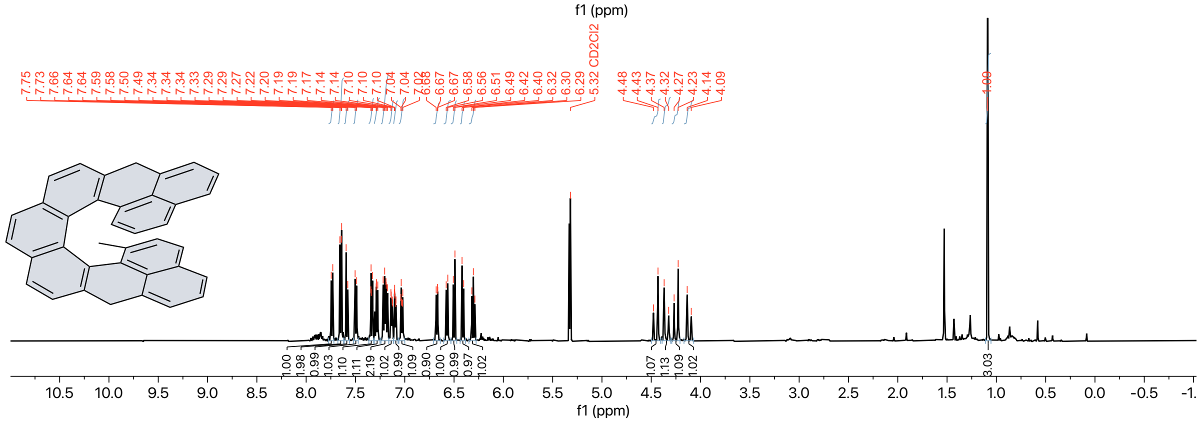

^{13}C NMR / 126 MHz / CD_2Cl_2

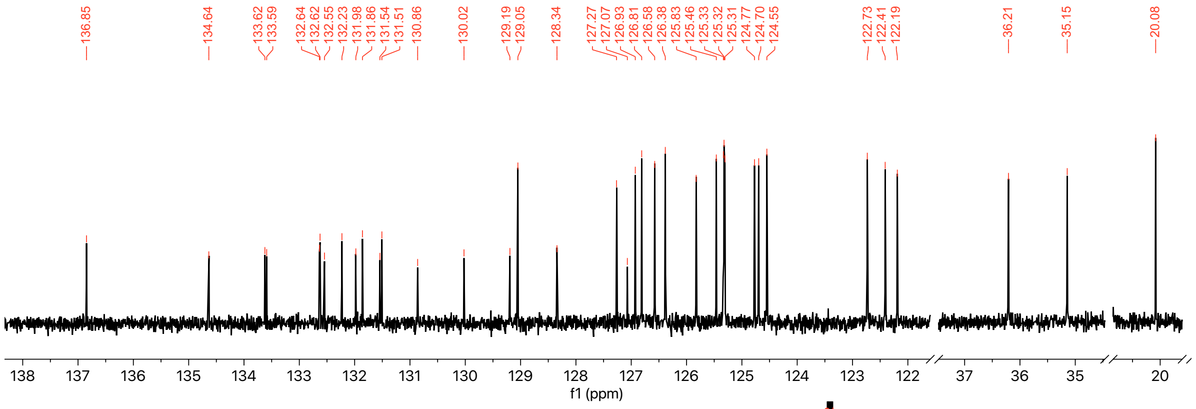

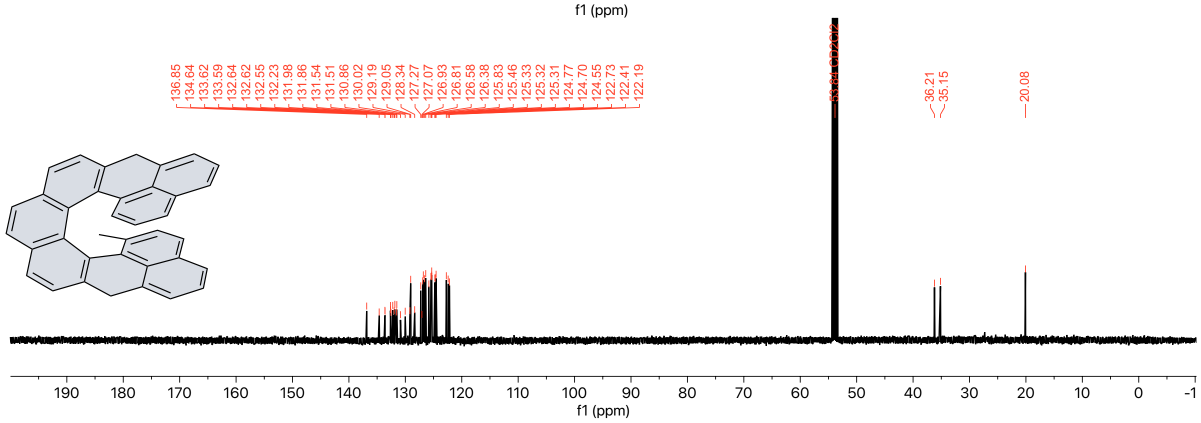

COSY NMR / 500 MHz / CD_2Cl_2

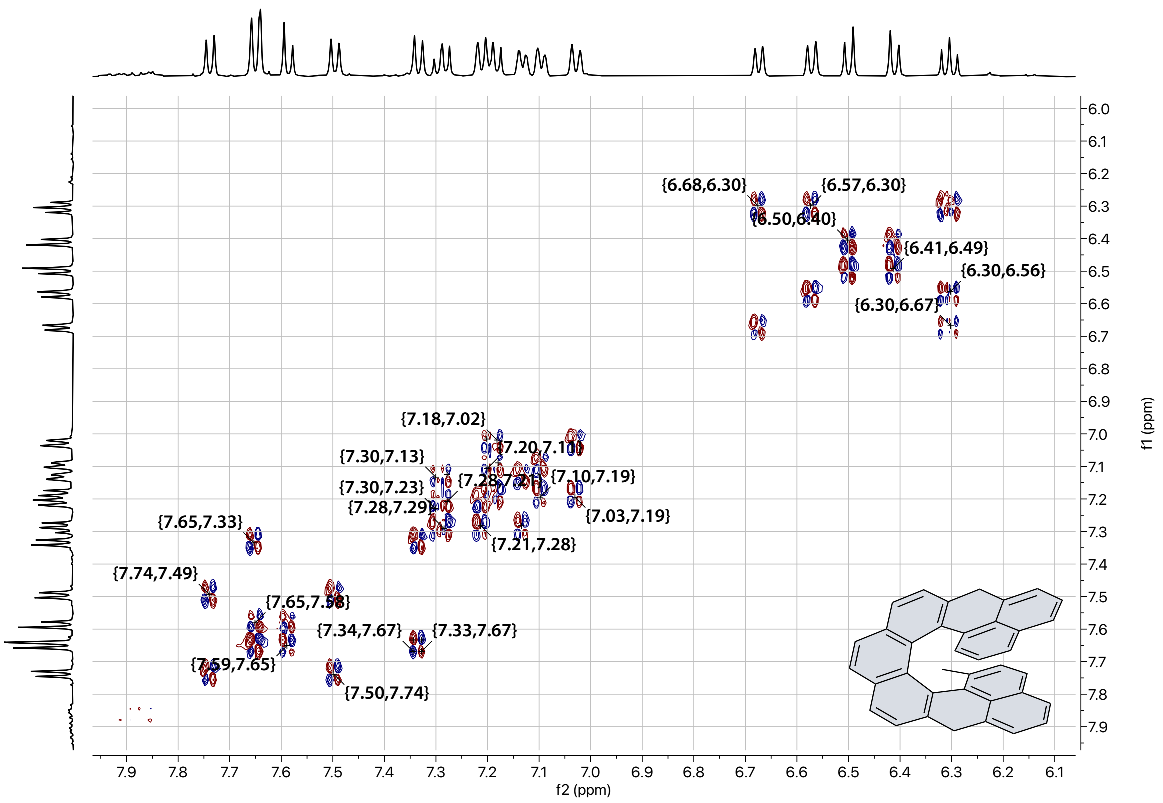

NOESY NMR / 500 MHz / CD_2Cl_2

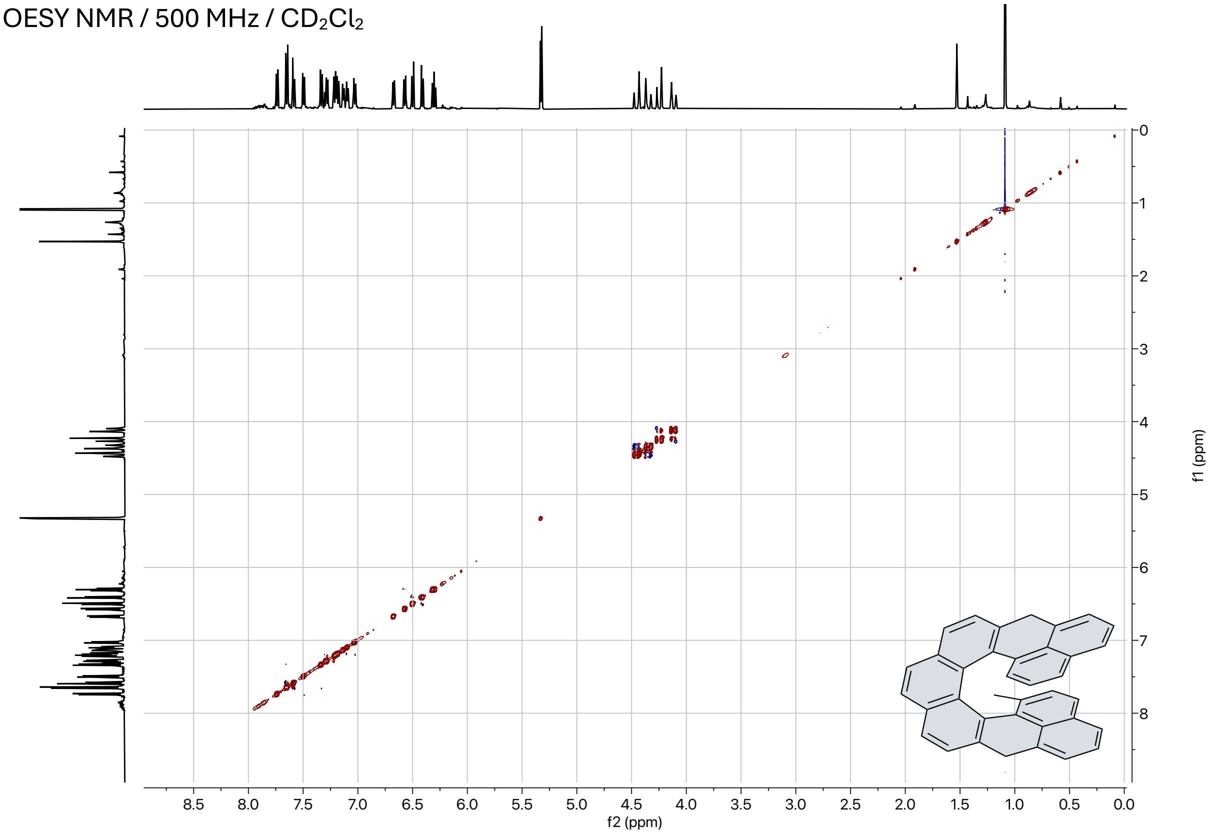

HMBC NMR / 500 MHz; 126 MHz / CD_2Cl_2

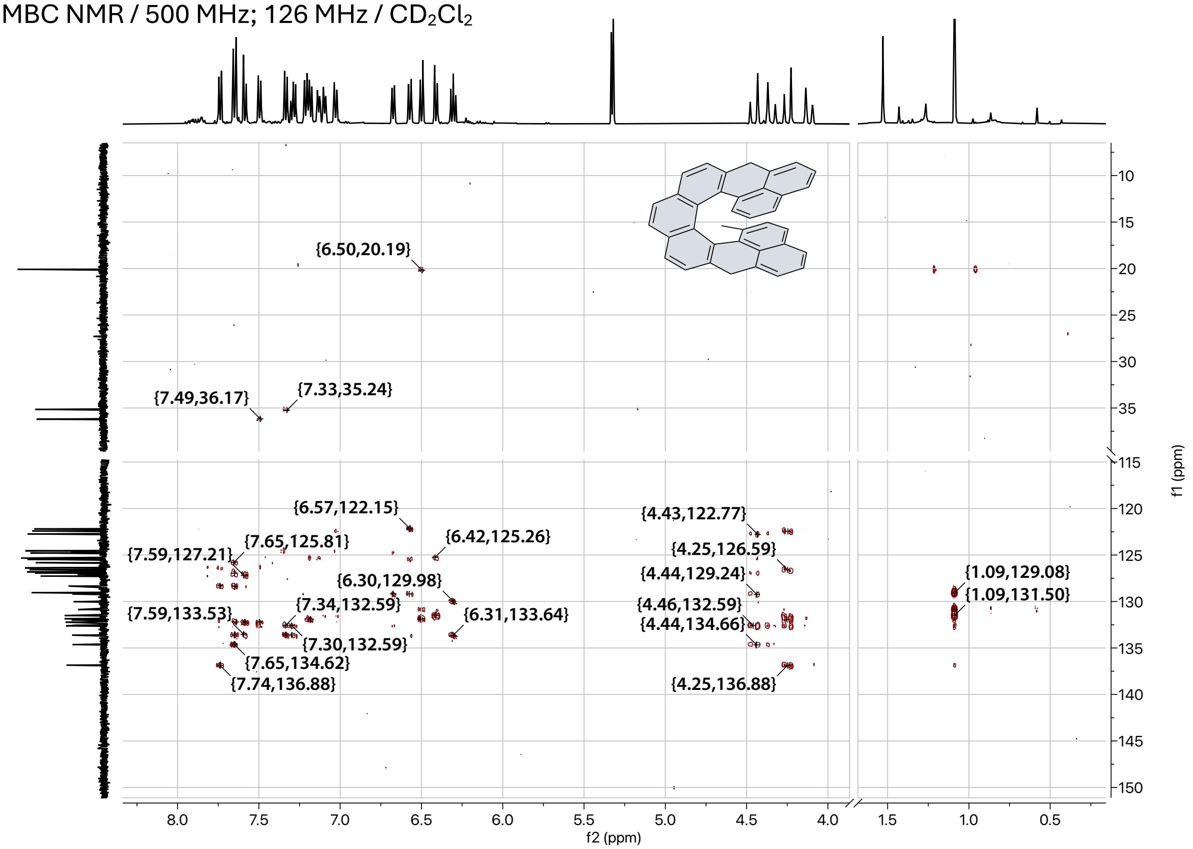

HSQC NMR / 500 MHz; 126 MHz / CD_2Cl_2

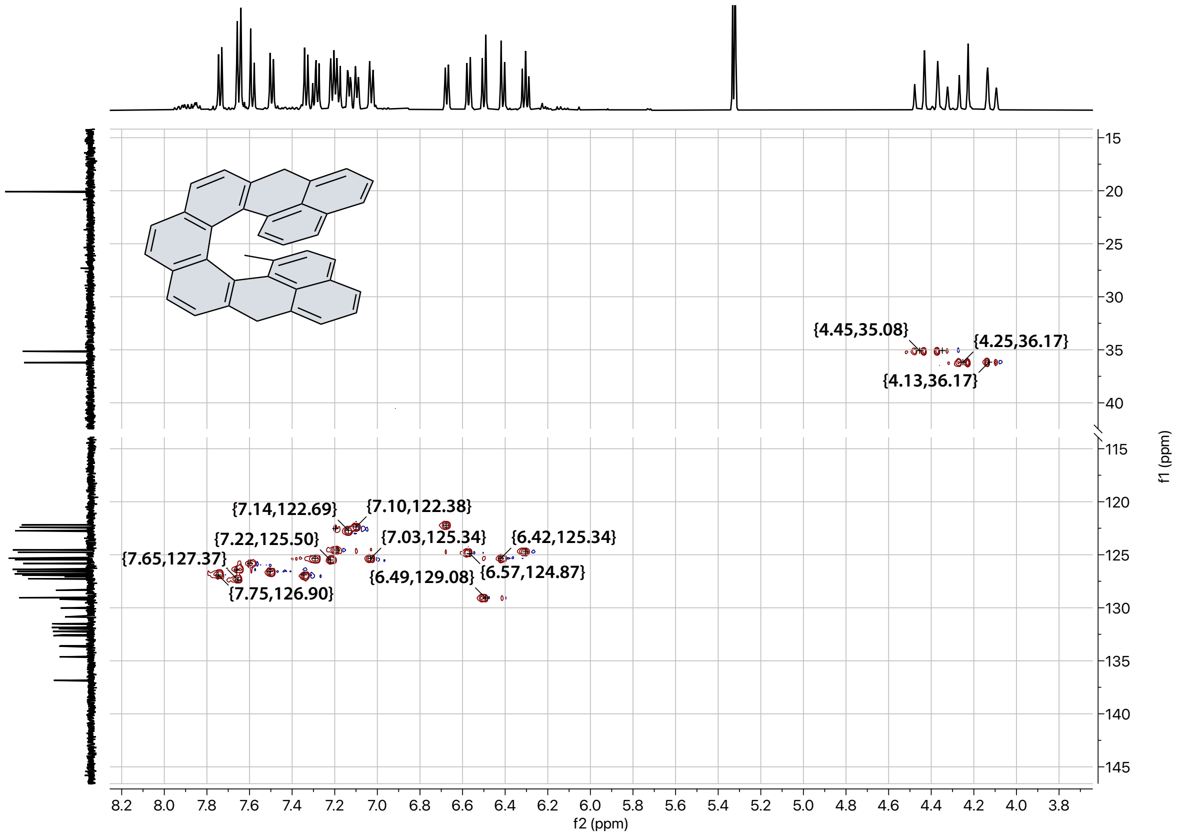

6,15-Bis(3,5-di-*tert*-butylphenyl)-1-methyl-7,14-dihydrobenzo[*no*]benzo[8,9]anthra[1,2-*a*]tetraphene (9)

^{1}H NMR / 500 MHz / CD_2Cl_2

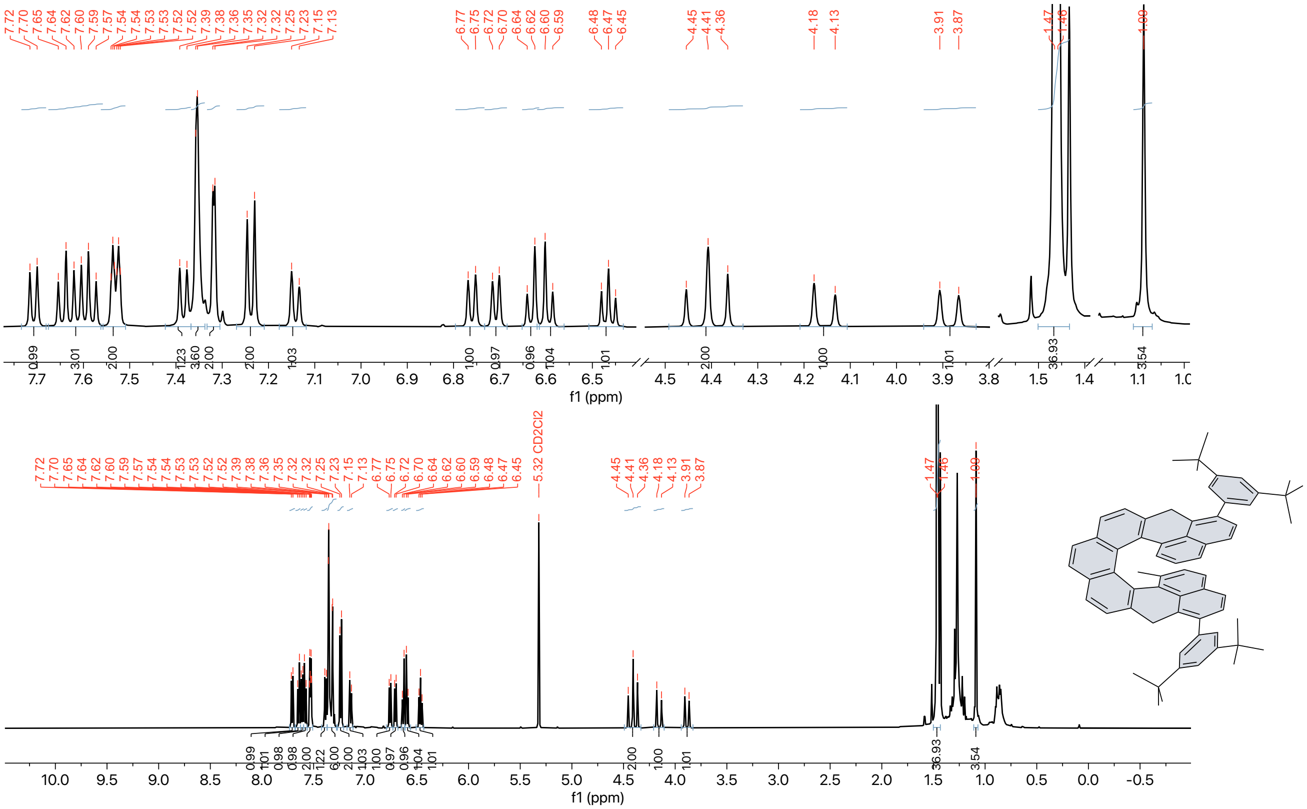

^{13}C NMR / 126 MHz / CD_2Cl_2

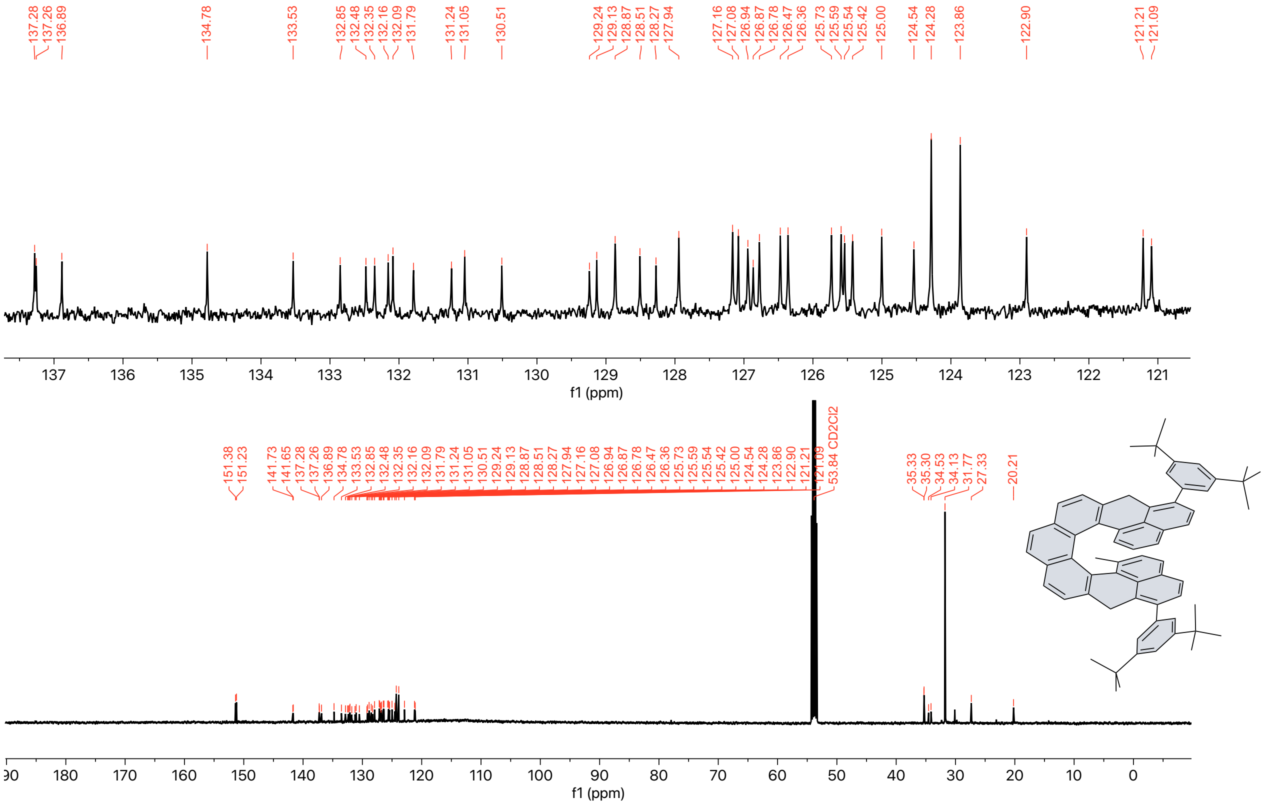

COSY NMR / 500 MHz / CD_2Cl_2

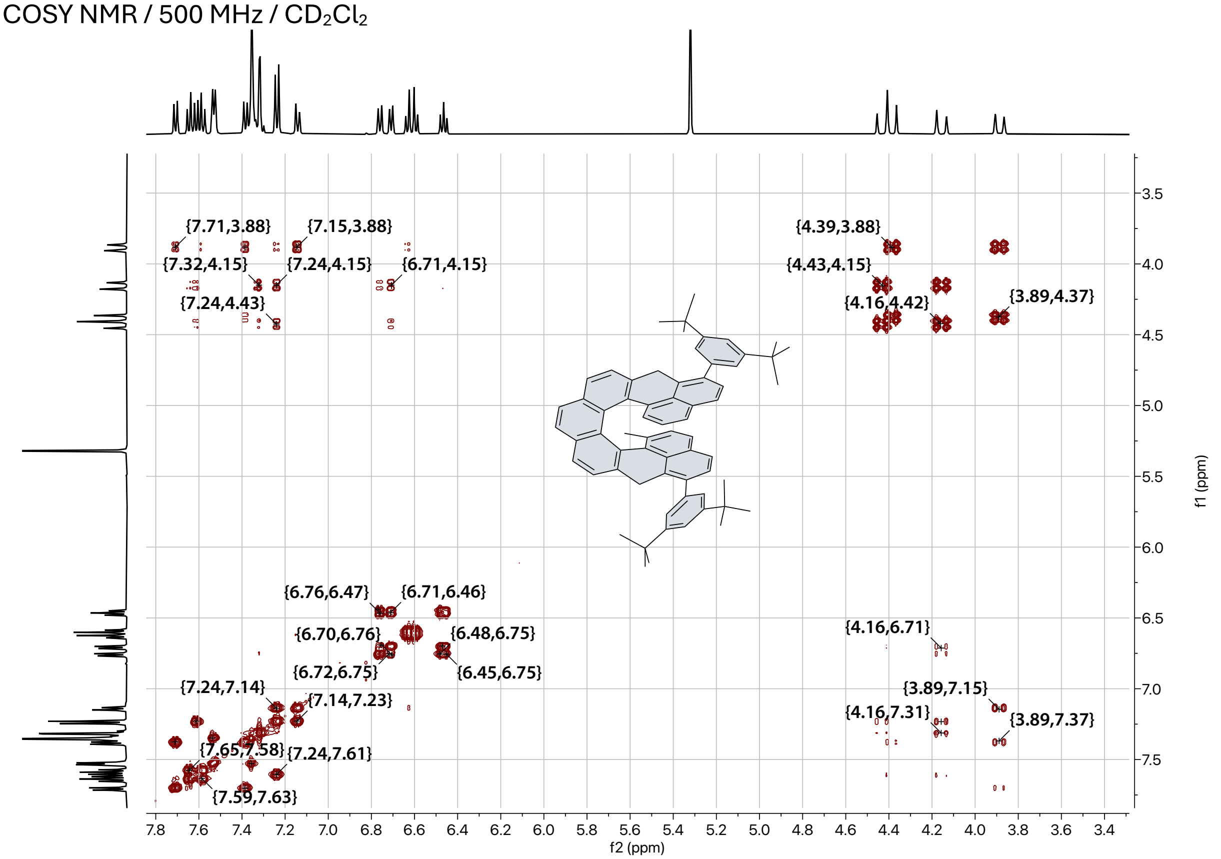

HMBC NMR / 500 MHz; 126 MHz / CD_2Cl_2

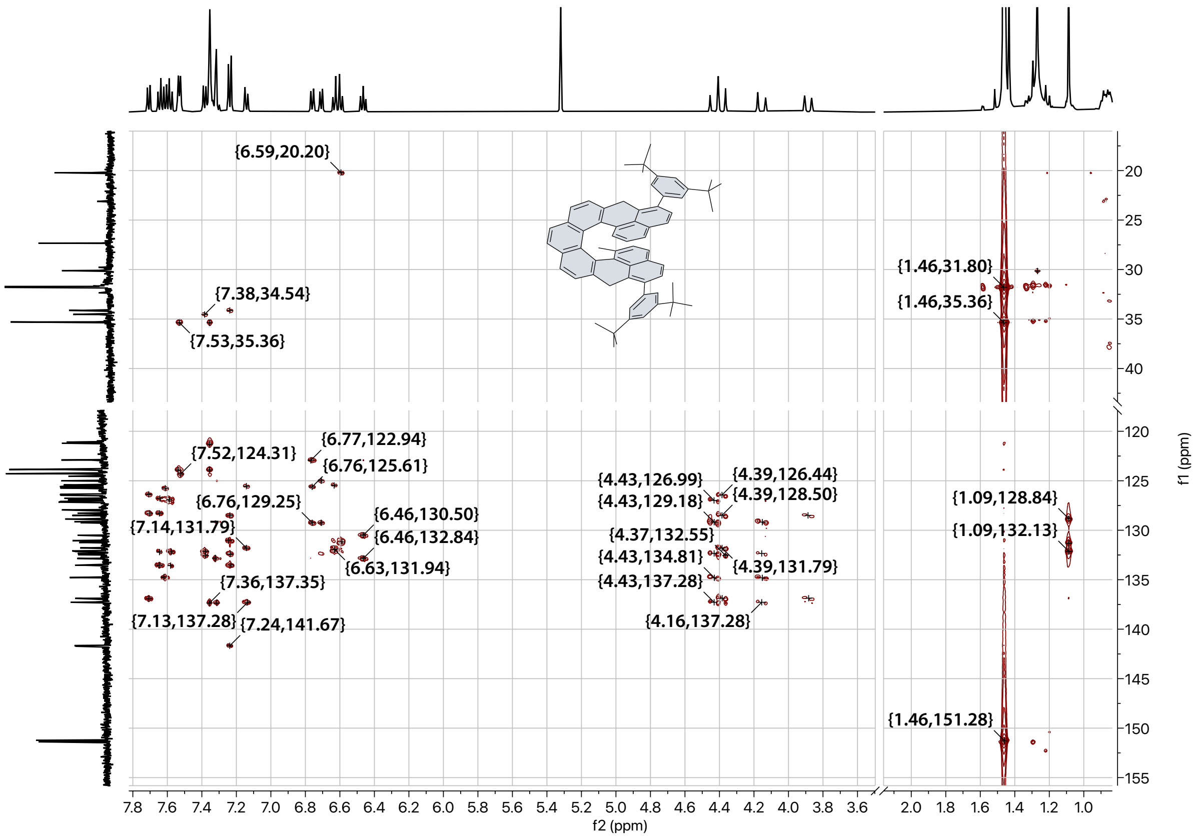

HSQC NMR / 500 MHz; 126 MHz / CD_2Cl_2

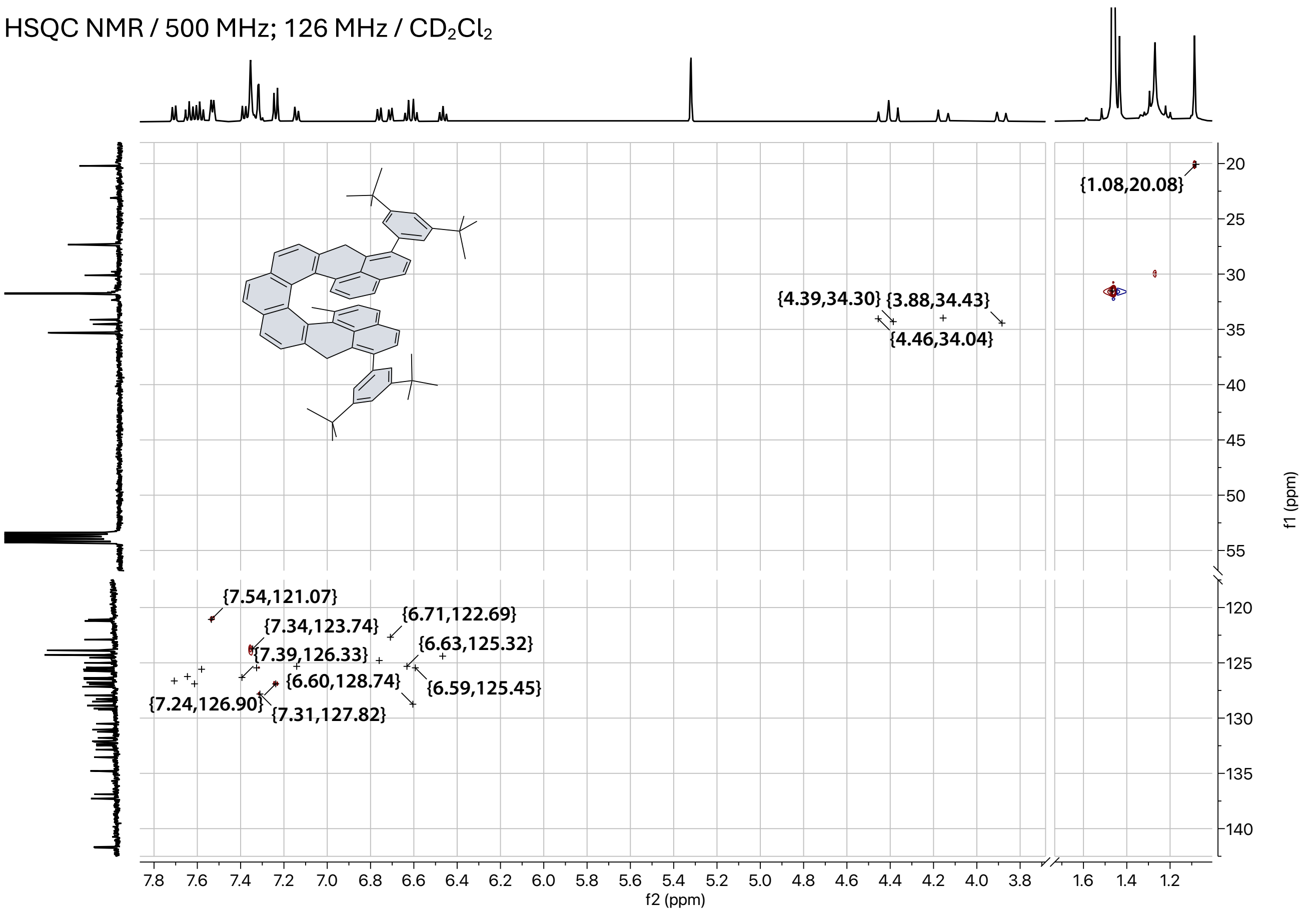

(16a*S*,16b*S*)-3,12-Bis(3,5-di-*tert*-butylphenyl)-16a-methyl-16a*H*-dibenzo[*cd*,*ghi*]phenaleno[2,1-*p*]perylene (*c*-MNC)

^{1}H NMR / 500 MHz / C_6D_6

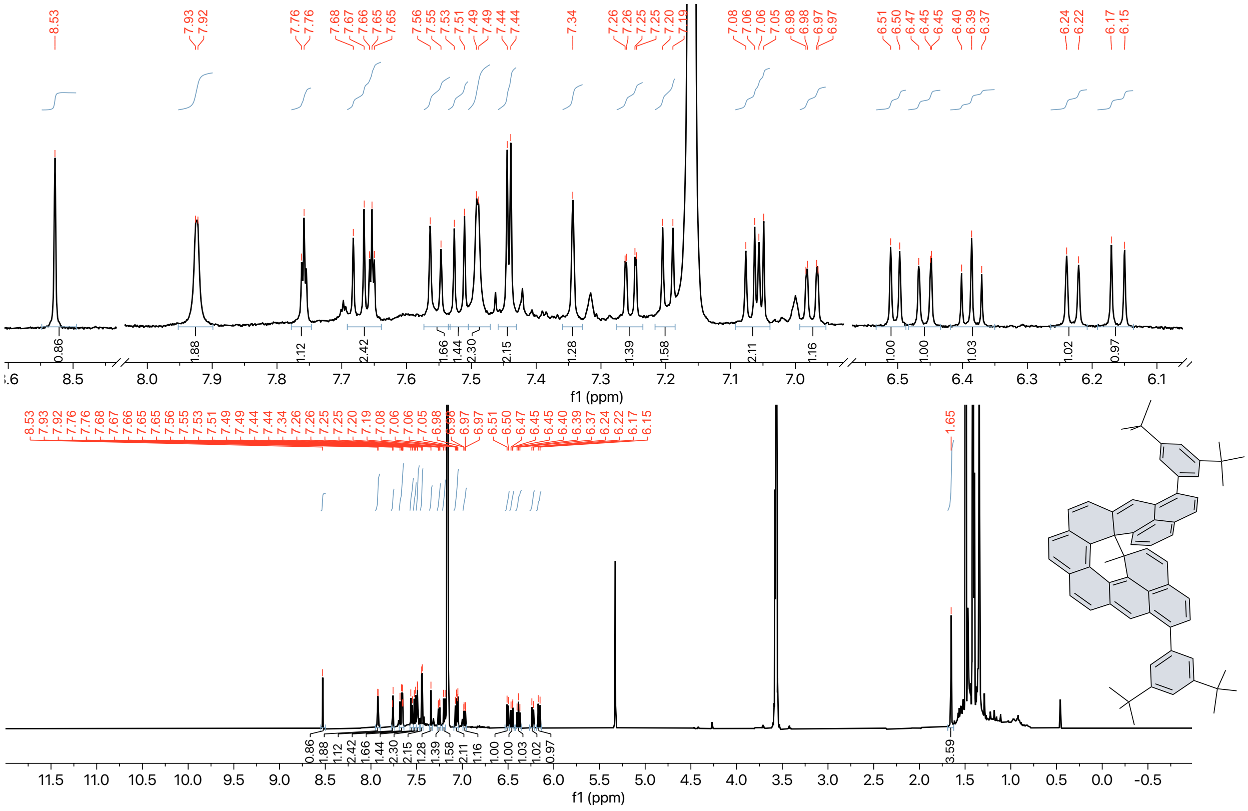

COSY NMR / 500 MHz; 126 MHz / C_6D_6

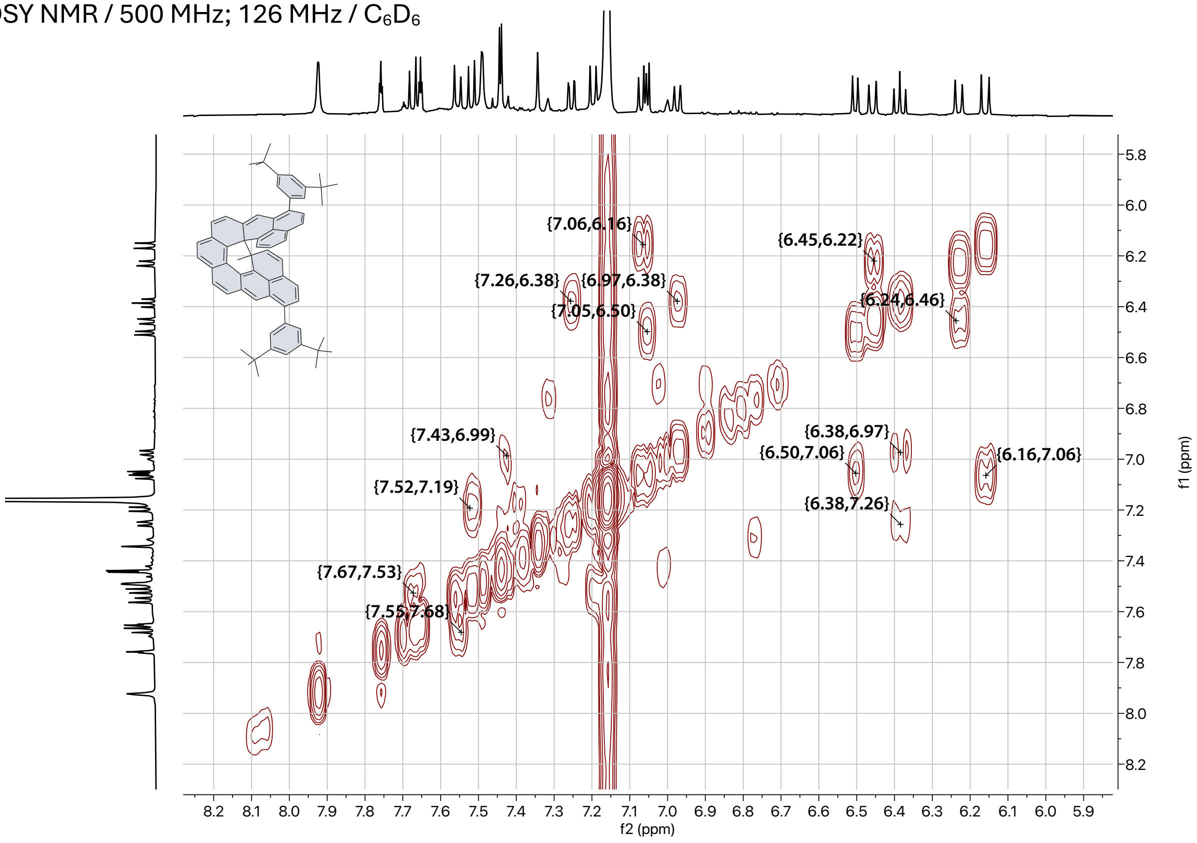

HMBC NMR / 500 MHz, 126 MHz / C_6D_6

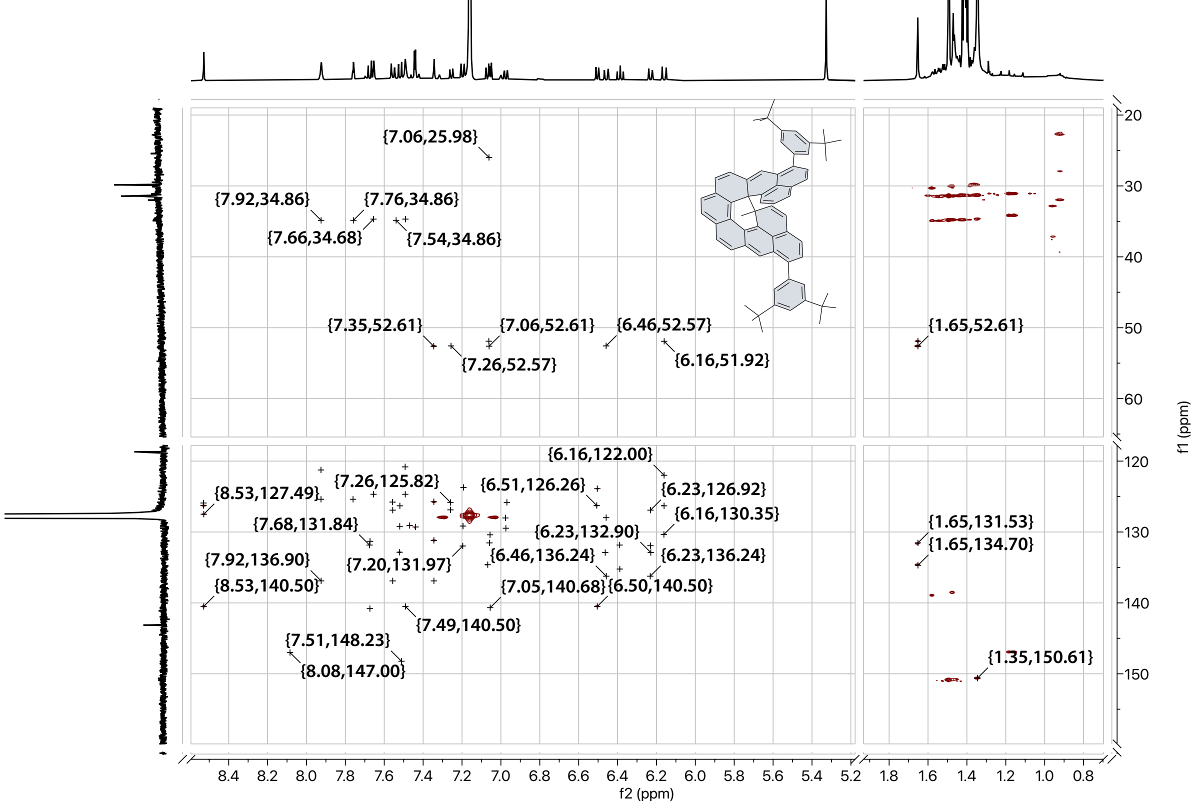

HMBC NMR / 500 MHz; 126 MHz / C_6D_6

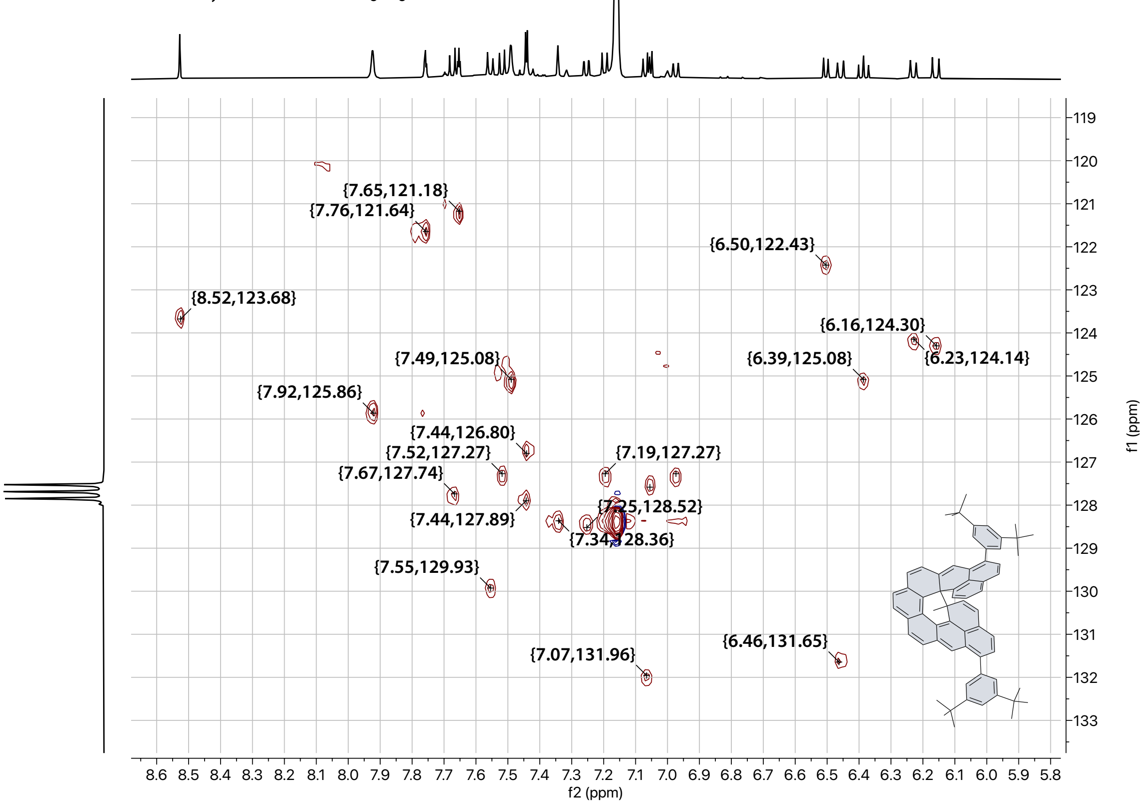

5. Copies of the HRMS Spectra

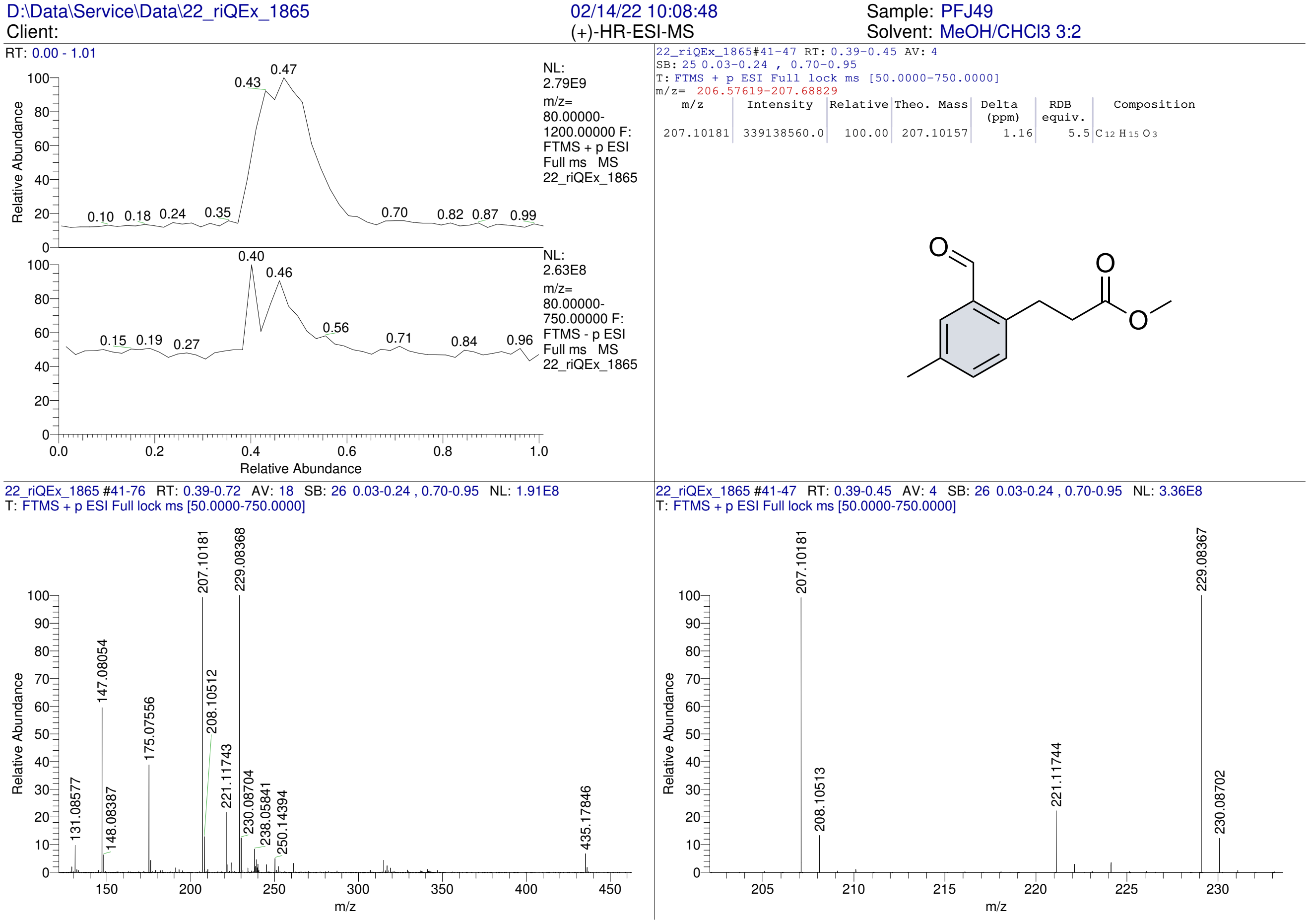

Figure S10. HR-ESI of methyl 3-(2-formyl-4-methylphenyl)propanoate (**10**).

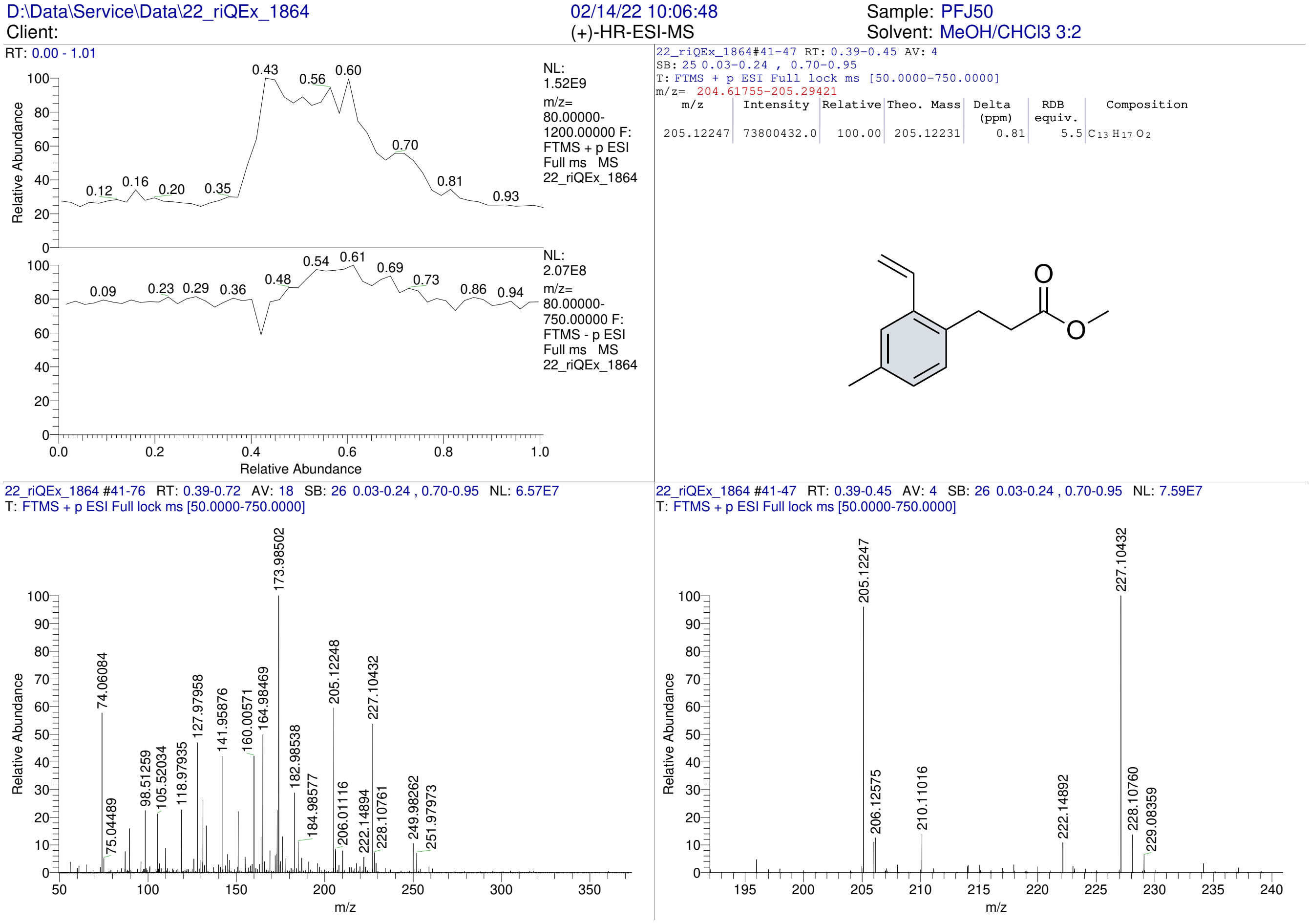

Figure S11. HR-ESI of methyl 3-(4-methyl-2-vinylphenyl)propanoate (**4**).

D:\Data\Service\Data\22_riQEx_1871 — 02/14/22 10:20:51 — Sample: PFJ51
Client: — (+)-HR-ESI-MS — Solvent: MeOH/CHCl3 3:2

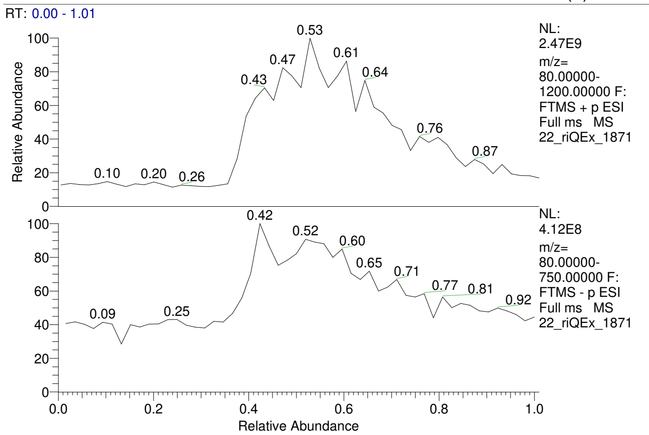

22_riQEx_1871#41-47 RT: 0.39-0.45 AV: 4
SB: 25 0.04-0.24 , 0.70-0.95
T: FTMS + p ESI Full lock ms [100.0000-1500.0000]
m/z= 443.78347-444.37883

m/z	Intensity	Relative	Theo. Mass	Delta (ppm)	RDB equiv.	Composition
444.07214	5095018.0	100.00	444.07194	0.45	16.0	$C_{26}H_{21}O_2Br$
			444.07245	-0.69	3.5	$C_{12}H_{23}O_8N_5Br$
			444.07111	2.33	4.0	$C_{10}H_{21}O_7N_8Br$
			444.07060	3.47	16.5	$C_{24}H_{19}ON_3Br$
			444.07379	-3.71	8.5	$C_{13}H_{19}O_4N_9Br$
			444.07379	-3.72	3.0	$C_{14}H_{25}O_9N_2Br$

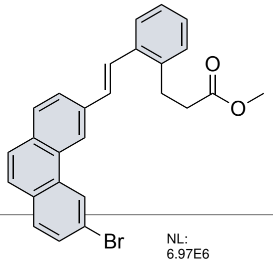

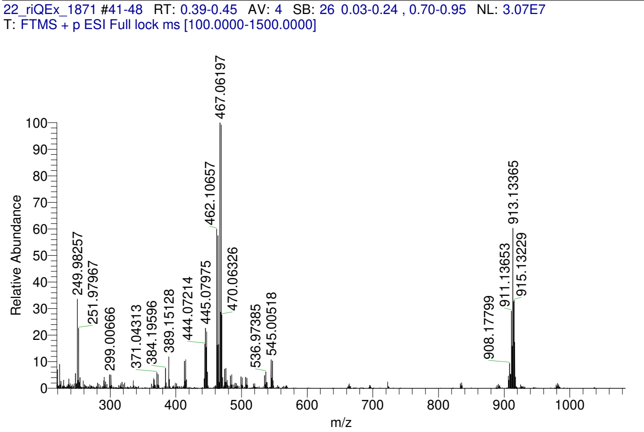

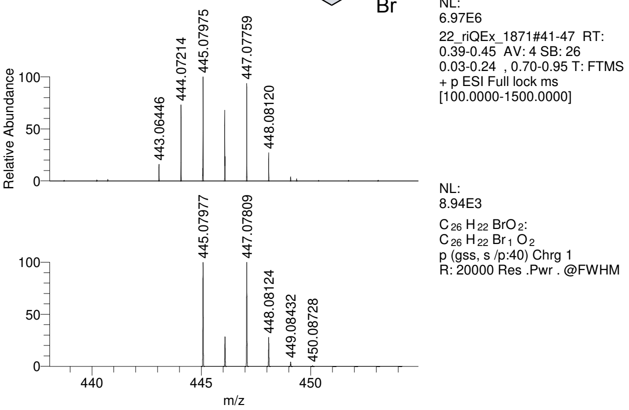

Figure S12. HR-ESI of methyl (*E*)-3-(2-(2-(6-bromophenanthren-3-yl)vinyl)phenyl) propanoate (**3**).

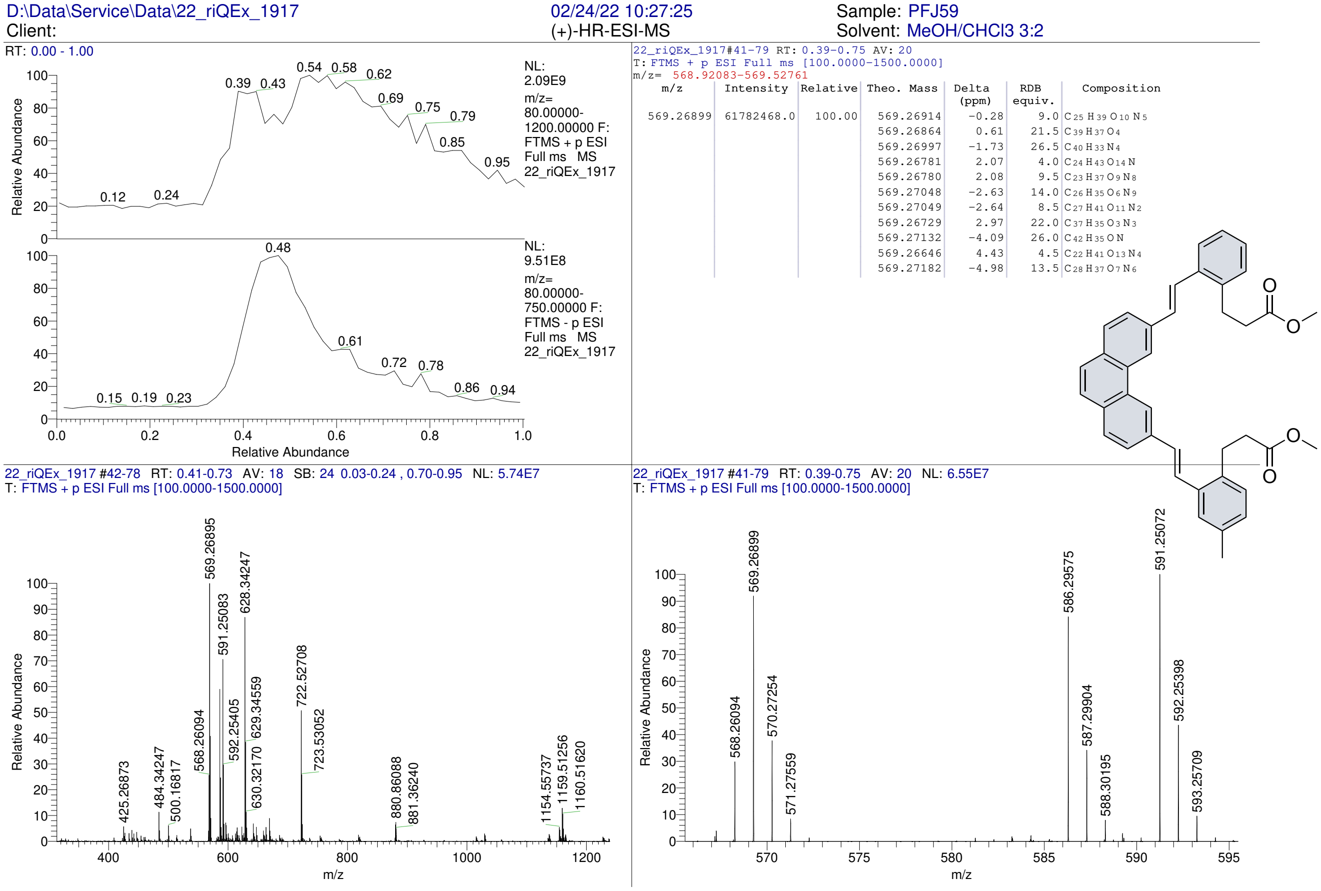

m/z	Intensity	Relative	Theo. Mass	Delta (ppm)	RDB equiv.	Composition
569.26899	61782468.0	100.00	569.26914	-0.28	9.0	$C_{25}H_{39}O_{10}N_5$
			569.26864	0.61	21.5	$C_{39}H_{37}O_4$
			569.26997	-1.73	26.5	$C_{40}H_{33}N_4$
			569.26781	2.07	4.0	$C_{24}H_{43}O_{14}N$
			569.26780	2.08	9.5	$C_{23}H_{37}O_9N_8$
			569.27048	-2.63	14.0	$C_{26}H_{35}O_6N_9$
			569.27049	-2.64	8.5	$C_{27}H_{41}O_{11}N_2$
			569.26729	2.97	22.0	$C_{37}H_{35}O_3N_3$
			569.27132	-4.09	26.0	$C_{42}H_{35}ON$
			569.26646	4.43	4.5	$C_{22}H_{41}O_{13}N_4$
			569.27182	-4.98	13.5	$C_{28}H_{37}O_7N_6$

Figure S13. HR-ESI of dimethyl 3,3'-(((1*E*,1'*E*)-phenanthrene-3,6-diylbis(ethene-2,1-diyl))bis(2,1-phenylene))dipropionate methyl (**5**).

D:\Data\...\22_riQEx_1916_20220224110215 02/24/22 11:03:13 Sample: PFJ58
Client: (+)-HR-ESI-MS Solvent: MeOH/CHCl3 3:2

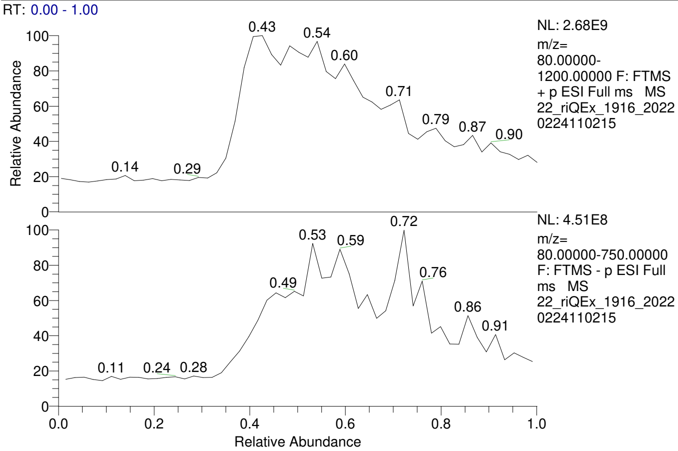

22_riQEx_1916_20220224110215#41-47 RT: 0.39-0.45 AV: 4
SB: 25 0.03-0.24 , 0.69-0.94
T: FTMS + p ESI Full ms [100.0000-1500.0000]
m/z= 564.89177-565.48309

m/z	Intensity	Relative	Theo. Mass	Delta (ppm)	RDB equiv.	Composition
565.23790	48350200.0	100.00	565.23784	0.10	11.0	$C_{25}H_{35}O_{10}N_5$
			565.23734	1.00	23.5	$C_{39}H_{33}O_4$
			565.23867	-1.37	28.5	$C_{40}H_{29}N_4$
			565.23918	-2.26	16.0	$C_{26}H_{31}O_6N_9$
			565.23919	-2.27	10.5	$C_{27}H_{37}O_{11}N_2$
			565.23651	2.47	6.0	$C_{24}H_{39}O_{14}N$
			565.23650	2.48	11.5	$C_{23}H_{33}O_9N_8$
			565.23599	3.38	24.0	$C_{37}H_{31}O_3N_3$
			565.24002	-3.74	28.0	$C_{42}H_{31}ON$
			565.24052	-4.64	15.5	$C_{28}H_{33}O_7N_6$
			565.23516	4.84	6.5	$C_{22}H_{37}O_{13}N_4$

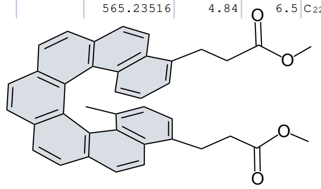

22_riQEx_1916_20220224110215 #41-49 RT: 0.39-0.46 AV: 5 SB: 24 0.03-0.24 , 0.70-0.95 NL:
T: FTMS + p ESI Full ms [100.0000-1500.0000]

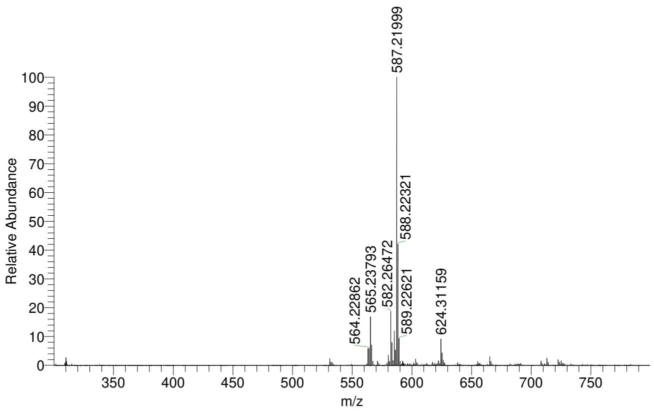

22_riQEx_1916_20220224110215 #41-47 RT: 0.39-0.45 AV: 4 SB: 24 0.03-0.24 , 0.70-0.95 NL:
T: FTMS + p ESI Full ms [100.0000-1500.0000]

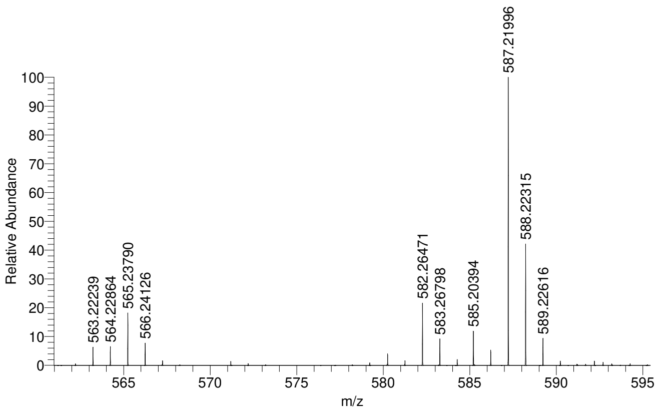

Figure S14. HR-ESI of dimethyl 3,3'-(1-methylbenzo[1,2-*c*:4,3-*c*']diphenanthrene-4,15-diyl)dipropionate (**6**).

HR-ESI Report

Analysis Info

Analysis Name	D:\Data\UZH_Data\Data_2022\Service\Data\22_rihres_0024.d	Acquisition Date	3/7/2022 8:46:48 AM
Method	Service_Syringe_Pump_High_Mass_Range_pos.m	Operator	Demo User
Sample Name	PFJ63	Instrument	timsTOF Pro 1854399.00195
Comment	Solvent: MeOH + NaI<br>Client: Pfister		

Acquisition Parameter

Source Type	ESI	Ion Polarity	Positive	Set Nebulizer	0.4 Bar
Focus	Not active			Set Dry Heater	200 °C
Scan Begin	50 m/z	Set Capillary	4500 V	Set Dry Gas	3.5 l/min
Scan End	3000 m/z	Set End Plate Offset	-500 V	Set Divert Valve	Source

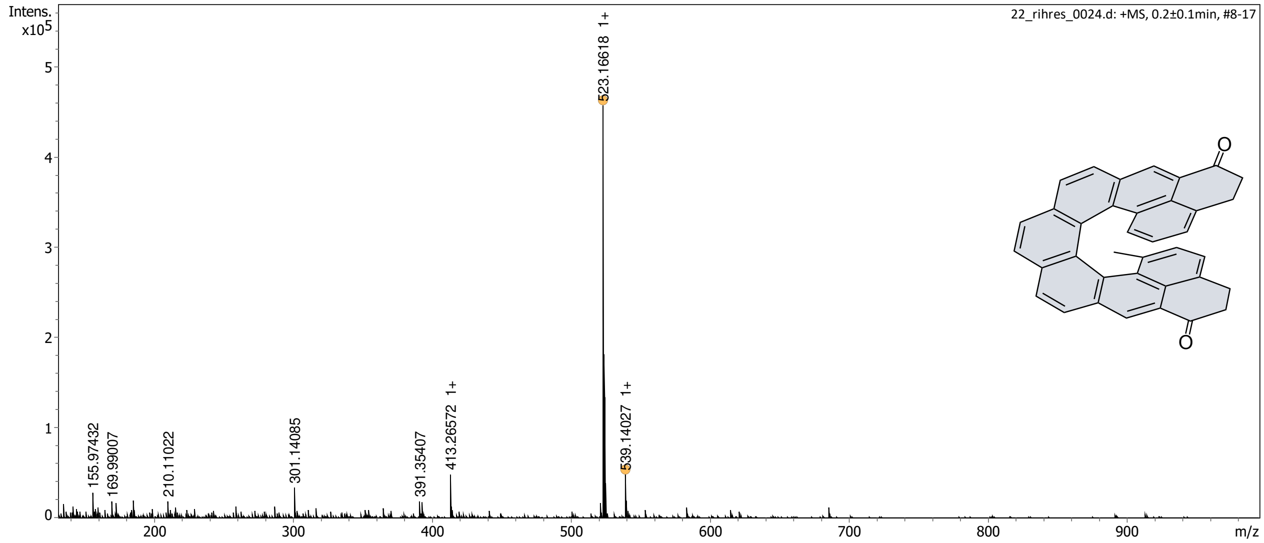

Bruker Compass DataAnalysis 5.3 printed: 3/7/2022 8:50:45 AM by: demo 1 of 2

Figure S15. HR-ESI of methyl-4,5,16,17-tetrahydrobenzo[*no*]benzo[8,9]anthra[1,2-*a*]tetraphene-6,15-dione (**7**).

D:\Data\Service\Data\22_juQEx_2003　　03/10/22 10:25:28
Sample: PFJ65　　(+)-HR-APCI-MS　　Solvent: DCM,　Sheath liquid: MeOH + 0.1% FA　Client: Pfister

22_juQEx_2003 #128-253 RT: 0.30-0.59 AV: 126 NL: 1.96E7
T: FTMS + p APCI corona Full lock ms [150.0000-2000.0000]

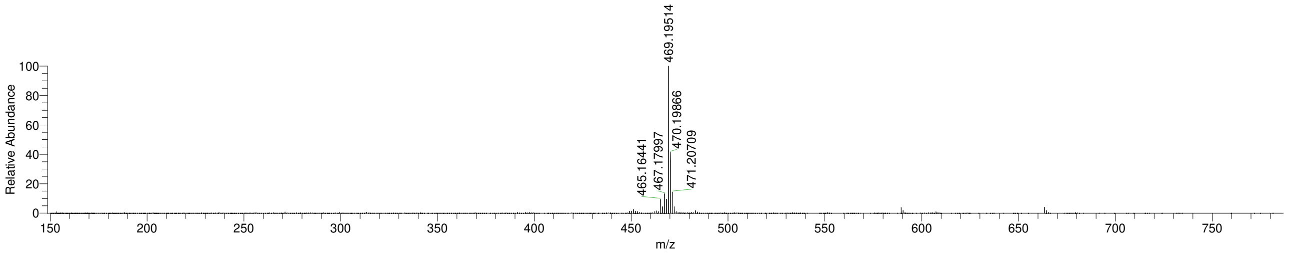

22_juQEx_2003 #128-253 RT: 0.30-0.59 AV: 126 NL: 1.96E7
T: FTMS + p APCI corona Full lock ms [150.0000-2000.0000]

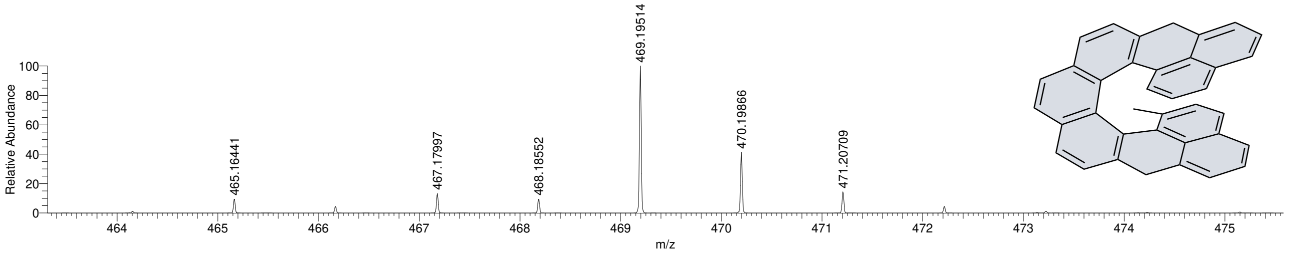

22_juQEx_2003#128-253 RT: 0.30-0.59 AV: 126
T: FTMS + p APCI corona Full lock ms [150.0000-2000.0000]
m/z= 468.89962-469.43373

m/z	Intensity	Relative	Theo. Mass	Delta (ppm)	RDB equiv.	Composition
469.19514	19810204.0	100.00	469.19508	0.14	25.5	$C_{37}H_{25}$

Figure S16. HR-APCI of 1-methyl-7,14-dihydrobenzo[*no*]benzo[8,9]anthra[1,2-*a*]tetraphene (**8**).

D:\Data\Service\Data\25_juQEx_0717 07/01/25 13:07:59
Sample: PFJ266 (+)-HR-APCI-MS Solvent: DCM Sheath liquid: MeOH + 0.1% FA Client: Pfister

25_juQEx_0717 #15-704 RT: 0.03-1.64 AV: 690 SB: 186 2.53-2.96 NL: 1.38E7
T: FTMS + p APCI corona Full ms [80.0000-1200.0000]

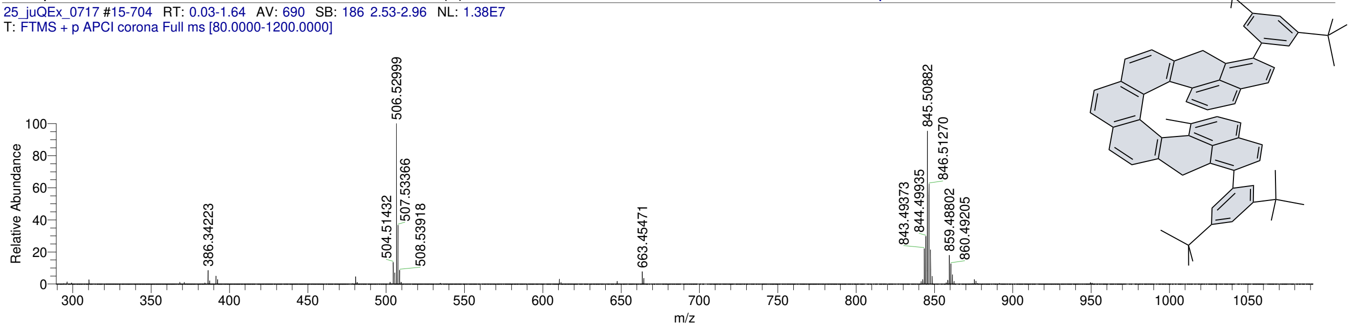

25_juQEx_0717 #15-704 RT: 0.03-1.64 AV: 690 SB: 186 2.53-2.96 NL: 1.32E7
T: FTMS + p APCI corona Full ms [80.0000-1200.0000]

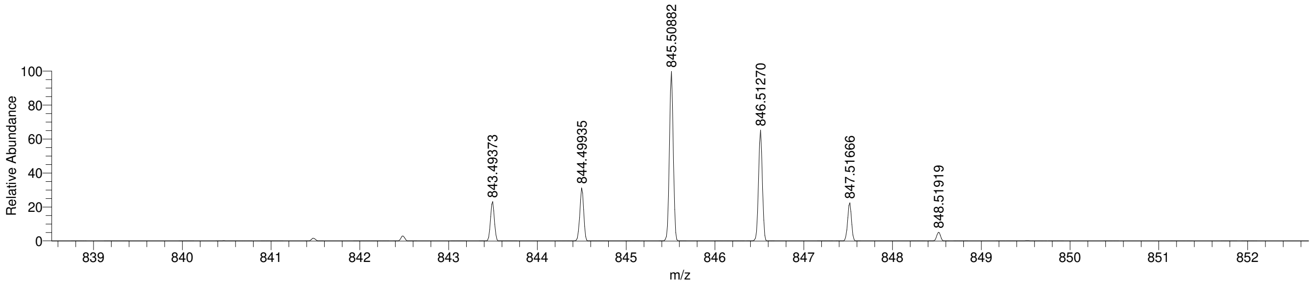

25_juQEx_0717#15-704 RT: 0.03-1.64 AV: 690
SB: 186 2.53-2.96
T: FTMS + p APCI corona Full ms [80.0000-1200.0000]
m/z= 842.79787-845.90907

m/z	Intensity	Relative	Theo. Mass	Delta (ppm)	RDB equiv.	Composition
843.49373	3097347.0	23.37	843.49243	1.54	34.5	$C_{65}H_{63}$
844.49935	4156549.8	31.37	844.50025	-1.06	34.0	$C_{65}H_{64}$
845.50882	13252086.0	100.00	845.50808	0.88	33.5	$C_{65}H_{65}$

Figure S17. HR-APCI of 6,15-bis(3,5-di-*tert*-butylphenyl)-1-methyl-7,14-dihydrobenzo[*no*]benzo[8,9]anthra[1,2-*a*]tetraphene (**9**).

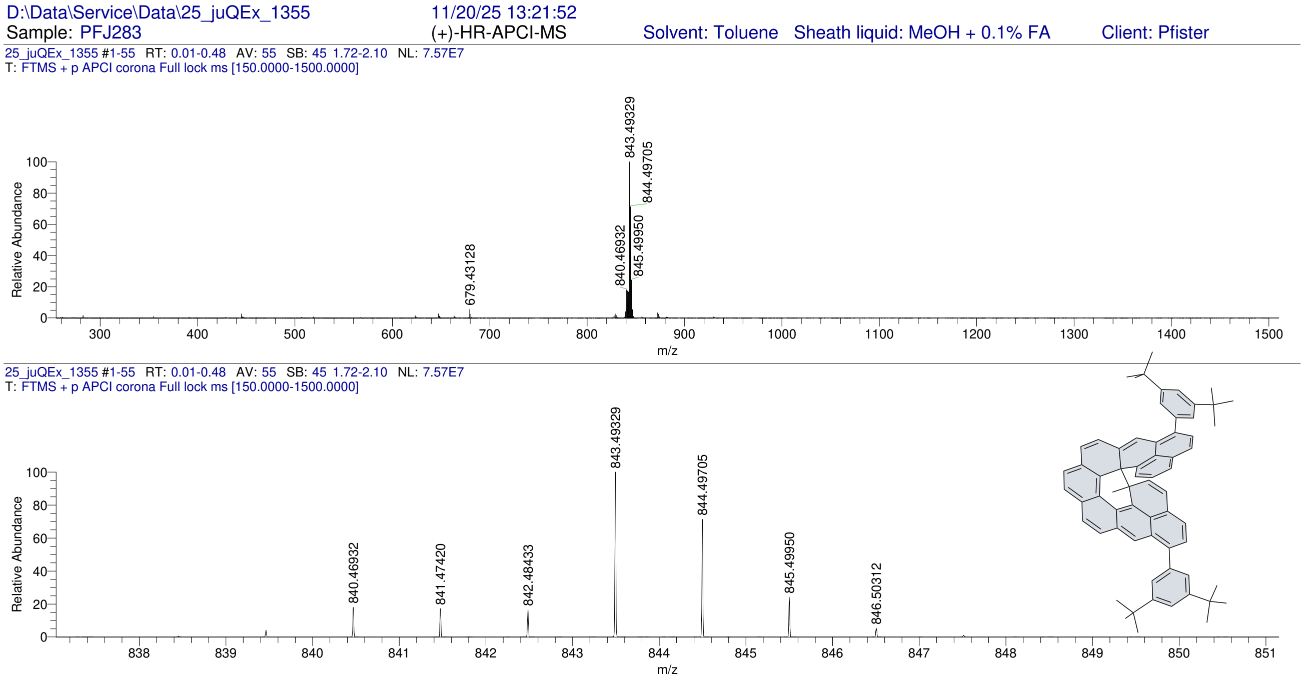

25_juQEx_1355#1-55 RT: 0.01-0.48 AV: 55
SB: 45 1.72-2.10
T: FTMS + p APCI corona Full lock ms [150.0000-1500.0000]
m/z= 843.34830-843.63644

m/z	Intensity	Relative	Theo. Mass	Delta (ppm)	RDB equiv.	Composition
843.49329	77081648.0	100.00	843.49243	1.02	34.5	$C_{65}H_{63}$

Figure S18. HR-APCI of the mixture of *c*-**MNC** and **MNC**.

6. EPR Spectroscopy

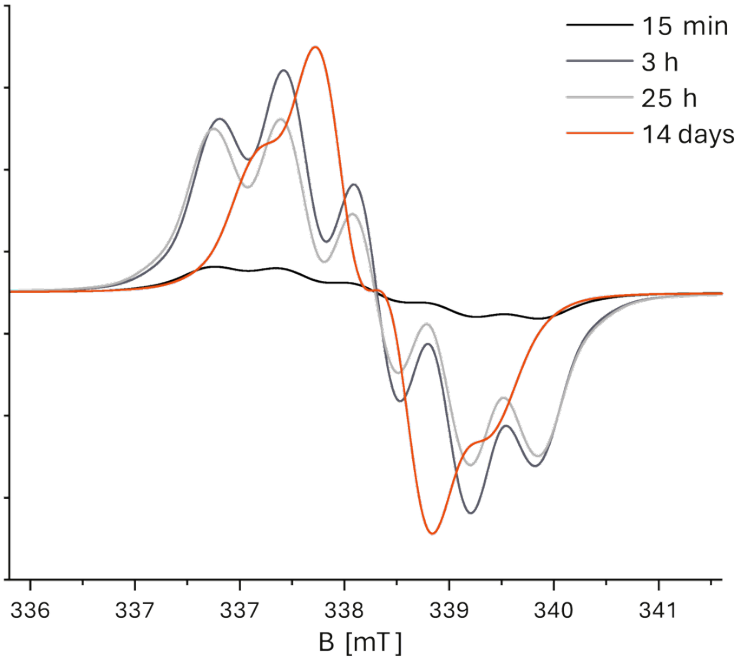

Figure S19. EPR spectra of a nitrogen-saturated solution of **9** in toluene-d_8 after the addition of 1.1 equivalents of *p*-chloranil. The spectra were measured over a period of two weeks. The grey spectra correspond to monoradical **MNC** and the orange spectrum to **MNC** (for calculations, see Schemes S1 and S2, and Tables S8, S9, and S10). The intensity of the orange spectrum was adjusted for better visibility. The signal is centered at g = 2.0026.

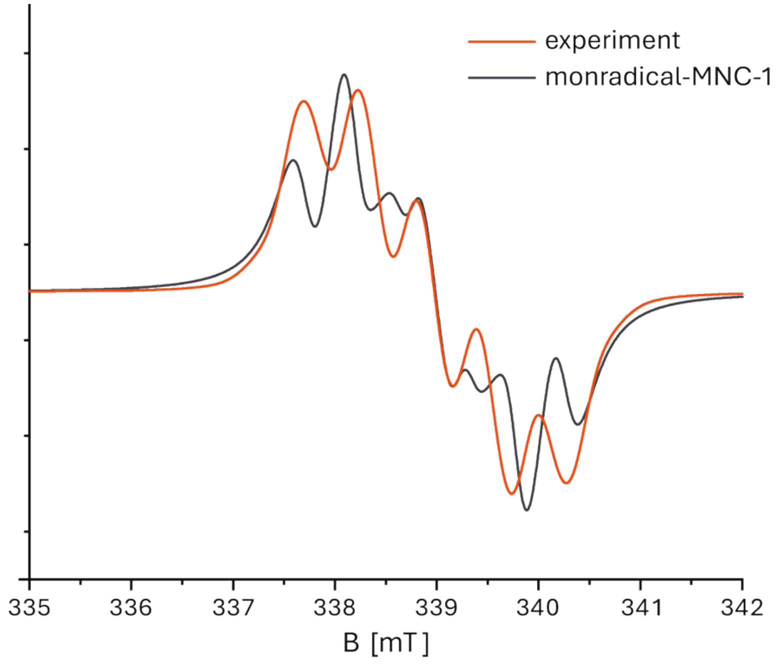

Figure S20. Orange: EPR spectrum of a sample of **9** dissolved in a nitrogen-saturated solution of toluene-d_8 40 min after addition of 1.1 equivalents of *p*-chloranil. Gray: simulated EPR spectrum of monoradical **MNC**-1 using hyperfine coupling constants shown in Scheme S1.

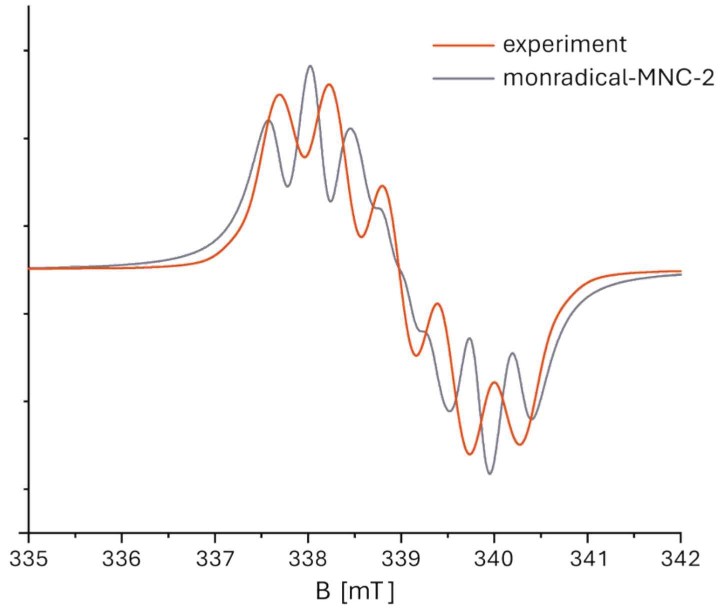

Figure S21. Orange: EPR spectrum of a sample of **9** dissolved in a nitrogen-saturated solution of toluene-d_8 40 min after addition of 1.1 equivalents of *p*-chloranil. Gray: simulated EPR spectrum of monoradical **MNC**-2 using hyperfine coupling constants shown in Scheme S1.

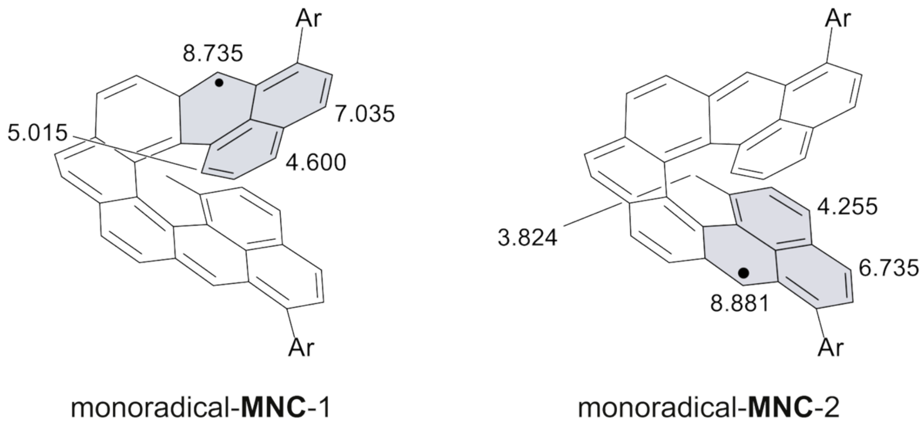

Scheme S1. Structures of monoradicals **MNC**-1 and **MNC**-2 with shown hyperfine coupling parameters that were used for the EPR simulation using WinSim. For calculations, see Table S8 (monoradical **MNC**-1) and Table S9 (monoradical **MNC**-2).

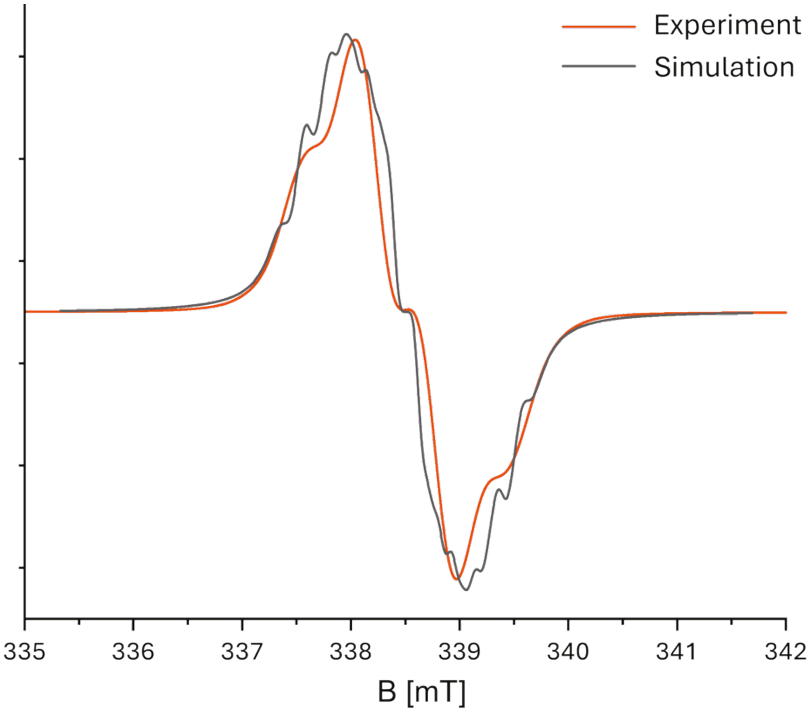

Figure S22. Orange: EPR spectrum of a sample of **MNC** in a nitrogen-saturated solution of toluene-d_8. Gray: simulated EPR spectrum of diradicaloid **MNC**; hyperfine coupling values used for the simulation are shown in Scheme S2.

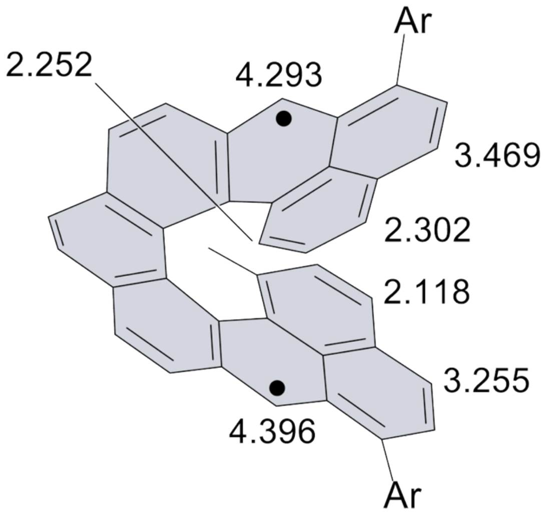

Scheme S2. The diradical resonance structure of **MNC** with shown hyperfine coupling parameters used for the EPR simulation using WinSim. For calculations, see also Table S10.

7. UV–Vis–NIR Spectroscopy and Irradiation Studies

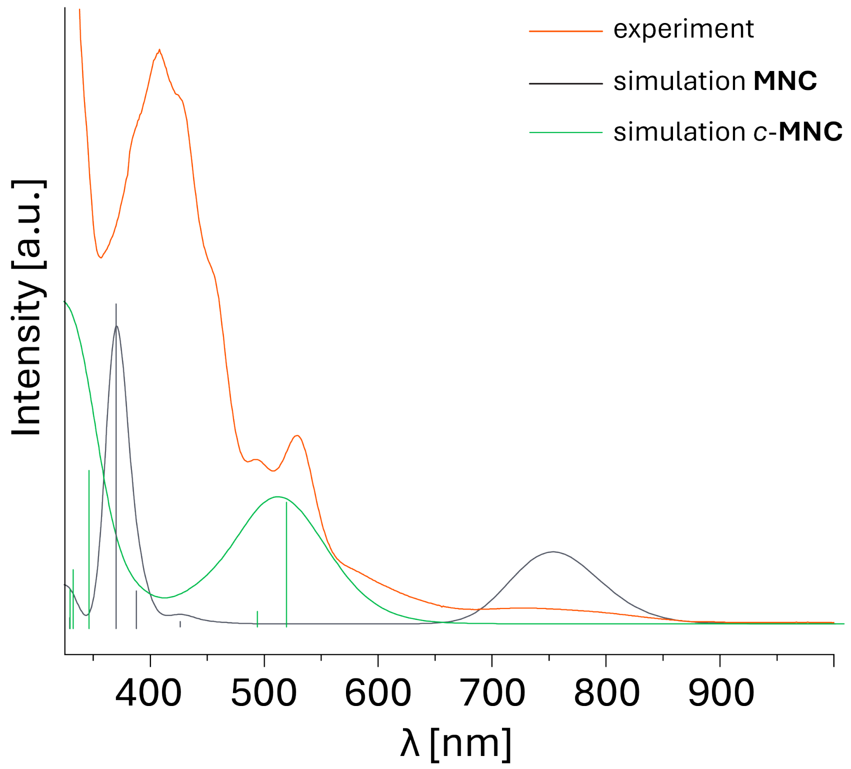

Figure S23. Experimental spectrum of the mixture of *c*-**MNC** and **MNC** in nitrogen-saturated toluene (orange) and simulated spectra by TD-DFT calculations (grey and green lines, and vertical bars; M06-2X-D3/cc-pVTZ). The calculated spectrum of **MNC** is shifted by +0.6 eV and the one of *c*-**MNC** by –0.7 eV.

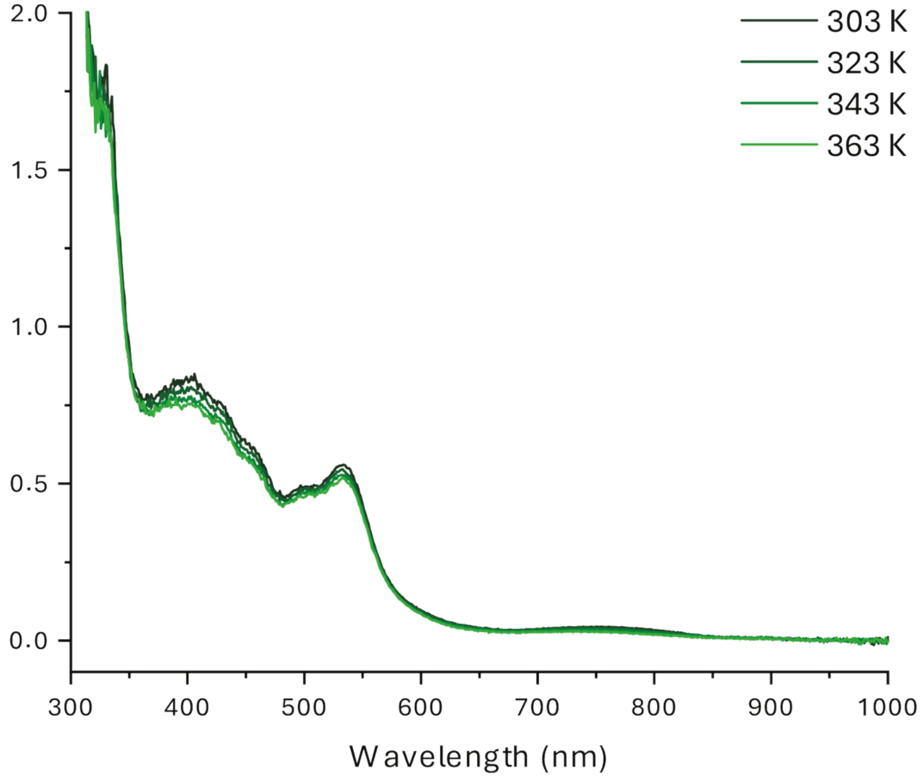

Figure S24. Variable temperature UV–vis–NIR absorption spectra of **9** after the addition of 1.1 equivalents of *p*-chloranil in nitrogen-saturated toluene.

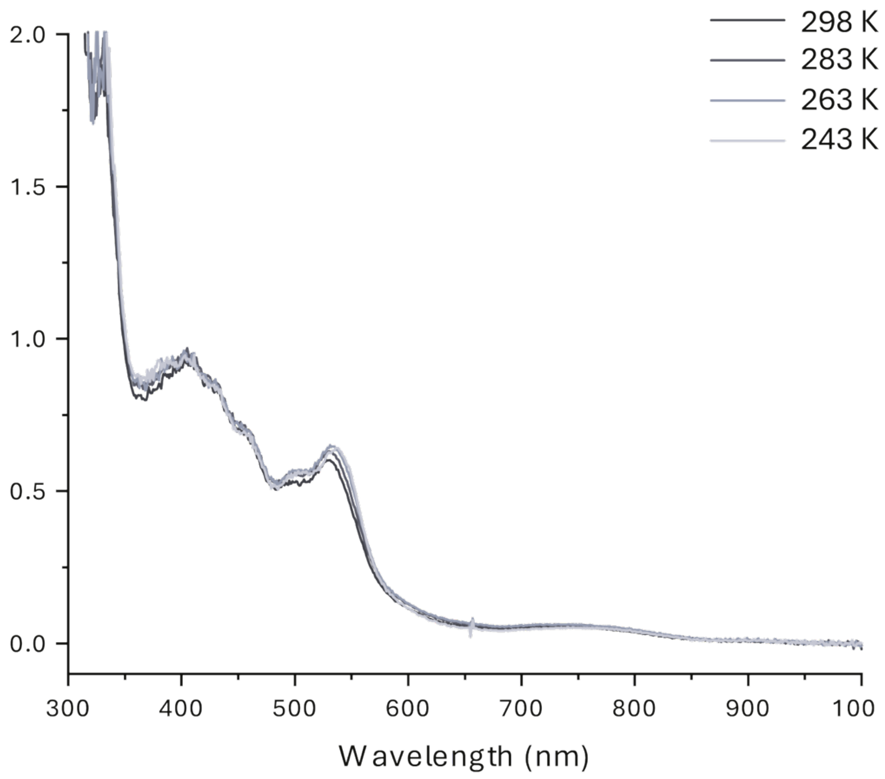

Figure S25. Variable temperature UV–vis–NIR absorption spectra of **9** after the addition of 1.1 equivalents of *p*-chloranil in nitrogen-saturated toluene.

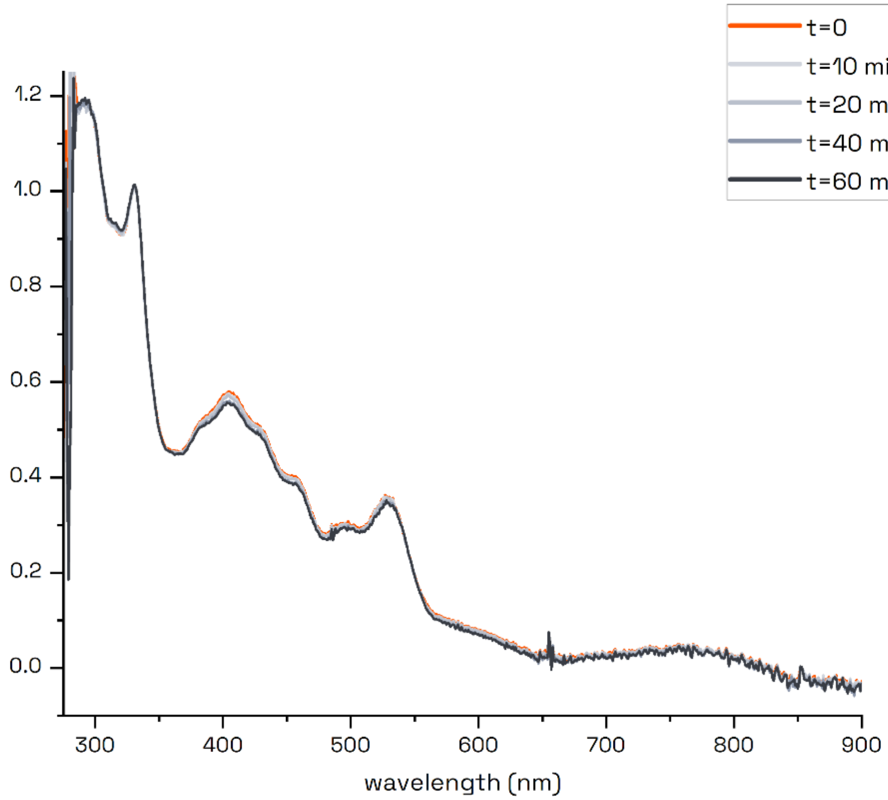

Figure S26. Irradiation of the mixture of *c*-**MNC** and **MNC** in nitrogen-saturated toluene while irradiating with light (505 nm) over a period of 60 min.

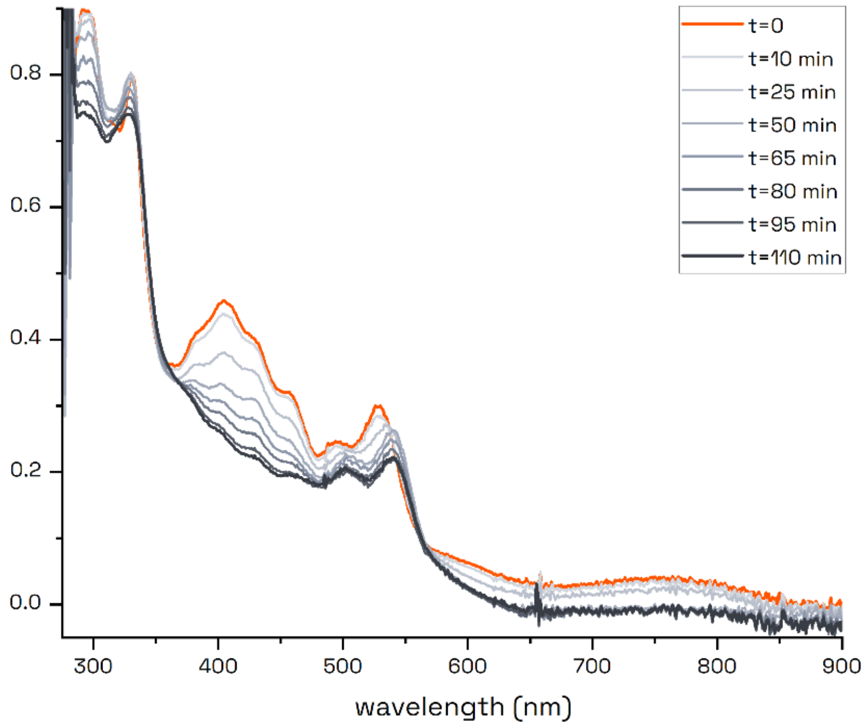

Figure S27. Irradiation of the mixture of *c*-**MNC** and **MNC** in nitrogen-saturated toluene while irradiating with light (405 nm) over a period of 110 min.

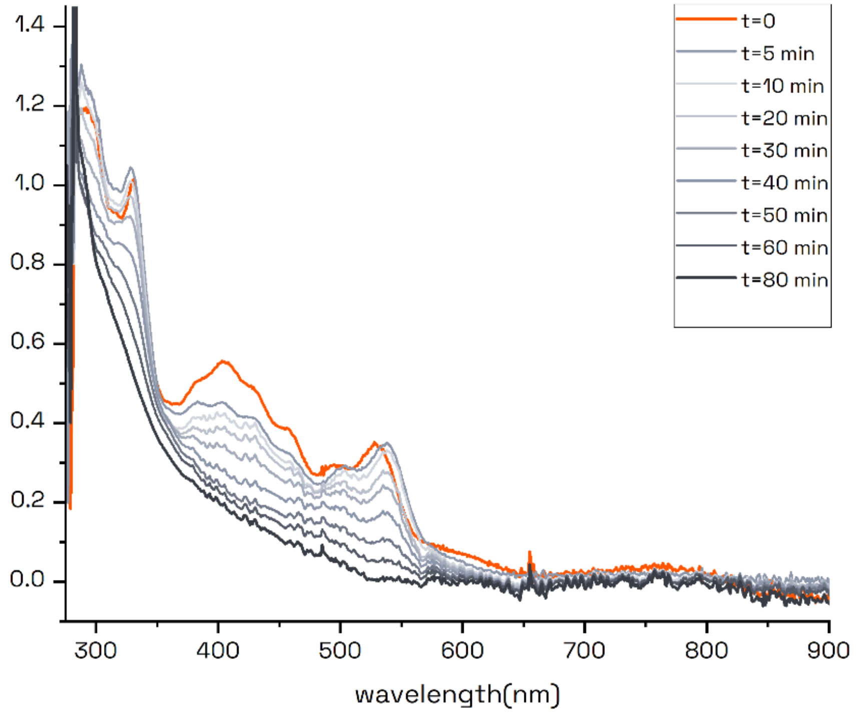

Figure S28. Irradiation of the mixture of *c*-**MNC** and **MNC** in nitrogen-saturated toluene while irradiating with light (365 nm) over a period of 80 min.

8. X-Ray Crystallography

Single-crystal X-ray diffraction data were collected at 160(1) K on a Rigaku OD SuperNova/Atlas area-detector diffractometer using Cu K_α radiation (λ = 1.54184 Å) from a micro-focus X-ray source and an Oxford Instruments Cryojet XL cooler. The selected suitable single crystal was mounted using polybutene oil on a flexible loop fixed on a goniometer head and immediately transferred to the diffractometer. Pre-experiment, data collection, data reduction and analytical absorption correction[3] were performed with the program suite CrysAlisPro.[4] Using Olex2,[5] the structure was solved with the SHELXT[6] small-molecule structure solution program and refined with the SHELXL2018/3 program package[7] by full-matrix least-squares minimization on F^2. PLATON[8] was used to check the results of the X-ray analyses. For more details about the data collection and refinement parameters, see the CIF file.

Yellow crystals were obtained by slow evaporation of CH_2Cl_2 at 25 °C for the SC-XRD measurement.

Special features

The molecule shows two different orientations in the crystal. It results that the methyl group is observed over two sets of positions with site-occupancy factors of 0.429(7) and 0.571(7).

Table S1: *Crystal data and structure refinement for **7**.*

CCDC number	2495359
Empirical formula	$C_{37}H_{24}O_2$
Formula weight	500.56
Temperature/K	160.0(1)
Crystal system	triclinic
Space group	*P*-1
a/Å	8.0938(4)
b/Å	12.2842(6)
c/Å	13.4252(6)
α/°	71.692(4)
β/°	73.626(4)
γ/°	76.024(4)
Volume/Å^3	1198.58(11)
Z	2
ρ_{calc} g/cm^3	1.387
μ/mm^{-1}	0.659
F(000)	524.0
Crystal size/mm^3	0.14 × 0.06 × 0.03
Radiation	Cu K_α (λ = 1.54184)
2Θ range for data collection/°	7.116 to 148.76
Index ranges	$-10 \le h \le 10$, $-14 \le k \le 15$, $-16 \le l \le 16$
Reflections collected	24191
Independent reflections	4872 [R_{int} = 0.0375, R_{sigma} = 0.0254]
Data/restraints/parameters	4872/0/364
Goodness-of-fit on F^2	1.087
Final *R* indexes [*I*>=2σ (*I*)]	R_1 = 0.0676, wR_2 = 0.1842
Final *R* indexes [all data]	R_1 = 0.0810, wR_2 = 0.1925
Largest diff. peak/hole / e Å^{-3}	0.28/-0.25

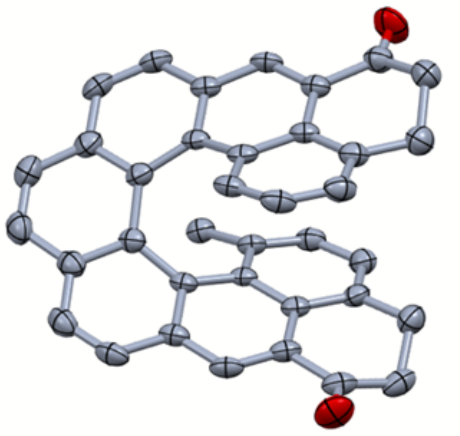

Figure S29. Solid-state structure of **7**. Hydrogen atoms are omitted for clarity. Thermal ellipsoids are shown at the 50% probability level. Color code: C / gray, O / red.

9. DFT Calculations

DFT calculations were performed using Gaussian 16 suite.[9] Geometries were optimized using the functionals listed in Tables S2–S7 and cc-pVTZ basis set in the gas phase. The frequency analysis was performed to verify the stationary-state geometry, where no imaginary frequency was found (except for transition-state calculations, where one imaginary frequency is present). TD-DFT calculations were performed on the M06-2X-D3/cc-pVTZ-optimized geometries at the M06-2X-D3/cc-pVTZ level of theory. The effect of the solvent was accounted for using CPCM (with toluene as solvent). The *tert*-butyl substituents were approximated as methyl groups for lower computational cost. For the transition state calculations, they were omitted completely. For **MNC**, the singlet geometries were optimized with spin-restricted and spin-unrestricted broken-symmetry wavefunctions, whereas the triplet geometry was optimized with spin-unrestricted wavefunctions. The hyperfine coupling constants were calculated by using unrestricted M06-2X functional. The isotropic EPR spectra were simulated using WinSim software and the used parameters are shown in Tables S7 and S8.[10] The adiabatic singlet–triplet energy gap was calculated using DFT-optimized singlet and triplet geometries.

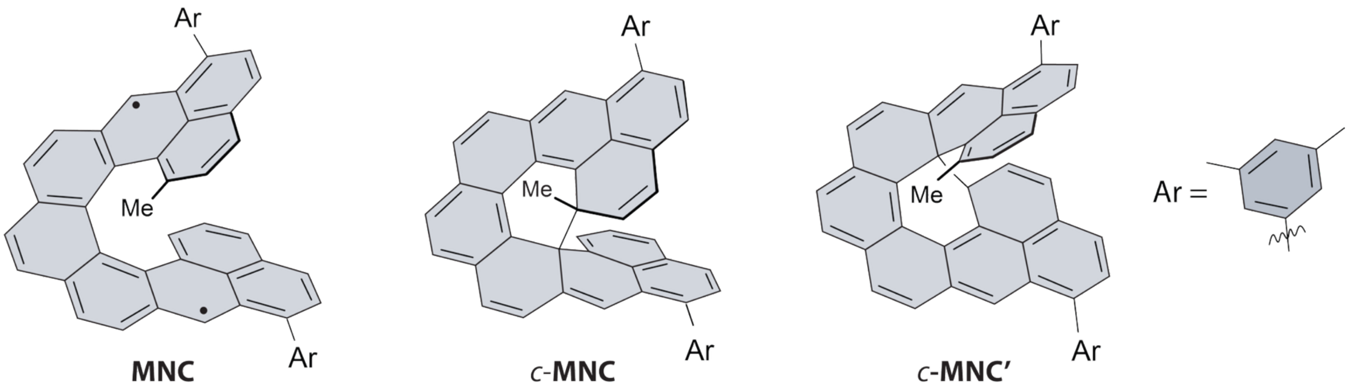

Scheme S3. Structures of the **MNC** derivatives for the calculations of relative energies.

Table S2. Energies [in Hartree] obtained from DFT calculations using cc-pVTZ as basis set including zero-point vibrational energy corrections.

Functional	B3LYP	B3LYP-D3	M06-2X	M06-2X-D3
MNC-S	-2042.50899	-2042.63083	-2041.68699	-2041.69829
MNC-uS*	-2042.52093	-2042.63900	-2041.69989	-2041.71082
MNC-T*	-2042.51953	-2042.63740	-2041.69841	-2041.70926
c-MNC	-2042.51531	-2042.63147	-2041.70718	-2041.71750
c-MNC'	-2042.50775	-2042.62449	-2041.69950**	-2041.71003**

*Calculated with spin-unrestricted broken-symmetry wavefunctions
** One small imaginary frequency (–13 and –9.5 cm^{-1}) was observed for the optimized structure. Visual inspection shows this mode corresponds to nearly free rotation of an outer-sphere methyl group.

Table S3. Relative energies [in kcal/mol] obtained from DFT calculations using cc-pVTZ as basis set including zero-point vibrational energy corrections.

Functional	B3LYP	B3LYP-D3	M06-2X	M06-2X-D3
MNC-S	7.488	5.128	8.091	7.865
MNC-uS*	0	0	0	0
MNC-T*	0.875	1.005	0.932	0.983
c-MNC	3.527	4.728	-4.573	-4.190
c-MNC'	8.271	9.102	0.243	0.496

*Calculated with spin-unrestricted broken-symmetry wavefunctions.

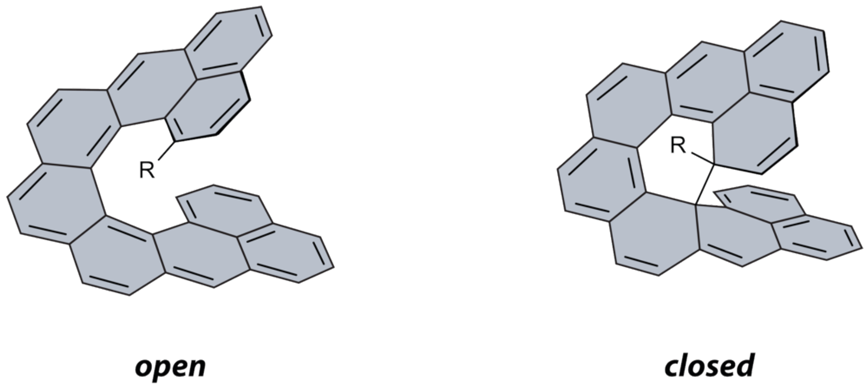

Scheme S4. Structures of open (left) and closed (right) forms with R = Me for **MNC** and R = H for **NC** for the calculations of the transition states.

Transition-state calculations were performed without the Ar substituents to save computational costs and avoid the optimization of modes that correspond to the rotation of outer-sphere methyl groups. Since the transition state between the ground state (unrestricted-singlet calculations) and *c*-**MNC** is of interest, only the singlet states and *c*-**MNC** were calculated.

Table S4. Energies [in Hartree] obtained from DFT calculations for **MNC** using cc-pVTZ as basis set including zero-point vibrational energy corrections of the transition state.

Functional	B3LYP	B3LYP-D3	BMK	M06-2X
open-MNC-S	-1423.196433	-1423.279231	-1422.180216	-1422.639267
open-MNC-uS*	-1423.208334	-1423.288724	-1422.197499	-1422.652981
transition state*	-1423.179445	-1423.279829	n.a.	-1422.658842
closed-MNC	-1423.201037	-1423.262267	-1422.205996	-1422.628287

*Calculated with spin-unrestricted broken-symmetry wavefunctions.

Table S5. Relative energies [in kcal/mol] obtained from DFT calculations for **MNC** using cc-pVTZ as basis set including zero-point vibrational energy corrections of the transition state.

Functional	B3LYP	B3LYP-D3	BMK	M06-2X
open-MNC-S	7.458	5.957	10.845	8.606
open-MNC-uS*	0	0	0	0
transition state*	18.128	16.602	n.a.	15.496
closed-MNC	4.579	5.582	-5.332	-3.678

*Calculated with spin-unrestricted broken-symmetry wavefunctions.

Table S6. Energies [in Hartree] obtained from DFT calculations for **NC** using cc-pVTZ as basis set including zero-point vibrational energy corrections of the transition state.

Functional	B3LYP	B3LYP-D3	BMK	M06-2X
open-MNC-S	-1383.900947	-1383.973822	-1382.910665	-1383.356602
open-MNC-uS*	-1383.915927	-1383.986501	-1382.932385	-1383.373098
transition state*	-1383.887426	-1383.960741	-1382.907907	-1383.350253
closed-MNC	-1383.907341	-1383.978079	-1382.940089	-1383.379251

*Calculated with spin-unrestricted broken-symmetry wavefunctions.

Table S7. Relative energies [in kcal/mol] obtained from DFT calculations for **NC** using cc-pVTZ as basis set including zero-point vibrational energy corrections of the transition state.

**Functional**	**B3LYP**	**B3LYP-D3**	**BMK**	**M06-2X**
open-MNC-S	9.400	7.956	13.629	10.351
open-MNC-uS*	0	0	0	0
transition state*	17.884	16.164	15.360	14.335
closed-MNC	5.388	5.284	-4.834	-3.861

*Calculated with spin-unrestricted broken-symmetry wavefunctions.

Geometry optimization of **DMNC**-Ar and **NC**-Ar were performed for comparison with **MNC** as shown in Figure 2 of the main text. Detailed investigation of these compounds was made in the respective publications. Ar = 3,5-dimethylphenyl.

Table S8. Calculated energies obtained from DFT calculations for **DMNC** and **NC** with the aryl substituents using M06-2X as functional and cc-pVTZ as basis set including zero-point vibrational energy corrections.

	**M06-2X (Hartee)**	**Relative energies (kcal/mol)**
DMNC-Ar-uS	-2080.979186	0
c-DMNC-Ar	-2080.977362	-4.706
NC-Ar-uS	-2002.420123	0
c-NC-Ar	-2002.427623	1.144

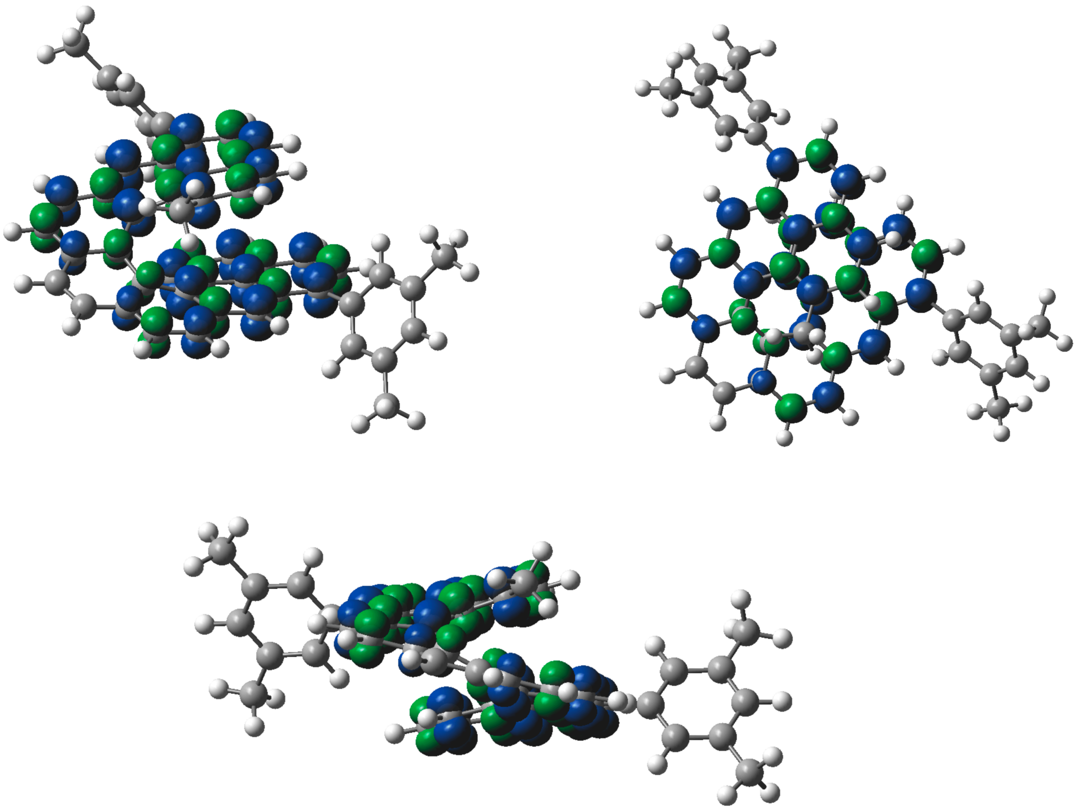

Figure S30. Plot of the spin density of triplet **MNC** calculated by DFT (M06-2X/cc-pVTZ), depicted with an iso-value of 0.05 and a density of 0.025. Three different perspectives are shown for better visibility of the spin densities.

Table S9. Overview of the Mulliken charges, spin densities, and isotropic fermi contact couplings for mon-oradical **MCN**-2 with the radical located on the non-substitutes phenalenyl unit (gray; M06-2X/cc-pVTZ).

Mulliken charges and spin densities

	1	2
1 C	-0.144002	-0.051304
2 C	-0.150463	0.081176
3 C	0.114839	-0.036292
4 C	-0.138577	0.057083
5 C	0.006099	-0.088711
6 C	0.051903	0.110448
7 C	-0.149737	0.015642
8 C	-0.119162	-0.017848
9 C	0.016631	0.018082
10 C	-0.001791	-0.020833
11 C	-0.030503	0.221674
12 C	0.050444	-0.162799
13 C	-0.12734	0.082473
14 C	-0.151468	-0.07551
15 C	0.186854	-0.124443
16 C	-0.010613	0.083172

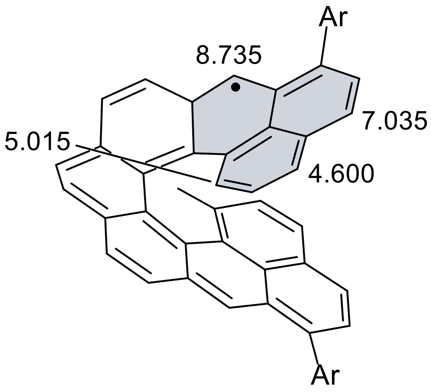

17	C	0.073866	-0.198357
18	C	-0.215094	0.447152
19	C	-0.367401	-0.005797
20	C	0.179765	0.007057
21	C	-0.110249	-0.000025
22	C	-0.0575	0.011762
23	C	0.083267	0.006353
24	C	-0.088344	-0.012589
25	C	-0.20826	0.008846
26	C	0.162865	-0.01272
27	C	-0.108546	-0.002351
28	C	-0.118224	0.00138
29	C	-0.168841	-0.004351
30	C	-0.131171	0.257323
31	C	-0.163678	-0.135975
32	C	-0.161434	0.24221
33	C	0.107873	-0.163903
34	C	-0.164272	0.366678
35	C	-0.138213	-0.187714
36	C	-0.109875	0.359264
37	C	-0.464251	0.005624
38	C	0.049183	-0.03841
39	C	-0.164428	0.023947
40	C	0.098226	-0.01053
41	C	-0.167943	0.021166
42	C	0.105827	-0.011479
43	C	-0.219376	0.022538
44	C	-0.378729	0.000427
45	C	-0.37699	0.000377
46	C	0.05037	-0.000318
47	C	-0.190083	-0.000149
48	C	0.099503	0.000124
49	C	-0.163264	-0.000237
50	C	0.091697	0.000144
51	C	-0.170379	0.000074
52	C	-0.379414	-0.000003
53	C	-0.378874	-0.000007
54	H	0.09761	0.002215
55	H	0.098321	-0.004981
56	H	0.097782	-0.000659
57	H	0.100272	0.001053
58	H	0.099798	-0.003648
59	H	0.099696	0.004129
60	H	0.147517	-0.029056

61	H	0.170772	0.000347
62	H	0.132373	0.00137
63	H	0.115049	-0.001332
64	H	0.126336	-0.000388
65	H	0.111675	-0.000134
66	H	0.160933	-0.01932
67	H	0.121765	0.005561
68	H	0.127457	-0.017721
69	H	0.109196	-0.024735
70	H	0.123197	0.007334
71	H	0.148994	-0.001086
72	H	0.157416	-0.001279
73	H	0.137876	-0.000444
74	H	0.12821	-0.000534
75	H	0.105478	-0.001435
76	H	0.150259	-0.004902
77	H	0.10623	0.000101
78	H	0.124	-0.000263
79	H	0.129723	-0.000202
80	H	0.104658	0.000055
81	H	0.121827	-0.000138
82	H	0.128818	-0.000212
83	H	0.142028	-0.000067
84	H	0.105219	0.000017
85	H	0.132985	0.000085
86	H	0.124598	0.000003
87	H	0.103726	0
88	H	0.129447	0.000002
89	H	0.122034	0.000002
90	H	0.104725	0
91	H	0.128735	0.000003
92	H	0.182549	0.000719

Sum of Mulliken charges = –0.00000 1.00000

Isotropic Fermi Contact couplings

Atom	a.u.	MegaHertz	Gauss	10^{-4} cm^{-1}
1 C(13)	-0.00871	-9.78836	-3.49273	-3.26505
2 C(13)	0.0111	12.4783	4.45257	4.16231
3 C(13)	-0.00677	-7.60746	-2.71453	-2.53757
4 C(13)	0.00873	9.81837	3.50344	3.27506
5 C(13)	-0.01806	-20.30756	-7.24624	-6.77387
6 C(13)	0.01909	21.45762	7.65661	7.15749
7 C(13)	0.00274	3.08529	1.10091	1.02914

8 C(13)	-0.00264	-2.96972	-1.05967	-0.99059
9 C(13)	0.0029	3.26108	1.16363	1.08778
10 C(13)	-0.00345	-3.87941	-1.38427	-1.29403
11 C(13)	0.0383	43.0617	15.36549	14.36384
12 C(13)	-0.03076	-34.58025	-12.33909	-11.53473
13 C(13)	0.01361	15.2975	5.45853	5.1027
14 C(13)	-0.01231	-13.83381	-4.93625	-4.61446
15 C(13)	-0.02604	-29.27882	-10.44741	-9.76636
16 C(13)	0.0153	17.20461	6.13903	5.73884
17 C(13)	-0.0358	-40.2415	-14.35917	-13.42312
18 C(13)	0.06078	68.32482	24.38	22.79071
19 C(13)	-0.00044	-0.49798	-0.17769	-0.16611
20 C(13)	0.00052	0.58453	0.20857	0.19498
21 C(13)	-0.0009	-1.01674	-0.3628	-0.33915
22 C(13)	0.00187	2.10017	0.74939	0.70054
23 C(13)	0.00078	0.87467	0.31211	0.29176
24 C(13)	-0.00079	-0.88364	-0.31531	-0.29475
25 C(13)	0.00105	1.1807	0.4213	0.39384
26 C(13)	-0.00061	-0.68372	-0.24397	-0.22806
27 C(13)	-0.00069	-0.77572	-0.2768	-0.25875
28 C(13)	0.00114	1.27801	0.45602	0.4263
29 C(13)	0.00007	0.08347	0.02978	0.02784
30 C(13)	0.03553	39.937	14.25052	13.32155
31 C(13)	-0.0221	-24.84072	-8.86379	-8.28597
32 C(13)	0.03291	36.99191	13.19963	12.33917
33 C(13)	-0.02645	-29.73898	-10.61161	-9.91986
34 C(13)	0.04894	55.01597	19.63106	18.35135
35 C(13)	-0.03184	-35.79208	-12.7715	-11.93895
36 C(13)	0.05583	62.76854	22.39737	20.93733
37 C(13)	0.00027	0.29874	0.1066	0.09965
38 C(13)	-0.01223	-13.75031	-4.90645	-4.58661
39 C(13)	0.01249	14.03915	5.00952	4.68296
40 C(13)	-0.00067	-0.75182	-0.26827	-0.25078
41 C(13)	0.00273	3.07069	1.0957	1.02427
42 C(13)	-0.0014	-1.57946	-0.56359	-0.52685
43 C(13)	0.00925	10.39389	3.7088	3.46703
44 C(13)	0.00048	0.53627	0.19135	0.17888
45 C(13)	0.00035	0.39391	0.14056	0.1314
46 C(13)	0.00015	0.16851	0.06013	0.05621
47 C(13)	-0.00013	-0.14392	-0.05135	-0.04801
48 C(13)	0.00001	0.01403	0.00501	0.00468
49 C(13)	-0.00003	-0.03222	-0.0115	-0.01075
50 C(13)	0.00002	0.01724	0.00615	0.00575
51 C(13)	-0.0001	-0.1086	-0.03875	-0.03623

52	C(13)	0	-0.00416	-0.00149	-0.00139
53	C(13)	0	-0.00415	-0.00148	-0.00138
54	H(1)	0.00037	1.67276	0.59688	0.55797
55	H(1)	-0.001	-4.47786	-1.59781	-1.49365
56	H(1)	-0.00017	-0.74461	-0.2657	-0.24838
57	H(1)	0.0002	0.88505	0.31581	0.29522
58	H(1)	-0.00097	-4.3181	-1.54081	-1.44036
59	H(1)	0.00081	3.6228	1.29271	1.20844
60	H(1)	-0.00548	-24.47707	-8.73403	-8.16467
61	H(1)	0.00012	0.53484	0.19084	0.1784
62	H(1)	0.00009	0.41807	0.14918	0.13945
63	H(1)	-0.00004	-0.16791	-0.05991	-0.05601
64	H(1)	-0.00005	-0.21282	-0.07594	-0.07099
65	H(1)	0.00011	0.50561	0.18042	0.16865
66	H(1)	-0.00314	-14.05513	-5.01522	-4.68829
67	H(1)	0.00103	4.60038	1.64153	1.53452
68	H(1)	-0.00288	-12.89241	-4.60033	-4.30044
69	H(1)	-0.00441	-19.71733	-7.03563	-6.57699
70	H(1)	0.00125	5.5993	1.99797	1.86773
71	H(1)	-0.00001	-0.03664	-0.01307	-0.01222
72	H(1)	-0.00019	-0.8632	-0.30801	-0.28793
73	H(1)	-0.0002	-0.88861	-0.31708	-0.29641
74	H(1)	-0.00036	-1.61925	-0.57779	-0.54012
75	H(1)	-0.00024	-1.07952	-0.3852	-0.36009
76	H(1)	-0.00027	-1.20935	-0.43153	-0.4034
77	H(1)	-0.00001	-0.03654	-0.01304	-0.01219
78	H(1)	-0.00012	-0.53315	-0.19024	-0.17784
79	H(1)	-0.00011	-0.49762	-0.17756	-0.16599
80	H(1)	0.00001	0.04507	0.01608	0.01503
81	H(1)	-0.00009	-0.41778	-0.14907	-0.13936
82	H(1)	-0.00013	-0.60154	-0.21464	-0.20065
83	H(1)	0	0.01752	0.00625	0.00584
84	H(1)	0	0.01236	0.00441	0.00412
85	H(1)	0	0.01517	0.00541	0.00506
86	H(1)	0	0.0067	0.00239	0.00223
87	H(1)	0	0.00047	0.00017	0.00016
88	H(1)	0	0.00628	0.00224	0.0021
89	H(1)	0	0.00503	0.00179	0.00168
90	H(1)	0	-0.00027	-0.0001	-0.00009
91	H(1)	0	0.007	0.0025	0.00233
92	H(1)	0.00041	1.85082	0.66042	0.61737

Table S10. Overview of the Mulliken charges, spin densities, and isotropic fermi contact couplings for monoradical **MCN**-1 with the radical located on the non-substitutes phenalenyl unit (gray; M06-2X/cc-pVTZ).

Mulliken charges and spin densities

	1	2
1 C	-0.13721	0.089152
2 C	-0.155842	-0.055074
3 C	0.114493	0.117705
4 C	-0.158267	-0.094063
5 C	0.041014	0.064967
6 C	0.03979	-0.039353
7 C	-0.162372	-0.081904
8 C	-0.129824	0.092838
9 C	0.06615	-0.168357
10 C	0.036558	0.240451
11 C	-0.052232	-0.024436
12 C	-0.017422	0.018582
13 C	-0.116178	-0.019965
14 C	-0.138898	0.01671
15 C	0.129762	0.015866
16 C	-0.100796	-0.006143
17 C	0.147235	0.008399
18 C	-0.356761	-0.004428
19 C	-0.203985	0.44828
20 C	0.071967	-0.192603
21 C	-0.033114	0.083289
22 C	0.042256	-0.109731
23 C	0.085469	-0.159794
24 C	-0.128093	0.22904
25 C	-0.190683	-0.132603
26 C	0.138671	0.222803
27 C	-0.102171	0.34377
28 C	-0.147605	-0.179623
29 C	-0.164945	0.351662
30 C	-0.143087	-0.016662
31 C	-0.170602	0.010985
32 C	-0.152095	-0.011744
33 C	0.137232	0.009813
34 C	-0.176478	-0.006881
35 C	-0.115362	0.004539
36 C	-0.115299	-0.005598
37 C	-0.460975	-0.016033
38 C	0.039066	-0.000028

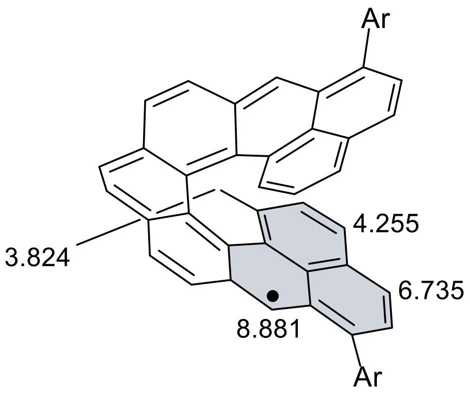

39	C	-0.167379	-0.000088
40	C	0.095563	0.000067
41	C	-0.164235	-0.000236
42	C	0.103358	0.000124
43	C	-0.183594	-0.000147
44	C	-0.37803	-0.000002
45	C	-0.376684	-0.00001
46	C	0.051009	-0.036993
47	C	-0.221115	0.021135
48	C	0.108145	-0.010611
49	C	-0.168734	0.019447
50	C	0.094604	-0.009618
51	C	-0.163804	0.022364
52	C	-0.3786	0.000395
53	C	-0.378327	0.000357
54	H	0.098421	-0.005514
55	H	0.097649	0.002364
56	H	0.098061	0.004519
57	H	0.098102	-0.004042
58	H	0.103454	0.001145
59	H	0.098111	-0.000641
60	H	0.173391	0.00049
61	H	0.148492	-0.029706
62	H	0.134745	-0.018547
63	H	0.115446	0.006407
64	H	0.124104	0.007067
65	H	0.112477	-0.024883
66	H	0.155407	0.002144
67	H	0.122062	-0.001684
68	H	0.132936	0.000676
69	H	0.110706	0.001058
70	H	0.126468	-0.000325
71	H	0.150326	0.001325
72	H	0.159193	0.009631
73	H	0.132522	0.004696
74	H	0.135031	0.000155
75	H	0.104748	0.000015
76	H	0.138627	-0.000084
77	H	0.104429	-0.000001
78	H	0.121663	0.000002
79	H	0.1303	0.000003
80	H	0.106107	0
81	H	0.119066	0.000001
82	H	0.128522	0.000003

83 H	0.149554	-0.004889
84 H	0.105427	-0.001317
85 H	0.130975	-0.000561
86 H	0.123925	-0.000244
87 H	0.106217	0.0001
88 H	0.129578	-0.000188
89 H	0.12274	-0.000133
90 H	0.10428	0.000062
91 H	0.128851	-0.000183
92 H	0.186342	0.001065

Sum of Mulliken charges = –0.00000 1.00000

Isotropic Fermi Contact couplings

Atom	a.u.	MegaHertz	Gauss	10^{-4} cm^{-1}
1 C(13)	0.01216	13.6688	4.87737	4.55942
2 C(13)	-0.00954	-10.72047	-3.82533	-3.57596
3 C(13)	0.02063	23.19672	8.27717	7.73759
4 C(13)	-0.01962	-22.05128	-7.86845	-7.35552
5 C(13)	0.0094	10.56873	3.77119	3.52535
6 C(13)	-0.00723	-8.12863	-2.9005	-2.71142
7 C(13)	-0.01319	-14.83096	-5.29206	-4.94708
8 C(13)	0.01538	17.28927	6.16924	5.76708
9 C(13)	-0.03247	-36.49918	-13.02382	-12.17482
10 C(13)	0.03977	44.70923	15.95337	14.91339
11 C(13)	-0.00418	-4.69459	-1.67515	-1.56595
12 C(13)	0.0033	3.70825	1.3232	1.23694
13 C(13)	-0.00281	-3.15739	-1.12663	-1.05319
14 C(13)	0.003	3.37147	1.20302	1.1246
15 C(13)	0.00206	2.31376	0.82561	0.77179
16 C(13)	-0.00108	-1.21275	-0.43274	-0.40453
17 C(13)	0.00063	0.7068	0.2522	0.23576
18 C(13)	-0.00059	-0.6617	-0.23611	-0.22072
19 C(13)	0.06193	69.62618	24.84435	23.22479
20 C(13)	-0.03565	-40.07757	-14.30068	-13.36844
21 C(13)	0.01888	21.22059	7.57203	7.07843
22 C(13)	-0.02667	-29.98087	-10.69792	-10.00054
23 C(13)	-0.02513	-28.24999	-10.0803	-9.42318
24 C(13)	0.02981	33.51203	11.95793	11.17841
25 C(13)	-0.02166	-24.34566	-8.68714	-8.12084
26 C(13)	0.03838	43.14659	15.39578	14.39215
27 C(13)	0.05336	59.98761	21.40507	20.00971
28 C(13)	-0.0304	-34.17366	-12.19401	-11.39911
29 C(13)	0.04656	52.34381	18.67757	17.46002

30	C(13)	-0.00085	-0.95795	-0.34182	-0.31954
31	C(13)	0.00104	1.17353	0.41874	0.39145
32	C(13)	-0.00103	-1.15798	-0.4132	-0.38626
33	C(13)	0.00109	1.2287	0.43843	0.40985
34	C(13)	-0.0002	-0.22571	-0.08054	-0.07529
35	C(13)	0.00093	1.04142	0.3716	0.34738
36	C(13)	-0.00062	-0.69889	-0.24938	-0.23313
37	C(13)	-0.00749	-8.42154	-3.00502	-2.80912
38	C(13)	0.00015	0.16347	0.05833	0.05453
39	C(13)	-0.00011	-0.12028	-0.04292	-0.04012
40	C(13)	0.00001	0.01424	0.00508	0.00475
41	C(13)	-0.00003	-0.03076	-0.01098	-0.01026
42	C(13)	0.00001	0.01316	0.0047	0.00439
43	C(13)	-0.00012	-0.13999	-0.04995	-0.0467
44	C(13)	0	-0.00388	-0.00139	-0.0013
45	C(13)	0	-0.00307	-0.0011	-0.00102
46	C(13)	-0.01161	-13.05063	-4.65679	-4.35322
47	C(13)	0.00877	9.85447	3.51632	3.2871
48	C(13)	-0.00128	-1.43348	-0.5115	-0.47816
49	C(13)	0.00251	2.81719	1.00524	0.93971
50	C(13)	-0.00057	-0.6429	-0.2294	-0.21445
51	C(13)	0.01178	13.24277	4.72535	4.41731
52	C(13)	0.00044	0.49914	0.1781	0.16649
53	C(13)	0.00033	0.37478	0.13373	0.12501
54	H(1)	-0.00111	-4.9449	-1.76446	-1.64944
55	H(1)	0.0004	1.76889	0.63118	0.59004
56	H(1)	0.00091	4.05882	1.44829	1.35388
57	H(1)	-0.00106	-4.75626	-1.69715	-1.58652
58	H(1)	0.00021	0.95102	0.33935	0.31723
59	H(1)	-0.00017	-0.7752	-0.27661	-0.25858
60	H(1)	0.0002	0.90697	0.32363	0.30253
61	H(1)	-0.00557	-24.88817	-8.88072	-8.3018
62	H(1)	-0.00267	-11.9237	-4.25467	-3.97732
63	H(1)	0.00122	5.44174	1.94175	1.81517
64	H(1)	0.0012	5.3507	1.90926	1.7848
65	H(1)	-0.00422	-18.87615	-6.73548	-6.29641
66	H(1)	0.00033	1.49391	0.53306	0.49831
67	H(1)	-0.00005	-0.22242	-0.07937	-0.07419
68	H(1)	0.0001	0.44803	0.15987	0.14945
69	H(1)	0.00015	0.68107	0.24302	0.22718
70	H(1)	-0.00004	-0.2005	-0.07154	-0.06688
71	H(1)	0.00052	2.33492	0.83316	0.77885
72	H(1)	0.00446	19.92464	7.10961	6.64614
73	H(1)	0.00221	9.88674	3.52783	3.29786

74	H(1)	0	0.01637	0.00584	0.00546
75	H(1)	0	0.01299	0.00464	0.00433
76	H(1)	0	0.01815	0.00648	0.00606
77	H(1)	0	0.00073	0.00026	0.00024
78	H(1)	0	0.00535	0.00191	0.00179
79	H(1)	0	0.0077	0.00275	0.00257
80	H(1)	0	0.00028	0.0001	0.00009
81	H(1)	0	0.00417	0.00149	0.00139
82	H(1)	0	0.00767	0.00274	0.00256
83	H(1)	-0.00025	-1.10573	-0.39455	-0.36883
84	H(1)	-0.00022	-0.99353	-0.35452	-0.33141
85	H(1)	-0.00034	-1.5051	-0.53706	-0.50205
86	H(1)	-0.00011	-0.49283	-0.17585	-0.16439
87	H(1)	-0.00001	-0.03368	-0.01202	-0.01123
88	H(1)	-0.0001	-0.45889	-0.16374	-0.15307
89	H(1)	-0.00009	-0.40705	-0.14525	-0.13578
90	H(1)	0.00001	0.05362	0.01913	0.01789
91	H(1)	-0.00012	-0.53734	-0.19174	-0.17924
92	H(1)	0.00052	2.33753	0.83409	0.77972

Table S11. Overview of the Mulliken charges, spin densities and isotropic fermi contact couplings for triplet **MCN** (M06-2X/cc-pVTZ).

Mulliken charges and spin densities

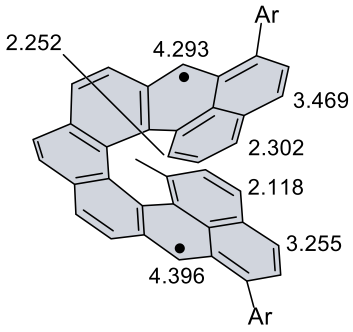

		1	2
1	C	-0.145472	0.034206
2	C	-0.152177	0.023164
3	C	0.112691	0.067562
4	C	-0.149308	-0.025634
5	C	0.026273	-0.013064
6	C	0.041655	0.058044
7	C	-0.161348	-0.067155
8	C	-0.12661	0.067971
9	C	0.048362	-0.162075
10	C	0.040124	0.243666
11	C	-0.040076	0.213522
12	C	0.035819	-0.153241
13	C	-0.126163	0.056029
14	C	-0.148584	-0.058732
15	C	0.193081	-0.10813
16	C	-0.011248	0.072154
17	C	0.061532	-0.186604
18	C	-0.207636	0.435892

19	C	-0.196373	0.440591
20	C	0.052199	-0.183857
21	C	-0.0129	0.080758
22	C	0.036207	-0.104125
23	C	0.071752	-0.151013
24	C	-0.11172	0.226106
25	C	-0.19608	-0.127313
26	C	0.131951	0.215918
27	C	-0.094985	0.336985
28	C	-0.151077	-0.172583
29	C	-0.149481	0.338251
30	C	-0.138606	0.246744
31	C	-0.167729	-0.133138
32	C	-0.160051	0.246967
33	C	0.110173	-0.156152
34	C	-0.155837	0.360547
35	C	-0.14303	-0.182812
36	C	-0.101874	0.35614
37	C	-0.458978	-0.00999
38	C	0.050388	-0.038521
39	C	-0.163168	0.024551
40	C	0.097033	-0.011123
41	C	-0.167549	0.022057
42	C	0.105607	-0.011895
43	C	-0.220378	0.023236
44	C	-0.378757	0.000436
45	C	-0.377407	0.000377
46	C	0.054315	-0.037181
47	C	-0.222352	0.021438
48	C	0.107282	-0.010775
49	C	-0.167536	0.019853
50	C	0.094295	-0.009924
51	C	-0.164142	0.022485
52	C	-0.378735	0.000392
53	C	-0.37786	0.000357
54	H	0.097703	-0.002991
55	H	0.097801	-0.002334
56	H	0.098509	0.004044
57	H	0.098921	-0.002475
58	H	0.100323	-0.002051
59	H	0.099998	0.003588
60	H	0.147437	-0.028883
61	H	0.148533	-0.029686
62	H	0.126349	-0.019065

63	H	0.117492	0.005709
64	H	0.123368	0.00669
65	H	0.110056	-0.022837
66	H	0.161406	-0.017206
67	H	0.124827	0.003746
68	H	0.125552	-0.019399
69	H	0.107963	-0.023339
70	H	0.122893	0.007084
71	H	0.15084	-0.000089
72	H	0.158474	0.00852
73	H	0.135299	0.004536
74	H	0.127554	-0.000484
75	H	0.105725	-0.001496
76	H	0.150606	-0.004981
77	H	0.106278	0.0001
78	H	0.124129	-0.000272
79	H	0.129778	-0.000212
80	H	0.104677	0.000059
81	H	0.122266	-0.000146
82	H	0.128757	-0.000215
83	H	0.150049	-0.00494
84	H	0.105605	-0.001345
85	H	0.129468	-0.000381
86	H	0.124388	-0.000249
87	H	0.106246	0.0001
88	H	0.129515	-0.000189
89	H	0.122106	-0.000132
90	H	0.104675	0.000055
91	H	0.128921	-0.000194

Sum of Mulliken charges = –0.00000 2.00000

Isotropic Fermi Contact couplings

Atom		a.u.	MegaHertz	Gauss	10^{-4} cm^{-1}
1	C(13)	0.00317	1.7842	0.63665	0.59515
2	C(13)	0.00135	0.761	0.27154	0.25384
3	C(13)	0.01221	6.86113	2.44822	2.28863
4	C(13)	-0.0098	-5.50945	-1.96591	-1.83775
5	C(13)	-0.00729	-4.09847	-1.46244	-1.3671
6	C(13)	0.01019	5.72998	2.0446	1.91132
7	C(13)	-0.01038	-5.83279	-2.08129	-1.94561
8	C(13)	0.01211	6.80433	2.42795	2.26968
9	C(13)	-0.03105	-17.45132	-6.22707	-5.82113
10	C(13)	0.03919	22.03106	7.86123	7.34877

11	C(13)	0.03635	20.43448	7.29153	6.81621
12	C(13)	-0.02888	-16.23303	-5.79235	-5.41476
13	C(13)	0.01016	5.71212	2.03823	1.90536
14	C(13)	-0.0091	-5.11602	-1.82552	-1.70652
15	C(13)	-0.02428	-13.64745	-4.86975	-4.5523
16	C(13)	0.01441	8.09989	2.89024	2.70183
17	C(13)	-0.03497	-19.65879	-7.01474	-6.55747
18	C(13)	0.05956	33.47853	11.94597	11.16724
19	C(13)	0.06117	34.38182	12.26829	11.46854
20	C(13)	-0.03488	-19.60342	-6.99499	-6.539
21	C(13)	0.01837	10.32844	3.68544	3.4452
22	C(13)	-0.02557	-14.37027	-5.12767	-4.79341
23	C(13)	-0.02444	-13.73638	-4.90148	-4.58196
24	C(13)	0.02994	16.82899	6.005	5.61355
25	C(13)	-0.02131	-11.97643	-4.27349	-3.99491
26	C(13)	0.03919	22.02696	7.85977	7.3474
27	C(13)	0.0523	29.39919	10.49036	9.80651
28	C(13)	-0.02917	-16.3945	-5.84996	-5.46862
29	C(13)	0.04633	26.0434	9.29293	8.68714
30	C(13)	0.03587	20.16027	7.19369	6.72474
31	C(13)	-0.02187	-12.29121	-4.38581	-4.09991
32	C(13)	0.03288	18.48444	6.59571	6.16575
33	C(13)	-0.02588	-14.54618	-5.19044	-4.85208
34	C(13)	0.04908	27.58571	9.84327	9.2016
35	C(13)	-0.03126	-17.5714	-6.26991	-5.86119
36	C(13)	0.05563	31.26985	11.15786	10.4305
37	C(13)	-0.00741	-4.16681	-1.48682	-1.3899
38	C(13)	-0.01228	-6.90066	-2.46233	-2.30181
39	C(13)	0.01242	6.97987	2.49059	2.32824
40	C(13)	-0.00078	-0.43588	-0.15553	-0.14539
41	C(13)	0.00285	1.59925	0.57065	0.53345
42	C(13)	-0.00149	-0.83829	-0.29912	-0.27962
43	C(13)	0.00922	5.17992	1.84832	1.72783
44	C(13)	0.00048	0.27019	0.09641	0.09012
45	C(13)	0.00036	0.20013	0.07141	0.06676
46	C(13)	-0.01144	-6.43235	-2.29522	-2.1456
47	C(13)	0.00858	4.82159	1.72046	1.60831
48	C(13)	-0.00133	-0.7469	-0.26651	-0.24914
49	C(13)	0.00256	1.4378	0.51304	0.4796
50	C(13)	-0.00065	-0.36381	-0.12982	-0.12135
51	C(13)	0.0115	6.46296	2.30614	2.15581
52	C(13)	0.00044	0.24625	0.08787	0.08214
53	C(13)	0.00033	0.18727	0.06682	0.06247
54	H(1)	-0.00065	-1.45916	-0.52066	-0.48672

55	H(1)	-0.00053	-1.18499	-0.42284	-0.39527
56	H(1)	0.00079	1.75969	0.6279	0.58697
57	H(1)	-0.00075	-1.67032	-0.59601	-0.55716
58	H(1)	-0.00064	-1.4391	-0.51351	-0.48003
59	H(1)	0.00067	1.49413	0.53314	0.49839
60	H(1)	-0.00538	-12.0317	-4.29321	-4.01334
61	H(1)	-0.00551	-12.32136	-4.39657	-4.10996
62	H(1)	-0.00266	-5.93479	-2.11768	-1.97963
63	H(1)	0.0012	2.67452	0.95433	0.89212
64	H(1)	0.00113	2.53387	0.90415	0.84521
65	H(1)	-0.00408	-9.12337	-3.25545	-3.04323
66	H(1)	-0.00282	-6.31279	-2.25256	-2.10572
67	H(1)	0.00101	2.26361	0.80771	0.75506
68	H(1)	-0.00289	-6.45079	-2.3018	-2.15175
69	H(1)	-0.00435	-9.72261	-3.46927	-3.24311
70	H(1)	0.00121	2.711	0.96735	0.90429
71	H(1)	0.00046	1.03721	0.3701	0.34598
72	H(1)	0.00426	9.51555	3.39539	3.17405
73	H(1)	0.00218	4.88313	1.74242	1.62884
74	H(1)	-0.00037	-0.82979	-0.29609	-0.27679
75	H(1)	-0.00025	-0.56346	-0.20106	-0.18795
76	H(1)	-0.00028	-0.62057	-0.22144	-0.207
77	H(1)	-0.00001	-0.0193	-0.00689	-0.00644
78	H(1)	-0.00012	-0.27558	-0.09833	-0.09192
79	H(1)	-0.00012	-0.25902	-0.09243	-0.0864
80	H(1)	0.00001	0.02203	0.00786	0.00735
81	H(1)	-0.0001	-0.22059	-0.07871	-0.07358
82	H(1)	-0.00014	-0.3081	-0.10994	-0.10277
83	H(1)	-0.00025	-0.55588	-0.19835	-0.18542
84	H(1)	-0.00023	-0.508	-0.18127	-0.16945
85	H(1)	-0.00034	-0.7552	-0.26947	-0.25191
86	H(1)	-0.00011	-0.25314	-0.09033	-0.08444
87	H(1)	-0.00001	-0.01691	-0.00603	-0.00564
88	H(1)	-0.0001	-0.23069	-0.08232	-0.07695
89	H(1)	-0.00009	-0.19979	-0.07129	-0.06664
90	H(1)	0.00001	0.02286	0.00816	0.00763
91	H(1)	-0.00012	-0.2784	-0.09934	-0.09287

10. Cartesian Coordinates

The Cartesian coordinates are shown for geometries optimized at the B3LYP-D3/cc-pVTZ level (if not stated differently) of DFT in the gas phase. The unrestricted broken-symmetry approach was utilized to adjust for the diradicaloid nature of the open forms of **MNC**, which resulted in the lowest-energy configuration.

Table S12. Cartesian coordinates.

MNC-Ar (broken-symmetry unrestricted singlet)

	X	Y	Z
C	-0.618636	5.654294	-0.383734
C	0.749834	5.657988	-0.332949
C	1.46739	4.459057	-0.114916
C	0.752462	3.239937	0.049037
C	-0.626018	3.205281	-0.391901
C	-1.337131	4.436189	-0.418788
C	2.891379	4.442671	-0.044856
C	3.558993	3.275328	0.142493
C	2.852442	2.073662	0.464243
C	1.440747	2.127315	0.627473
C	-1.324409	2.016238	-0.776914
C	-2.738869	1.997526	-0.644749
C	-3.43642	3.235886	-0.484646
C	-2.76152	4.414192	-0.47191
C	-0.692099	0.818316	-1.293346
C	-1.390654	-0.417271	-1.227337
C	-2.770553	-0.453033	-0.835059
C	-3.425202	0.776484	-0.640768
C	3.513669	0.844562	0.54826
C	2.821396	-0.354691	0.803468
C	1.451742	-0.261418	1.225478
C	0.798698	1.005345	1.29105
C	0.749362	-1.44126	1.594792
C	-0.539498	-1.319847	2.146468
C	-1.060153	-0.075081	2.408731
C	-0.408414	1.102896	2.022389
C	3.422181	-1.636694	0.673195
C	2.659562	-2.780991	0.933876
C	1.36638	-2.697092	1.400244
C	0.573371	0.832411	-1.900758
C	1.205166	-0.343272	-2.284926
C	0.595976	-1.564564	-2.076164
C	-0.712471	-1.624794	-1.561722
C	-1.379847	-2.855325	-1.356604
C	-2.682393	-2.884995	-0.908957

Thermochemistry
Temperature 298.150 Kelvin. Pressure: 1.00000 Atm.
Zero-point correction = 0.735345 (Hartree/Particle)
Thermal correction to Energy = 0.777580
Thermal correction to Enthalpy = 0.778524
Thermal correction to Gibbs Free Energy = 0.657628
Sum of electronic and zero-point Energies = -2042.639001
Sum of electronic and thermal Energies = -2042.596765
Sum of electronic and thermal Enthalpies = -2042.595821
Sum of electronic and thermal Free Energies = -2042.716718

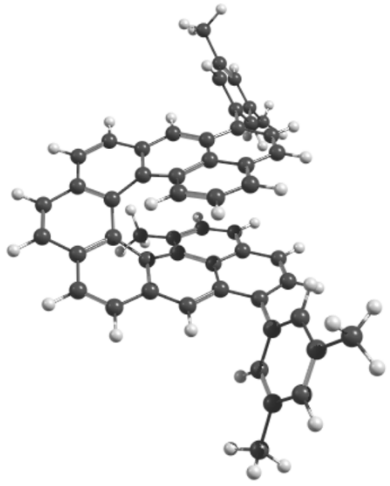

C	-3.414499	-1.712828	-0.683204
C	-1.007643	2.40538	2.487166
C	-4.837377	-1.835553	-0.285379
C	-5.179508	-2.548366	0.86442
C	-6.509099	-2.704442	1.251792
C	-7.504601	-2.14258	0.456419
C	-7.197687	-1.435207	-0.706072
C	-5.860986	-1.285677	-1.062266
C	-8.294775	-0.879221	-1.576637
C	-6.855697	-3.433233	2.52423
C	4.832158	-1.811615	0.248535
C	5.889296	-1.285686	0.995963
C	7.212516	-1.482843	0.613053
C	7.47093	-2.214053	-0.546417
C	6.440677	-2.752836	-1.313105
C	5.125642	-2.548874	-0.899242
C	8.345461	-0.951632	1.452602
C	6.735895	-3.507618	-2.58334
H	-1.168564	6.584359	-0.448526
H	1.300041	6.587148	-0.40833
H	3.428623	5.3691	-0.203196
H	4.63931	3.238464	0.085799
H	-4.517078	3.2157	-0.426873
H	-3.294451	5.35616	-0.446906
H	-4.485072	0.790641	-0.43638
H	4.570506	0.817371	0.330262
H	-1.091334	-2.213794	2.405141
H	-2.008499	0.008095	2.924612
H	3.123265	-3.750127	0.806224
H	0.811441	-3.594954	1.638503
H	1.075815	1.774026	-2.054391
H	2.193479	-0.297004	-2.721395
H	1.109357	-2.485523	-2.318229
H	-0.855063	-3.777359	-1.569612
H	-3.179536	-3.836128	-0.771167
H	-0.290741	3.221804	2.462117
H	-1.868681	2.703752	1.888253
H	-1.355748	2.290489	3.514851
H	-4.390917	-2.973517	1.472896
H	-8.542888	-2.260045	0.745381
H	-5.605405	-0.751539	-1.968325
H	-7.933687	-0.06294	-2.201938
H	-9.127075	-0.506954	-0.978226
H	-8.692407	-1.650345	-2.241304

H	-7.854414	-3.867354	2.476433
H	-6.146012	-4.235033	2.729732
H	-6.835028	-2.753276	3.379783
H	5.67043	-0.731028	1.899378
H	8.49832	-2.368685	-0.856095
H	4.310303	-2.955497	-1.48472
H	9.180728	-0.624594	0.832362
H	8.027024	-0.108997	2.066054
H	8.724717	-1.723392	2.127225
H	5.992991	-4.284259	-2.766788
H	7.718397	-3.978487	-2.548865
H	6.724852	-2.836472	-3.445999

MNC-Ar (unrestricted triplet)

	X	Y	Z
C	-0.621373	5.639492	-0.381131
C	0.743089	5.644664	-0.35262
C	1.464076	4.44249	-0.142831
C	0.756615	3.224578	0.031471
C	-0.630315	3.187492	-0.377601
C	-1.338414	4.416245	-0.400017
C	2.889617	4.434045	-0.101279
C	3.56795	3.27133	0.070631
C	2.874932	2.066443	0.407155
C	1.467655	2.108272	0.591447
C	-1.347601	1.992004	-0.735478
C	-2.75781	1.986696	-0.582001
C	-3.445598	3.228928	-0.413961
C	-2.764136	4.402249	-0.423833
C	-0.736108	0.790926	-1.262357
C	-1.450728	-0.436137	-1.202037
C	-2.82968	-0.458559	-0.804091
C	-3.463	0.77381	-0.584956
C	3.555131	0.845909	0.501869
C	2.880546	-0.35562	0.776024
C	1.511609	-0.273519	1.204107
C	0.846806	0.986711	1.269763
C	0.820442	-1.459212	1.577041
C	-0.468875	-1.348805	2.130882
C	-0.9984	-0.108039	2.39707
C	-0.356515	1.074928	2.011818
C	3.497879	-1.63424	0.657726
C	2.745869	-2.782417	0.920795
C	1.448579	-2.708516	1.382229
C	0.529078	0.790432	-1.875815

Thermochemistry
Temperature 298.150 Kelvin. Pressure: 1.00000 Atm.
Zero-point correction = 0.735479 (Hartree/Particle)
Thermal correction to Energy = 0.777722
Thermal correction to Enthalpy = 0.778666
Thermal correction to Gibbs Free Energy = 0.656699
Sum of electronic and zero-point Energies = -2042.637399
Sum of electronic and thermal Energies = -2042.595156
Sum of electronic and thermal Enthalpies = -2042.594212
Sum of electronic and thermal Free Energies = -2042.716179

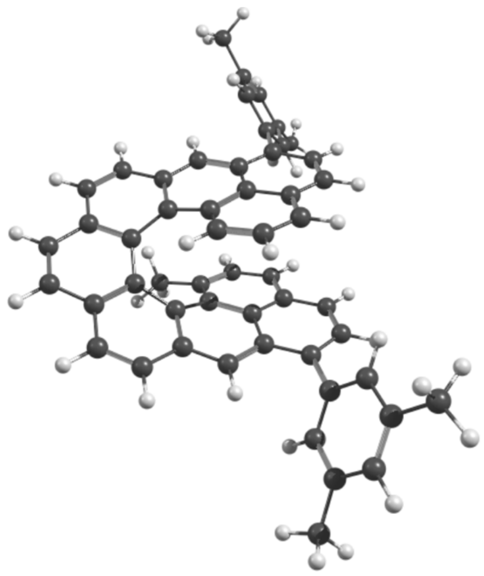

C	1.143897	-0.391373	-2.266419
C	0.518588	-1.605935	-2.063237
C	-0.790359	-1.650917	-1.548087
C	-1.475281	-2.872758	-1.354811
C	-2.781128	-2.890147	-0.911253
C	-3.495765	-1.712757	-0.672072
C	-0.946967	2.372617	2.503079
C	-4.921488	-1.817754	-0.280657
C	-5.281668	-2.551488	0.850257
C	-6.61566	-2.692373	1.228795
C	-7.597318	-2.093286	0.443806
C	-7.272539	-1.363715	-0.700216
C	-5.932122	-1.230337	-1.047689
C	-8.356259	-0.766379	-1.560087
C	-6.980379	-3.445492	2.481872
C	4.911517	-1.795873	0.241441
C	5.957286	-1.238639	0.982777
C	7.285046	-1.424002	0.610683
C	7.560417	-2.175345	-0.532073
C	6.54231	-2.745129	-1.292299
C	5.222146	-2.552206	-0.889316
C	8.406009	-0.858658	1.444018
C	6.854996	-3.521792	-2.545064
H	-1.175579	6.567377	-0.439579
H	1.293502	6.572702	-0.439024
H	3.41757	5.3639	-0.270191
H	4.647214	3.241365	-0.006106
H	-4.525068	3.215122	-0.336114
H	-3.29037	5.347781	-0.393855
H	-4.520905	0.802015	-0.372411
H	4.610722	0.831004	0.277233
H	-1.012487	-2.247429	2.39073
H	-1.943903	-0.032939	2.919494
H	3.218104	-3.748295	0.799794
H	0.901272	-3.611442	1.619368
H	1.039783	1.726194	-2.035152
H	2.131047	-0.355841	-2.706586
H	1.018314	-2.532296	-2.313112
H	-0.963067	-3.800053	-1.575838
H	-3.291285	-3.836396	-0.788082
H	-0.234338	3.191859	2.46217
H	-1.828594	2.671891	1.935664
H	-1.260151	2.248753	3.54103
H	-4.503811	-3.005786	1.451419

H	-8.638798	-2.198467	0.725962
H	-5.663269	-0.679764	-1.939923
H	-7.97285	0.042224	-2.181975
H	-9.172239	-0.371486	-0.953591
H	-8.783286	-1.519524	-2.227081
H	-7.989976	-3.852278	2.423797
H	-6.291505	-4.270533	2.665139
H	-6.941482	-2.789317	3.355172
H	5.725734	-0.669067	1.873625
H	8.591607	-2.321047	-0.833354
H	4.416254	-2.98294	-1.470596
H	9.232917	-0.518329	0.819642
H	8.068726	-0.017721	2.049619
H	8.803886	-1.614944	2.12538
H	6.128084	-4.317031	-2.712286
H	7.846627	-3.972025	-2.498745
H	6.832014	-2.870304	-3.422429

c-MNC-Ar

	X	Y	Z
C	0.469333	5.493453	0.163705
C	-0.886453	5.490975	0.383673
C	-1.608582	4.286649	0.324144
C	-0.904382	3.082576	0.084105
C	0.499763	3.071353	0.072481
C	1.181581	4.287758	-0.006909
C	-3.029885	4.234774	0.513214
C	-3.709507	3.066939	0.432735
C	-3.050117	1.843557	0.068039
C	-1.644867	1.865206	-0.165569
C	1.230124	1.726056	0.056009
C	2.720426	1.880539	-0.234305
C	3.264905	3.166316	-0.586255
C	2.576701	4.30535	-0.392265
C	1.106392	0.948345	1.385515
C	1.995505	-0.14252	1.598995
C	3.181667	-0.291892	0.807992
C	3.565722	0.853811	0.019607
C	-3.75224	0.659228	-0.103542
C	-3.114996	-0.517202	-0.515168
C	-1.705643	-0.472311	-0.793513
C	-0.995213	0.735284	-0.658197
C	-1.022354	-1.676008	-1.160357
C	0.401221	-1.613582	-1.421663

Thermochemistry
Temperature 298.150 Kelvin. Pressure: 1.00000 Atm.
Zero-point correction = 0.739007 (Hartree/Particle)
Thermal correction to Energy = 0.780407
Thermal correction to Enthalpy = 0.781351
Thermal correction to Gibbs Free Energy = 0.663155
Sum of electronic and zero-point Energies = -2042.631467
Sum of electronic and thermal Energies = -2042.590067
Sum of electronic and thermal Enthalpies = -2042.589123
Sum of electronic and thermal Free Energies = -2042.707319

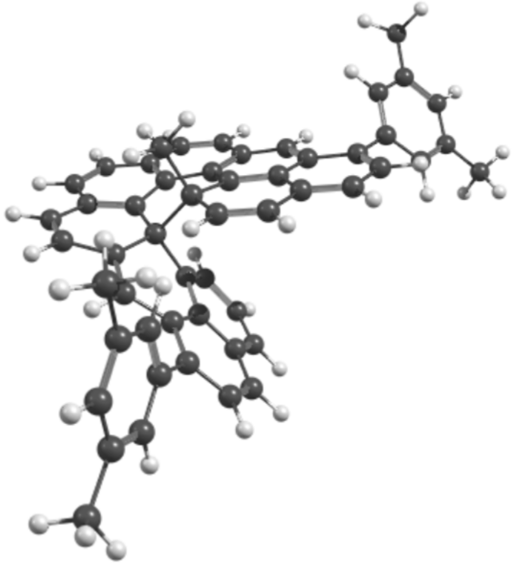

C	1.088741	-0.474049	-1.359764
C	0.466135	0.861789	-1.082161
C	-3.816982	-1.765732	-0.65183
C	-3.108833	-2.894116	-0.986345
C	-1.723524	-2.854866	-1.233402
C	0.117081	1.176218	2.317
C	-0.099966	0.296823	3.394262
C	0.671649	-0.823393	3.541412
C	1.747247	-1.058859	2.657139
C	2.599996	-2.178981	2.802182
C	3.678472	-2.352237	1.978718
C	4.005647	-1.404204	0.981338
C	0.533388	1.626761	-2.437207
C	5.219463	-1.624187	0.161644
C	5.179305	-1.520098	-1.231088
C	6.309586	-1.751553	-2.009686
C	7.502188	-2.085224	-1.370696
C	7.579179	-2.197435	0.016803
C	6.429706	-1.97159	0.768678
C	8.888738	-2.517578	0.689834
C	6.233632	-1.680176	-3.512858
C	-5.282882	-1.868918	-0.440031
C	-6.178345	-1.123163	-1.213271
C	-7.553087	-1.251157	-1.049823
C	-8.031926	-2.138562	-0.08435
C	-7.168038	-2.894329	0.702152
C	-5.793828	-2.75198	0.50972
C	-8.511345	-0.476723	-1.917367
C	-7.69689	-3.821688	1.765517
H	1.012512	6.429032	0.121654
H	-1.415328	6.417913	0.56526
H	-3.549802	5.156793	0.741219
H	-4.777817	3.039927	0.605873
H	4.303498	3.20104	-0.891962
H	3.046979	5.266156	-0.557358
H	4.612537	0.962154	-0.231521
H	-4.814173	0.6511	0.092041
H	0.905159	-2.536174	-1.683175
H	2.136652	-0.464769	-1.612402
H	-3.636489	-3.833527	-1.084838
H	-1.204014	-3.767869	-1.49553
H	-0.531723	2.030662	2.221898
H	-0.902008	0.505532	4.089369
H	0.485699	-1.525956	4.343646

H	2.382953	-2.903393	3.576849
H	4.304933	-3.227191	2.08733
H	0.067225	2.607155	-2.374937
H	1.571943	1.755551	-2.742799
H	0.01841	1.047112	-3.201278
H	4.2442	-1.273328	-1.714874
H	8.390398	-2.263164	-1.966313
H	6.477406	-2.042981	1.847953
H	8.733961	-2.997798	1.655952
H	9.467965	-1.60756	0.865927
H	9.500927	-3.178536	0.07568
H	7.177093	-1.344421	-3.943982
H	6.012176	-2.662309	-3.938278
H	5.448503	-0.998592	-3.840197
H	-5.789338	-0.446786	-1.963499
H	-9.102246	-2.241339	0.054418
H	-5.105307	-3.326012	1.117186
H	-9.362187	-0.1113	-1.34069
H	-8.025096	0.379275	-2.38454
H	-8.909206	-1.106516	-2.717035
H	-7.668175	-3.343641	2.747979
H	-7.099631	-4.731791	1.831589
H	-8.730265	-4.107118	1.569433

c-MNC'-Ar

	X	Y	Z
C	-0.872459	5.576290	-0.19372
C	0.500998	5.575886	-0.177459
C	1.231102	4.369392	-0.191072
C	0.544451	3.154443	-0.135792
C	-0.85389	3.157456	-0.26687
C	-1.582561	4.365389	-0.163674
C	2.670032	4.376075	-0.357595
C	3.373243	3.231076	-0.420493
C	2.778975	1.946657	-0.155268
C	1.272521	1.814119	0.023478
C	-1.570409	1.918345	-0.437949
C	-2.979558	1.877397	-0.252988
C	-3.6715	3.117219	-0.031699
C	-3.011472	4.3012	-0.042905
C	-0.877318	0.779688	-0.826488

Thermochemistry
Temperature 298.150 Kelvin. Pressure: 1.00000 Atm.
Zero-point correction = 0.738999 (Hartree/Particle)
Thermal correction to Energy = 0.780356
Thermal correction to Enthalpy = 0.781300
Thermal correction to Gibbs Free Energy = 0.663131
Sum of electronic and zero-point Energies = -2042.624495
Sum of electronic and thermal Energies = -2042.583137
Sum of electronic and thermal Enthalpies = -2042.582193
Sum of electronic and thermal Free Energies = -2042.700363

C	-1.544785	-0.450327	-0.927251
C	-2.953877	-0.524761	-0.657059
C	-3.63808	0.657263	-0.339739
C	3.560084	0.872689	0.099909
C	3.035797	-0.318026	0.72541
C	1.803081	-0.169271	1.447323
C	1.037997	1.039574	1.347683
C	1.409463	-1.198024	2.339444
C	0.31796	-0.964485	3.202483
C	-0.253449	0.272176	3.240285
C	0.110873	1.313987	2.345902
C	3.763373	-1.50683	0.810256
C	3.273341	-2.558753	1.615552
C	2.139715	-2.407353	2.366794
C	0.592706	0.935896	-1.147695
C	1.239431	-0.343999	-1.559039
C	0.586033	-1.506711	-1.618815
C	-0.817931	-1.632517	-1.277141
C	-1.473671	-2.839182	-1.294027
C	-2.850988	-2.92046	-1.009003
C	-3.603177	-1.806994	-0.721014
C	-0.531976	2.65031	2.645238
C	-5.061483	-1.960901	-0.487696
C	-5.524447	-2.799307	0.52476
C	-6.889438	-2.988432	0.741859
C	-7.792573	-2.326058	-0.083629
C	-7.36175	-1.485969	-1.112103
C	-5.995492	-1.30931	-1.299425
C	-8.36074	-0.815025	-2.01905
C	-7.36701	-3.865072	1.870521
C	5.039735	-1.711151	0.085299
C	6.153413	-2.225792	0.755976
C	7.359466	-2.445753	0.097198
C	7.439137	-2.155874	-1.264341
C	6.346135	-1.652675	-1.96661
C	5.156367	-1.431605	-1.27826
C	8.565861	-2.949443	0.846449
C	6.434988	-1.388453	-3.447429
H	-1.417112	6.508767	-0.033264
H	1.043679	6.512332	-0.205671
H	3.164782	5.331656	-0.474471
H	4.444491	3.257243	-0.578986
H	-4.745266	3.089537	0.103651
H	-3.555578	5.229684	0.077106

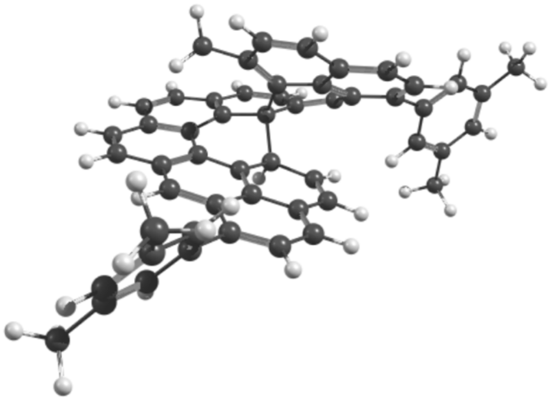

H	-4.70323	0.62665	-0.164483
H	4.628999	0.943275	-0.049344
H	-0.011568	-1.750111	3.870125
H	-1.02511	0.482663	3.970317
H	3.818021	-3.492735	1.639524
H	1.794521	-3.210928	3.004912
H	0.66788	1.608746	-2.014736
H	2.269895	-0.287434	-1.870472
H	1.103749	-2.400755	-1.944498
H	-0.928875	-3.740067	-1.54642
H	-3.341497	-3.883882	-1.053349
H	0.027005	3.491891	2.253271
H	-1.552588	2.71337	2.266317
H	-0.585483	2.766703	3.728694
H	-4.805776	-3.300567	1.161081
H	-8.856074	-2.465922	0.073843
H	-5.643091	-0.669518	-2.098259
H	-7.916879	0.027536	-2.548685
H	-9.222056	-0.448654	-1.458744
H	-8.737038	-1.514597	-2.769646
H	-8.394421	-4.19353	1.714813
H	-6.739734	-4.750809	1.977881
H	-7.333426	-3.327686	2.821646
H	6.079773	-2.435543	1.815585
H	8.372552	-2.327216	-1.788492
H	4.298075	-1.052263	-1.814751
H	9.188482	-3.585649	0.216729
H	9.188571	-2.117426	1.185023
H	8.277817	-3.521524	1.728297
H	5.752512	-0.594879	-3.752035
H	6.171986	-2.282407	-4.018676
H	7.444533	-1.100024	-3.741048

MNC without Ar (broken-symmetry unrestricted singlet)

	X	Y	Z
C	-4.688282	-0.794891	-0.204784
C	-4.724291	0.552274	-0.442229
C	-3.541076	1.324614	-0.39446
C	-2.301236	0.689467	-0.1011
C	-2.236846	-0.752565	-0.235916
C	-3.453662	-1.477057	-0.100897
C	-3.567879	2.731907	-0.619856
C	-2.42197	3.45808	-0.587867
C	-1.196319	2.867296	-0.147797

Thermochemistry
Temperature 298.150 Kelvin. Pressure: 1.00000 Atm.
Zero-point correction = 0.45074 (Hartree/Particle)
Thermal correction to Energy = 0.489970
Thermal correction to Enthalpy = 0.490915
Thermal correction to Gibbs Free Energy = 0.413620
Sum of electronic and zero-point Energies = -1423.288752
Sum of electronic and thermal Energies = -1423.263828
Sum of electronic and thermal Enthalpies = -1423.262883
Sum of electronic and thermal Free Energies = -1423.340178

C	-1.202867	1.514118	0.302216
C	-1.035514	-1.49684	-0.474346
C	-0.994693	-2.858256	-0.056115
C	-2.219856	-3.526793	0.253825
C	-3.407941	-2.880727	0.141656
C	0.148237	-0.965586	-1.125046
C	1.392338	-1.623399	-0.936886
C	1.447345	-2.887695	-0.269768
C	0.235877	-3.514546	0.066427
C	0.010809	3.568964	-0.209361
C	1.227019	2.96982	0.164895
C	1.185321	1.719237	0.860893
C	-0.057512	1.053977	1.072361
C	2.396131	1.173204	1.369988
C	2.323237	0.021204	2.177662
C	1.098378	-0.482389	2.545564
C	-0.107276	0.026988	2.042927
C	2.471149	3.584929	-0.068651
C	3.653803	2.992454	0.359345
C	3.624148	1.809487	1.074268
C	0.116632	0.144955	-1.982177
C	1.284555	0.68424	-2.50885
C	2.514411	0.133408	-2.208453
C	2.594013	-1.043299	-1.438171
C	3.830134	-1.675429	-1.157528
C	3.873962	-2.868183	-0.459073
C	2.701243	-3.485542	-0.037618
C	-1.383289	-0.504748	2.643232
H	-5.604941	-1.367088	-0.1427
H	-5.666506	1.054641	-0.619686
H	-4.511997	3.197515	-0.87229
H	-2.420593	4.505071	-0.862548
H	-2.182052	-4.572876	0.529141
H	-4.340926	-3.41055	0.28545
H	0.253526	-4.526866	0.45097
H	0.015872	4.575522	-0.608918
H	3.235736	-0.426246	2.548714
H	1.052124	-1.302824	3.250778
H	4.600544	3.4728	0.150444
H	4.541712	1.362013	1.433197
H	-0.829353	0.601443	-2.225609
H	1.224127	1.559023	-3.141714
H	3.424982	0.585197	-2.578633
H	4.742972	-1.214078	-1.510868

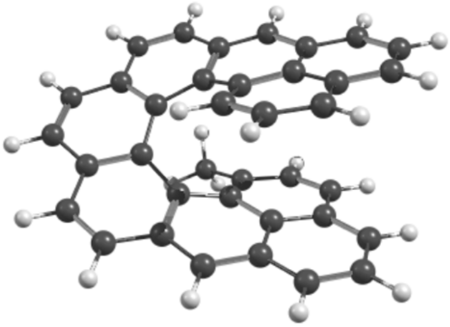

H	4.827634	-3.3384	-0.258253
H	-2.225805	0.161166	2.47655
H	-1.65333	-1.484016	2.246751
H	-1.249499	-0.619746	3.719974
H	2.740927	-4.440922	0.469474
H	2.495452	4.533135	-0.590075

MNC without Ar, transition state (broken-symmetry unrestricted singlet)

	X	Y	Z
C	4.433196	-1.542538	0.301679
C	4.715642	-0.239429	0.602888
C	3.70307	0.744113	0.516213
C	2.397171	0.355993	0.154096
C	2.060358	-1.023416	0.119219
C	3.101747	-1.957409	0.051171
C	3.965291	2.122905	0.814124
C	2.981833	3.051701	0.762234
C	1.674777	2.720304	0.268154
C	1.430164	1.389216	-0.174972
C	0.652466	-1.496005	0.06123
C	0.433655	-2.858472	-0.409507
C	1.570621	-3.697287	-0.6922
C	2.821436	-3.307915	-0.376524
C	-0.349449	-1.006211	1.011813
C	-1.673709	-1.518023	0.904128
C	-1.944906	-2.672467	0.105593
C	-0.837158	-3.373157	-0.451037
C	0.656865	3.667118	0.187936
C	-0.609919	3.323283	-0.292028
C	-0.803637	2.025686	-0.866302
C	0.262478	1.103764	-0.913029
C	-2.100797	1.66044	-1.349432
C	-2.274637	0.34697	-1.905708
C	-1.223421	-0.488217	-2.069556
C	0.107839	-0.15303	-1.64842
C	-1.708943	4.224636	-0.243443
C	-2.944822	3.844727	-0.694405
C	-3.145673	2.560716	-1.235561
C	-0.105565	-0.008131	1.953933
C	-1.153182	0.581693	2.672816
C	-2.452526	0.183127	2.479866
C	-2.741818	-0.892771	1.608842
C	-4.058834	-1.365507	1.411808
C	-4.307141	-2.462378	0.612162

Thermochemistry
Temperature 298.150 Kelvin. Pressure: 1.00000 Atm.
Zero-point correction = 0.465557 (Hartree/Particle)
Thermal correction to Energy = 0.489796
Thermal correction to Enthalpy = 0.490740
Thermal correction to Gibbs Free Energy = 0.414980
Sum of electronic and zero-point Energies = -1423.262267
Sum of electronic and thermal Energies = -1423.238028
Sum of electronic and thermal Enthalpies = -1423.237084
Sum of electronic and thermal Free Energies = -1423.312844

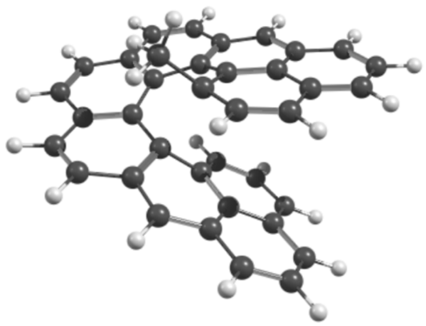

C	-3.256898	-3.13234	-0.014286
C	1.18126	-0.569461	-2.636005
H	5.223038	-2.28211	0.270034
H	5.721012	0.060996	0.868384
H	4.963696	2.397486	1.130087
H	3.174228	4.074746	1.059078
H	1.383098	-4.6953	-1.067844
H	3.655693	-3.987257	-0.495879
H	-0.995612	-4.372171	-0.83861
H	0.840906	4.677754	0.531268
H	-3.265351	0.050454	-2.225033
H	-1.362807	-1.441118	-2.563603
H	-3.778526	4.531479	-0.6328
H	-4.132434	2.26862	-1.57127
H	0.90248	0.32174	2.141167
H	-0.926902	1.372841	3.375107
H	-3.264049	0.665919	3.008445
H	-4.873661	-0.861621	1.915681
H	-5.32111	-2.818392	0.485759
H	2.190506	-0.387939	-2.281012
H	1.083492	-1.626445	-2.884446
H	1.050094	0.002697	-3.558251
H	-3.454025	-4.016054	-0.607614
H	-1.554762	5.210287	0.17639

c-MNC

	X	Y	Z
C	3.079382	-3.403280	0.12266
C	3.931091	-2.455683	0.635268
C	3.54776	-1.103813	0.660887
C	2.254144	-0.753705	0.20534
C	1.318917	-1.752405	-0.112981
C	1.768446	-3.065189	-0.273561
C	4.420078	-0.069982	1.138836
C	4.045434	1.230476	1.127571
C	2.789981	1.641329	0.562591
C	1.911937	0.642445	0.043369
C	-0.136993	-1.354077	-0.372876
C	-0.948665	-2.496584	-0.982441
C	-0.298106	-3.715482	-1.388686
C	0.94173	-4.026748	-0.971665
C	-0.877657	-0.87544	0.896803
C	-2.2968	-0.799283	0.840646
C	-3.028916	-1.490375	-0.174714

Thermochemistry
Temperature 298.150 Kelvin. Pressure: 1.00000 Atm.
Zero-point correction = 0.468762 (Hartree/Particle)
Thermal correction to Energy = 0.492853
Thermal correction to Enthalpy = 0.493797
Thermal correction to Gibbs Free Energy = 0.418355
Sum of electronic and zero-point Energies = -1423.279829
Sum of electronic and thermal Energies = -1423.255738
Sum of electronic and thermal Enthalpies = -1423.254794
Sum of electronic and thermal Free Energies = -1423.330235

C	-2.301307	-2.437772	-0.979888
C	2.428245	2.977374	0.467831
C	1.224334	3.360434	-0.131368
C	0.382101	2.356106	-0.711042
C	0.757708	1.002927	-0.6503
C	-0.854813	2.751642	-1.314733
C	-1.710994	1.715879	-1.859355
C	-1.36608	0.42833	-1.856256
C	-0.038773	-0.080751	-1.371349
C	0.8185	4.722804	-0.198757
C	-0.363708	5.070273	-0.788252
C	-1.206556	4.080793	-1.341693
C	-0.24707	-0.404938	2.02731
C	-0.960989	0.24982	3.049789
C	-2.31157	0.442412	2.945478
C	-3.01769	-0.095085	1.845484
C	-4.423413	0.030213	1.726
C	-5.099367	-0.58086	0.699379
C	-4.405478	-1.371821	-0.229806
C	0.729611	-0.495445	-2.66097
H	3.407128	-4.429494	0.015447
H	4.918068	-2.7338	0.982147
H	5.389574	-0.357173	1.526067
H	4.706648	1.99499	1.515296
H	-0.891734	-4.44339	-1.928158
H	1.365206	-4.999236	-1.187369
H	-2.867301	-3.225978	-1.463178
H	3.09118	3.739178	0.85984
H	-2.659915	2.01963	-2.284343
H	-2.018271	-0.300314	-2.312003
H	-0.665397	6.108478	-0.829661
H	-2.148379	4.370924	-1.789886
H	0.819146	-0.508692	2.139444
H	-0.420632	0.62291	3.909401
H	-2.85639	0.981703	3.70968
H	-4.959275	0.605457	2.470143
H	-6.174182	-0.484535	0.623274
H	1.732652	-0.852405	-2.439292
H	0.187857	-1.284005	-3.183328
H	0.807503	0.365164	-3.32285
H	-4.953733	-1.91485	-0.989339
H	1.463526	5.478176	0.23138

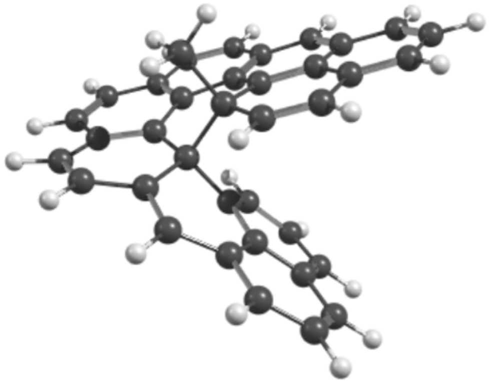

NC (broken-symmetry unrestricted singlet)

	X	Y	Z
C	1.270557	-3.045183	0.145142
C	1.236151	-1.810277	0.865917
C	0.054295	-3.591328	-0.294258
C	-1.148027	-2.878328	-0.214045
C	-1.153577	-1.543356	0.282601
C	0.013689	-1.104435	1.022904
C	-2.377643	-3.469542	-0.640837
C	-2.305665	-0.725422	0.016504
C	-3.538937	-2.774097	-0.567413
C	-3.538754	-1.385895	-0.240269
C	-2.305665	0.725422	-0.016504
C	-3.538754	1.385895	0.240269
C	-4.75251	0.662985	0.164296
C	-4.75251	-0.662985	-0.164296
C	-1.153577	1.543356	-0.282601
C	-3.538937	2.774097	0.567413
C	-2.377643	3.469542	0.640837
C	-1.148027	2.878328	0.214045
C	0.013689	1.104435	-1.022904
C	0.054295	3.591328	0.294258
C	1.270557	3.045183	-0.145142
C	1.236151	1.810277	-0.865917
C	2.436686	-1.3063	1.447205
C	2.372752	-0.153892	2.255779
C	1.155215	0.441179	2.520539
C	-0.009197	-0.026635	1.922406
C	-0.009197	0.026635	-1.922406
C	1.155215	-0.441179	-2.520539
C	2.372752	0.153892	-2.255779
C	2.436686	1.3063	-1.447205
C	3.65435	-1.988344	1.207725
C	3.680211	-3.157725	0.468711
C	2.5058	-3.697825	-0.041195
C	3.65435	1.988344	-1.207725
C	2.5058	3.697825	0.041195
C	3.680211	3.157725	-0.468711
H	0.046521	-4.583319	-0.728834
H	-2.367722	-4.499099	-0.974647
H	-4.483504	-3.247794	-0.802433
H	-5.683489	1.192391	0.320811
H	-5.683489	-1.192391	-0.320811
H	-4.483504	3.247794	0.802433

Thermochemistry
Temperature 298.150 Kelvin. Pressure: 1.00000 Atm.
Zero-point correction = 0.437297 (Hartree/Particle)
Thermal correction to Energy = 0.460814
Thermal correction to Enthalpy = 0.461758
Thermal correction to Gibbs Free Energy = 0.386256
Sum of electronic and zero-point Energies = -1383.986501
Sum of electronic and thermal Energies = -1383.962984
Sum of electronic and thermal Enthalpies = -1383.962040
Sum of electronic and thermal Free Energies = -1384.037542

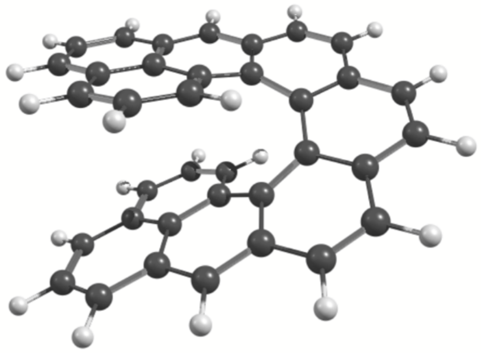

H	-2.367722	4.499099	0.974647
H	0.046521	4.583319	0.728834
H	3.283354	0.239167	2.688224
H	1.102451	1.292511	3.185416
H	-0.945318	0.458465	2.146613
H	-0.945318	-0.458465	-2.146613
H	1.102451	-1.292511	-3.185416
H	3.283354	-0.239167	-2.688224
H	4.567678	-1.585383	1.62552
H	4.620107	-3.667477	0.30265
H	2.527547	-4.63277	-0.586038
H	4.567678	1.585384	-1.62552
H	2.527547	4.63277	0.586038
H	4.620107	3.667478	-0.30265

NC transition state (broken-symmetry unrestricted singlet)

	X	Y	Z
C	0.591189	3.301236	-0.300003
C	0.786883	2.019776	-0.906754
C	-0.676636	3.62846	0.194867
C	-1.699395	2.682833	0.239148
C	-1.448558	1.363804	-0.228674
C	-0.271584	1.092835	-0.951898
C	-3.021782	3.001903	0.701252
C	-2.433595	0.324917	0.009934
C	-4.012237	2.078125	0.676223
C	-3.751511	0.709078	0.330262
C	-2.100897	-1.05363	-0.05295
C	-3.139658	-1.980506	-0.196379
C	-4.479559	-1.565857	0.00531
C	-4.770403	-0.271494	0.337897
C	-0.690201	-1.535286	-0.042989
C	-2.839172	-3.319045	-0.651498
C	-1.574587	-3.704149	-0.913951
C	-0.448845	-2.879723	-0.549561
C	0.273146	-1.059668	0.945865
C	0.824569	-3.386211	-0.559077
C	1.909724	-2.686402	0.0421
C	1.607071	-1.551494	0.855908
C	2.067903	1.674745	-1.443146
C	2.225806	0.389469	-2.066878
C	1.175223	-0.457718	-2.212827
C	-0.105881	-0.155889	-1.665477
C	-0.007225	-0.079191	1.896951

Thermochemistry
Temperature 298.150 Kelvin. Pressure: 1.00000 Atm.
Zero-point correction = 0.438474 (Hartree/Particle)
Thermal correction to Energy = 0.461124
Thermal correction to Enthalpy = 0.462068
Thermal correction to Gibbs Free Energy = 0.389123
Sum of electronic and zero-point Energies = -1383.690741
Sum of electronic and thermal Energies = -1383.938090
Sum of electronic and thermal Enthalpies = -1383.937146
Sum of electronic and thermal Free Energies = -1384.010091

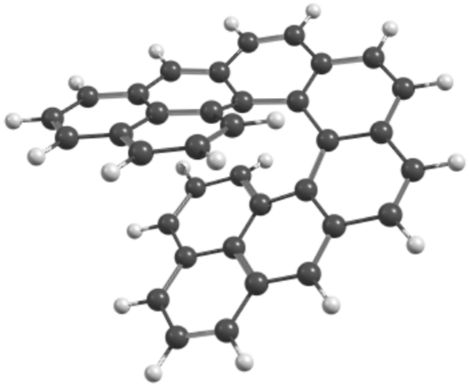

C	1.017934	0.5127	2.647667
C	2.325569	0.133047	2.478399
C	2.650093	-0.926417	1.597228
C	3.110019	2.580985	-1.330711
C	2.91467	3.8431	-0.739635
C	1.687594	4.204602	-0.247698
C	3.975037	-1.383312	1.42801
C	3.229565	-3.132019	-0.050498
C	4.255013	-2.464829	0.616416
H	-0.86168	4.630434	0.562228
H	-3.222401	4.013941	1.028839
H	-5.020203	2.34875	0.964186
H	-5.270452	-2.299167	-0.087413
H	-5.785455	0.023972	0.57011
H	-3.66754	-3.991336	-0.834936
H	-1.371668	-4.690178	-1.312341
H	1.004143	-4.373047	-0.967953
H	3.200782	0.124854	-2.455157
H	1.300465	-1.391561	-2.743528
H	-0.970022	-0.556049	-2.174704
H	-1.024535	0.228554	2.070824
H	0.766445	1.290005	3.356805
H	3.11836	0.616684	3.033928
H	4.087661	2.312345	-1.709872
H	3.745745	4.533059	-0.67749
H	1.54024	5.178246	0.20156
H	4.771762	-0.880663	1.961184
H	3.451266	-4.002186	-0.655023
H	5.275402	-2.80931	0.511753

c-NC without Ar

	X	Y	Z
C	3.357705	-3.186559	-0.101134
C	4.152443	-2.181574	0.397037
C	3.667533	-0.86455	0.460375
C	2.336504	-0.610459	0.049317
C	1.469738	-1.674441	-0.251937
C	2.01046	-2.948756	-0.442857
C	4.46284	0.228579	0.943092
C	3.980867	1.493802	0.981407
C	2.681462	1.813736	0.457838
C	1.881807	0.755456	-0.065373
C	-0.023914	-1.389071	-0.442039
C	-0.769259	-2.570761	-1.054981

Thermochemistry
Temperature 298.150 Kelvin. Pressure: 1.00000 Atm.
Zero-point correction = 0.441052 (Hartree/Particle)
Thermal correction to Energy = 0.463671
Thermal correction to Enthalpy = 0.464616
Thermal correction to Gibbs Free Energy = 0.391702
Sum of electronic and zero-point Energies = -1383.978079
Sum of electronic and thermal Energies = -1383.955450
Sum of electronic and thermal Enthalpies = -1383.954516
Sum of electronic and thermal Free Energies = -1384.027430

C	-0.046903	-3.739552	-1.48575
C	1.226759	-3.964343	-1.114187
C	-0.76103	-0.971184	0.844208
C	-2.182072	-0.959178	0.798347
C	-2.887138	-1.67356	-0.22064
C	-2.122889	-2.583452	-1.036782
C	2.200275	3.114746	0.398929
C	0.96305	3.40452	-0.18801
C	0.209427	2.343898	-0.788461
C	0.694353	1.028403	-0.737692
C	-1.04192	2.640538	-1.418015
C	-1.779144	1.547569	-2.025349
C	-1.334118	0.289221	-2.022366
C	-0.026767	-0.115813	-1.420987
C	0.438508	4.726675	-0.234092
C	-0.762932	4.98004	-0.83363
C	-1.506171	3.934865	-1.427379
C	-0.138464	-0.479438	1.969577
C	-0.873304	0.131256	3.00495
C	-2.23278	0.260045	2.914807
C	-2.925191	-0.298713	1.815952
C	-4.335967	-0.237697	1.708218
C	-4.990105	-0.868762	0.679148
C	-4.268414	-1.617254	-0.263899
H	3.760516	-4.182485	-0.235214
H	5.168917	-2.388436	0.706617
H	5.463532	0.014609	1.297027
H	4.587843	2.299179	1.375099
H	-0.604619	-4.505424	-2.010761
H	1.708661	-4.904284	-1.350702
H	-2.654643	-3.395202	-1.519847
H	2.797499	3.923864	0.801662
H	-2.721464	1.784915	-2.504107
H	-1.90344	-0.482684	-2.516724
H	-1.153549	5.988691	-0.859392
H	-2.456212	4.155375	-1.897341
H	0.933596	-0.531715	2.065391
H	-0.343725	0.523019	3.862969
H	-2.794431	0.766167	3.689456
H	-4.892797	0.303968	2.462095
H	-6.068795	-0.821428	0.611803
H	-4.797795	-2.176973	-1.024683
H	1.00989	5.528485	0.215688
H	0.609546	-0.44903	-2.254901

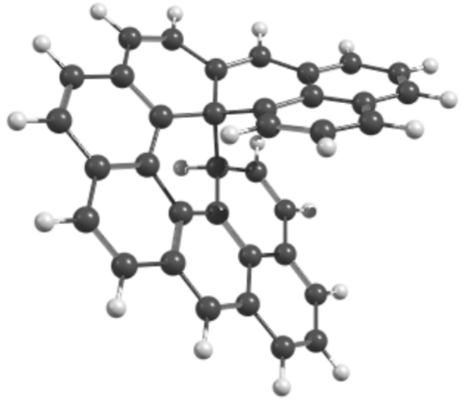

c-DMNC with Ar subsitutents (functional M06-2X/basis cc-pVTZ)

	X	Y	Z
C	0.490517	5.480843	0.14466
C	-0.8752	5.480716	0.143196
C	-1.58573	4.272906	0.071287
C	-0.8715	3.073832	-0.09232
C	0.528553	3.064133	-0.02117
C	1.209479	4.274143	-0.07473
C	-3.01499	4.218908	0.227945
C	-3.67872	3.045764	0.206106
C	-2.99806	1.809163	-0.08271
C	-1.59491	1.837695	-0.29019
C	1.249947	1.72104	0.053997
C	2.749515	1.863769	-0.16137
C	3.336483	3.156771	-0.44923
C	2.642067	4.292078	-0.3256
C	1.055457	0.941843	1.38122
C	1.848168	-0.25106	1.479123
C	3.07155	-0.38064	0.743918
C	3.556538	0.818158	0.089483
C	-3.68296	0.613368	-0.21292
C	-3.02117	-0.56571	-0.57189
C	-1.61656	-0.51316	-0.83261
C	-0.92123	0.702943	-0.72028
C	-0.91618	-1.71394	-1.17136
C	0.507622	-1.62955	-1.45048
C	1.178019	-0.48331	-1.38734
C	0.544962	0.846065	-1.10172
C	-3.70348	-1.82757	-0.67755
C	-2.98348	-2.95082	-0.9746
C	-1.59442	-2.89889	-1.21952
C	0.151557	1.203642	2.397737
C	-0.17281	0.157523	3.30588
C	0.416945	-1.0647	3.263495
C	1.496315	-1.27702	2.380032
C	2.252475	-2.47064	2.407946
C	3.375271	-2.60285	1.645778
C	3.82673	-1.54074	0.829372
C	0.650091	1.606067	-2.45082
C	5.10605	-1.71254	0.100562
C	5.221763	-1.40181	-1.25272
C	6.414915	-1.5948	-1.93932
C	7.508916	-2.09853	-1.24502

Thermochemistry
Temperature 298.150 Kelvin. Pressure: 1.00000 Atm.
Zero-point correction = 0.773765 (Hartree/Particle)
Thermal correction to Energy = 0.816350
Thermal correction to Enthalpy = 0.817294
Thermal correction to Gibbs Free Energy = 0.696930

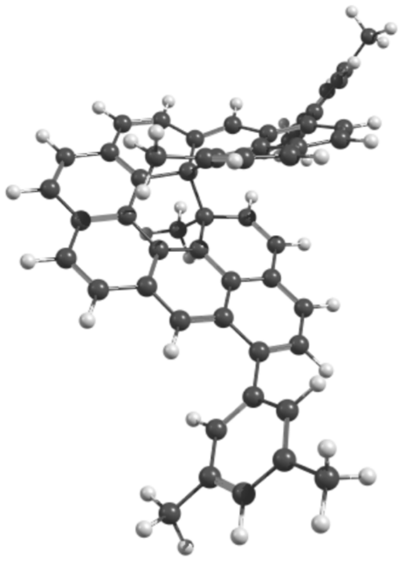

C	7.428143	-2.41763	0.106777
C	6.219374	-2.22762	0.764191
C	8.635927	-2.92403	0.84643
C	6.509194	-1.29823	-3.41097
C	-5.17006	-1.94038	-0.47923
C	-6.06	-1.21402	-1.27516
C	-7.43075	-1.35504	-1.12929
C	-7.91376	-2.2365	-0.1614
C	-7.05644	-2.97145	0.642438
C	-5.68146	-2.81464	0.470332
C	-8.38936	-0.59189	-2.00176
C	-7.58258	-3.91585	1.688184
H	1.038395	6.414444	-0.00742
H	-1.41806	6.408534	0.271269
H	-3.5442	5.148164	0.39779
H	-4.74951	3.013195	0.362478
H	4.39769	3.178207	-0.66471
H	3.122072	5.253753	-0.4537
H	4.622963	0.916975	-0.07031
H	-4.74865	0.597503	-0.03162
H	1.018211	-2.54344	-1.72946
H	2.22094	-0.45803	-1.66684
H	-3.50094	-3.89842	-1.05232
H	-1.06016	-3.80837	-1.46334
H	-0.93371	0.36179	4.04896
H	0.11926	-1.85461	3.940937
H	1.931227	-3.27305	3.060048
H	3.946724	-3.52123	1.667669
H	0.13589	2.563588	-2.42766
H	1.700006	1.7769	-2.69599
H	0.208215	0.994432	-3.23572
H	4.360772	-1.01829	-1.78606
H	8.446634	-2.2474	-1.76945
H	6.143657	-2.46203	1.819394
H	8.348028	-3.48693	1.732694
H	9.265065	-2.09328	1.170993
H	9.243931	-3.56763	0.211877
H	7.510558	-0.96644	-3.68182
H	6.287451	-2.1907	-3.99888
H	5.799514	-0.52588	-3.70342
H	-5.66661	-0.54328	-2.02977
H	-8.98539	-2.34936	-0.03778
H	-4.99554	-3.37435	1.095735
H	-9.11197	-0.0414	-1.39861

H	-7.86451	0.118095	-2.63806
H	-8.95222	-1.26996	-2.64479
H	-7.26514	-3.60716	2.684951
H	-7.20523	-4.92594	1.525451
H	-8.67023	-3.95315	1.675375
H	-0.51796	2.518991	2.732343
H	0.017865	3.385705	2.360884
H	-1.54623	2.563966	2.368375
H	-0.55943	2.596074	3.818921

DMNC with Ar subsitutents, unrestricted singlet (functional M06-2X/basis cc-pVTZ)

	X	Y	Z
C	-0.681292	-0.62988	0.04838
C	0.680998	5.629907	0.0485
C	1.39295	4.415784	0.201324
C	0.679205	3.197042	0.244159
C	-0.67938	3.197028	-0.24423
C	-1.39319	4.415731	-0.2013
C	2.814719	4.38871	0.310898
C	3.470606	3.208106	0.416752
C	2.750134	1.987834	0.62497
C	1.341677	2.035947	0.74259
C	-1.34182	2.035928	-0.74272
C	-2.75027	1.987749	-0.62507
C	-3.47079	3.207977	-0.41679
C	-2.81495	4.3886	-0.31087
C	-0.66806	0.877514	-1.30369
C	-1.32247	-0.38092	-1.19475
C	-2.7037	-0.45343	-0.8119
C	-3.40696	0.752743	-0.64411
C	3.406885	0.752865	0.64399
C	2.703687	-0.45335	0.811705
C	1.32243	-0.38093	1.194488
C	0.667963	0.87747	1.303488
C	0.618803	-1.57251	1.497598
C	-0.68485	-1.4808	2.015214
C	-1.21976	-0.2515	2.307996
C	-0.56088	0.942635	1.991547
C	3.306499	-1.72453	0.640923
C	2.543381	-2.8813	0.830877
C	1.241907	-2.81945	1.268794
C	0.560758	0.942783	-1.99178
C	1.219659	-0.2513	-2.30839
C	0.684811	-1.48064	-2.0157

Thermochemistry
Temperature 298.150 Kelvin. Pressure: 1.00000 Atm.
Zero-point correction = 0.769591 (Hartree/Particle)
Thermal correction to Energy = 0.812697
Thermal correction to Enthalpy = 0.813642
Thermal correction to Gibbs Free Energy = 0.693229

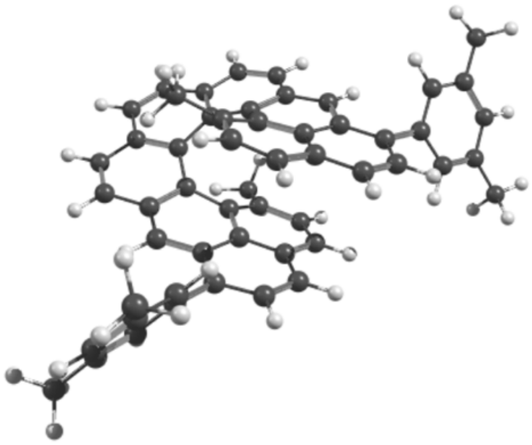

C	-0.61881	-1.57245	-1.498
C	-1.24185	-2.81944	-1.26925
C	-2.54328	-2.88137	-0.83121
C	-3.30643	-1.72465	-0.64112
C	-1.17784	2.217414	2.504296
C	-4.73139	-1.87856	-0.26379
C	-5.0723	-2.58957	0.880441
C	-6.40437	-2.78214	1.242171
C	-7.3956	-2.25675	0.42673
C	-7.08639	-1.54828	-0.7342
C	-5.75303	-1.36386	-1.0659
C	-8.18713	-1.01439	-1.60937
C	-6.74622	-3.52872	2.502341
C	4.731517	-1.87839	0.263813
C	5.753038	-1.36359	1.06608
C	7.086422	-1.54799	0.734652
C	7.395873	-2.25671	-0.42611
C	6.404838	-2.78222	-1.24164
C	5.072662	-2.58951	-0.88024
C	8.187055	-1.01319	1.609396
C	6.74678	-3.52968	-2.50126
H	-1.23414	6.560392	-0.06419
H	1.233795	6.560448	0.064399
H	3.35716	5.323106	0.242692
H	4.551977	3.166653	0.38977
H	-4.55216	3.166481	-0.38982
H	-3.35743	5.322968	-0.2426
H	-4.47012	0.73489	-0.45177
H	4.47004	0.735073	0.451631
H	-1.23413	-2.38957	2.22631
H	-2.1846	-0.18899	2.7972
H	3.018501	-3.84187	0.678011
H	0.681756	-3.72512	1.46293
H	2.18447	-0.1887	-2.79763
H	1.234099	-2.38938	-2.22692
H	-0.68167	-3.72507	-1.46348
H	-3.01834	-3.84197	-0.67834
H	-0.45998	3.03285	2.551474
H	-2.02075	2.546881	1.894053
H	-1.55816	2.037714	3.510143
H	-4.28332	-2.99041	1.507225
H	-8.43638	-2.40356	0.69431
H	-5.49355	-0.83106	-1.97292
H	-7.78657	-0.4293	-2.43494

H	-8.86547	-0.37973	-1.03827
H	-8.77932	-1.82916	-2.0281
H	-7.80918	-3.75795	2.54922
H	-6.19089	-4.46448	2.565447
H	-6.49042	-2.93801	3.38335
H	5.493367	-0.83074	1.973016
H	8.436715	-2.4036	-0.69341
H	4.283811	-2.99043	-1.50715
H	8.859677	-0.37102	1.039868
H	7.785951	-0.43539	2.439818
H	8.785483	-1.82699	2.021014
H	6.197242	-4.46917	-2.55991
H	7.810983	-3.7523	-2.55151
H	6.483825	-2.94292	-3.38278
H	1.177649	2.217648	-2.50439
H	0.459763	3.033067	-2.55141
H	2.020567	2.547071	-1.89413
H	1.557943	2.038101	-3.51027

NC with Ar substituents, unrestricted singlet (functional M06-2X/basis cc-pVTZ)

	X	Y	Z
C	-0.681139	5.710463	-0.000489
C	0.681359	5.710428	0.000582
C	1.400519	4.494224	0.101941
C	0.6979	3.273765	0.189083
C	-0.69781	3.273811	-0.18906
C	-1.40037	4.494301	-0.10189
C	2.829017	4.481975	0.111467
C	3.507148	3.313932	0.1807
C	2.81722	2.086702	0.443724
C	1.418241	2.112406	0.626798
C	-1.4182	2.112514	-0.62688
C	-2.81716	2.086862	-0.44377
C	-3.50704	3.314103	-0.18067
C	-2.82886	4.482117	-0.11141
C	-0.79967	0.945993	-1.2181
C	-1.48576	-0.29201	-1.16753
C	-2.85563	-0.34661	-0.7493
C	-3.50879	0.868043	-0.48707
C	3.508802	0.867866	0.487013
C	2.855554	-0.34677	0.74915
C	1.485671	-0.29215	1.16732
C	0.799655	0.945889	1.217968
C	0.804238	-1.48263	1.533516
C	-0.49576	-1.40192	2.062701

Thermochemistry
Temperature 298.150 Kelvin. Pressure: 1.00000 Atm.
Zero-point correction = 0.713478 (Hartree/Particle)
Thermal correction to Energy = 0.753957
Thermal correction to Enthalpy = 0.754901
Thermal correction to Gibbs Free Energy = 0.637870

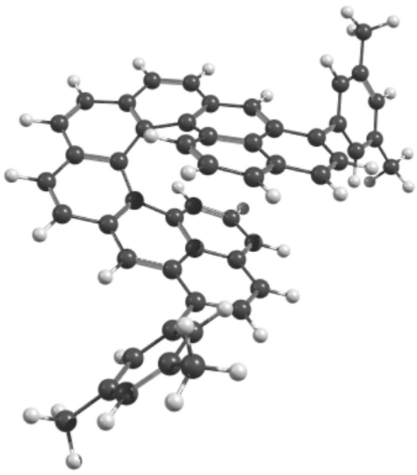

C	-1.08676	-0.17225	2.266135
C	-0.44987	0.989418	1.84969
C	3.489466	-1.61186	0.630522
C	2.754592	-2.77253	0.89013
C	1.457184	-2.72302	1.344907
C	0.449889	0.989482	-1.84978
C	1.086673	-0.17218	-2.26637
C	0.49559	-1.40183	-2.06306
C	-0.80438	-1.4825	-1.53383
C	-1.45734	-2.72289	-1.34517
C	-2.75468	-2.77238	-0.89024
C	-3.48956	-1.6117	-0.6306
C	-4.90984	-1.75003	-0.23436
C	-5.25274	-2.53712	0.858578
C	-6.58332	-2.71465	1.232865
C	-7.57182	-2.09622	0.481952
C	-7.26126	-1.30956	-0.62713
C	-5.92912	-1.14178	-0.97181
C	-8.35995	-0.67492	-1.43498
C	-6.92563	-3.54555	2.438964
C	4.909807	-1.75014	0.234387
C	5.928988	-1.14193	0.972043
C	7.261143	-1.30961	0.62748
C	7.57186	-2.09626	-0.4816
C	6.58349	-2.71465	-1.23266
C	5.252821	-2.53707	-0.85859
C	8.359751	-0.67426	1.434883
C	6.925868	-3.54613	-2.43834
H	-1.23556	6.639845	0.02236
H	1.235829	6.639778	-0.02225
H	3.351869	5.423679	0.003057
H	4.585569	3.288593	0.091136
H	-4.58546	3.288805	-0.09107
H	-3.35167	5.423842	-0.003
H	-4.56333	0.872076	-0.25094
H	4.563372	0.871846	0.251021
H	-1.01071	-2.31553	2.331149
H	-2.06435	-0.10837	2.724799
H	3.248448	-3.72888	0.774694
H	0.924703	-3.63439	1.584498
H	2.06426	-0.10831	-2.72505
H	1.010428	-2.31546	-2.33167
H	-0.9249	-3.63426	-1.58483
H	-3.24852	-3.72873	-0.77473

H	-4.46632	-3.00978	1.436429
H	-8.61165	-2.23036	0.759682
H	-5.66991	-0.54908	-1.8409
H	-7.95581	-0.04246	-2.22302
H	-9.00464	-0.06339	-0.80292
H	-8.98799	-1.43566	-1.90044
H	-7.99447	-3.74424	2.490887
H	-6.40023	-4.50026	2.417939
H	-6.63383	-3.03302	3.356962
H	5.669635	-0.54933	1.841153
H	8.611731	-2.2304	-0.75916
H	4.466472	-3.00969	-1.43657
H	8.998579	-0.05536	0.803973
H	7.955507	-0.04871	2.228358
H	8.993778	-1.43446	1.892984
H	6.406417	-4.50402	-2.41289
H	7.99566	-3.73849	-2.49422
H	6.627044	-3.03759	-3.35627
H	-0.93412	1.943253	1.999881
H	0.934236	1.943277	-1.99988

c-NC with Ar substituents (functional M06-2X/basis cc-pVTZ)

	X	Y	Z
C	0.477760	5.546614	-0.014243
C	-0.87965	5.547879	0.19097
C	-1.59486	4.342211	0.159362
C	-0.88945	3.141302	-0.04293
C	0.513543	3.130315	-0.04187
C	1.191751	4.34116	-0.1373
C	-3.02058	4.286799	0.355846
C	-3.69046	3.117527	0.321879
C	-3.02283	1.884248	-0.01071
C	-1.62565	1.914021	-0.24868
C	1.24059	1.788449	-0.00581
C	2.725873	1.926293	-0.29644
C	3.284477	3.212198	-0.66113
C	2.601432	4.350552	-0.49461
C	1.095128	1.034138	1.322943
C	1.954397	-0.08128	1.531068
C	3.137238	-0.25295	0.742958
C	3.554233	0.897885	-0.03675
C	-3.71157	0.691771	-0.15597
C	-3.06072	-0.47826	-0.565

Thermochemistry
Temperature 298.150 Kelvin. Pressure: 1.00000 Atm.
Zero-point correction = 0.717900 (Hartree/Particle)
Thermal correction to Energy = 0.756772
Thermal correction to Enthalpy = 0.757716
Thermal correction to Gibbs Free Energy = 0.645301

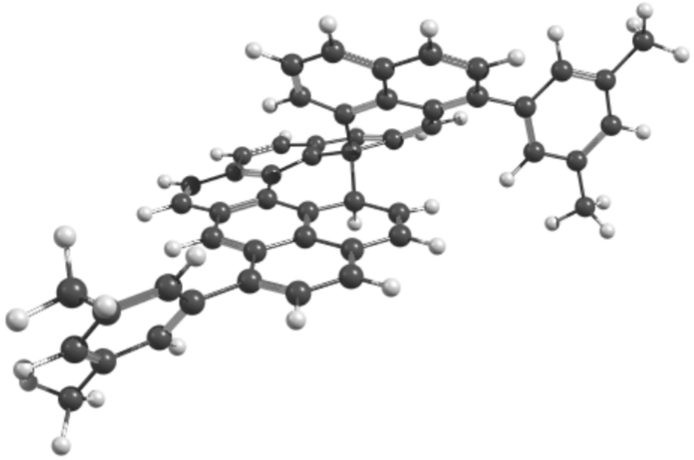

C	-1.66383	-0.41924	-0.86099
C	-0.96661	0.791175	-0.72652
C	-0.97175	-1.6049	-1.2639
C	0.444499	-1.50904	-1.58436
C	1.118555	-0.36448	-1.51087
C	0.489981	0.931317	-1.11474
C	-3.74464	-1.73709	-0.69288
C	-3.03287	-2.84871	-1.049
C	-1.65158	-2.7876	-1.33459
C	0.11011	1.285359	2.244952
C	-0.12978	0.405862	3.320075
C	0.610392	-0.73107	3.461783
C	1.681539	-0.98999	2.578246
C	2.506573	-2.13094	2.724594
C	3.583062	-2.32201	1.910412
C	3.936033	-1.37147	0.922118
C	5.165578	-1.6004	0.128839
C	5.169751	-1.44789	-1.25662
C	6.314867	-1.68687	-2.00613
C	7.474621	-2.07605	-1.34413
C	7.505012	-2.23637	0.037039
C	6.341511	-2.00293	0.760124
C	8.780504	-2.61878	0.736486
C	6.292469	-1.56288	-3.505
C	-5.20466	-1.85901	-0.45552
C	-6.11822	-1.10902	-1.20112
C	-7.48392	-1.25698	-1.01903
C	-7.93712	-2.16992	-0.06603
C	-7.05575	-2.92869	0.688179
C	-5.68665	-2.76422	0.480264
C	-8.46888	-0.46646	-1.83603
C	-7.5499	-3.90515	1.719963
H	1.021882	6.480619	-0.07643
H	-1.41376	6.477387	0.341483
H	-3.54224	5.214132	0.556729
H	-4.7576	3.086912	0.501592
H	4.326316	3.231968	-0.9561
H	3.072527	5.309405	-0.66885
H	4.608341	0.995306	-0.2664
H	-4.77246	0.67088	0.051345
H	0.944001	-2.41505	-1.90613
H	2.157648	-0.33087	-1.80285
H	-3.55239	-3.79356	-1.14386
H	-1.1266	-3.68781	-1.62807

H	-0.92696	0.630917	4.015105
H	0.40793	-1.43353	4.260139
H	2.266541	-2.85113	3.496504
H	4.195292	-3.20774	2.017216
H	4.255686	-1.15725	-1.75954
H	8.375718	-2.26073	-1.91891
H	6.352912	-2.11261	1.838122
H	8.576633	-3.13428	1.673586
H	9.372922	-1.73202	0.968929
H	9.391965	-3.26837	0.111756
H	7.246192	-1.19707	-3.88319
H	6.104773	-2.53273	-3.96915
H	5.508392	-0.88214	-3.8325
H	-5.7481	-0.41424	-1.94568
H	-9.00453	-2.28888	0.085306
H	-4.98189	-3.34281	1.066289
H	-9.1574	0.082085	-1.19245
H	-7.96251	0.248585	-2.48153
H	-9.06801	-1.12555	-2.46577
H	-7.22784	-3.6095	2.719287
H	-7.15312	-4.90341	1.53292
H	-8.63661	-3.965	1.718595
H	-0.51782	2.157898	2.149558
H	0.526805	1.581478	-2.00226

11. References

1. Fulmer, G. R.; Miller, A. J.; Sherden, N. H.; Gottlieb, H. E.; Nudelman, A.; Stoltz, B. M.; Bercaw, J. E.; Goldberg, K. I. NMR Chemical Shifts of Trace Impurities: Common Laboratory Solvents, Organics, and Gases in Deuterated Solvents Relevant to the Organometallic Chemist. *Organometallics* **2010**, *29*, 2176–2179, DOI: https://doi.org/10.1021/om100106e
2. Kogiso, T.; Yamamoto, K.; Suemune, H.; Usui, K. Synthesis and characterization of 1,8-naphthalimide with [6]helicene skeleton. *Org. Biomol. Chem.* **2012**, *10*, 2934–2936, DOI: https://doi.org/10.1039/C2OB25223F
3. Clark, R. C.; Reid, J. S. The analytical calculation of absorption in multifaceted crystals. *Acta Crystallogr. Sect. A* **1995**, *51*, 887–897, DOI: https://doi.org/10.1107/S0108767395007367
4. CrysAlisPro. Rigaku Oxford Diffraction Ltd: Yarnton, England **2019**.
5. Dolomanov, O. V.; Bourhis, L. J.; Gildea, R. J.; Howard, J. A. K.; Puschmann, H. OLEX2: a complete structure solution, refinement and analysis program. *J. Appl. Crystallogr.* **2009**, *42*, 339–341, DOI: https://doi.org/10.1107/S0021889808042726
6. Sheldrick, G. M. SHELXT – Integrated Space-Group and Crystal-Structure Determination. *Acta Crystallogr. Sect. A* **2015**, *71*, 3–8, DOI: https://doi.org/10.1107/S2053273314026370
7. Sheldrick, G. M. Crystal structure refinement with SHELXL. *Acta Cryst.* **2015**, C*71*, 3–8, DOI: https://doi.org/10.1107/S2053229614024218
8. Spek, A. L. Structure validation in chemical crystallography. *Acta Crystallogr. Sect. D* **2009**, *D65*, 148–155, DOI: https://doi.org/10.1107/S090744490804362X
9. Gaussian 16, Rev. C.01. M. J. Frisch, G. W. Trucks, H. B. Schlegel, G. E. Scuseria, M. A. Robb, J. R. Cheeseman, G. Scalmani, V. Barone, G. A. Petersson, H. Nakatsuji, X. Li, M. Caricato, A. v. Marenich, J. Bloino, B. G. Janesko, R. Gomperts, B. Mennucci, H. P. Hratchian, J. v. Ortiz, A. F. Izmaylov, J. L. Sonnenberg, D. Williams-Young, F. Ding, F. Lipparini, F. Egidi, J. Goings, B. Peng, A. Petrone, T. Henderson, D. Ranasinghe, V. G. Zakrzewski, J. Gao, N. Rega, G. Zheng, W. Liang, M. Hada, M. Ehara, K. Toyota, R. Fukuda, J. Hasegawa, M. Ishida, T. Nakajima, Y. Honda, O. Kitao, H. Nakai, T. Vreven, K. Throssell, J. A. Jr. Montgomery, J. E. Peralta, F. Ogliaro, M. J. Bearpark, J. J. Heyd, E. N. Brothers, K. N. Kudin, V. N. Staroverov, T. A. Keith, R. Kobayashi, J. Normand, K. Raghavachari, A. P. Rendell, J. C. Burant, S. S. Iyengar, J. Tomasi, M. Cossi, J. M. Millam, M. Klene, C. Adamo, R. Cammi, J. W. Ochterski, R. L. Martin, K. Morokuma, O. Farkas, J. B. Foresman, D. J. Fox, Wallingford, CT, 2016.
10. WinSim EPR simulator, https://www.niehs.nih.gov/research/resources/software/toxpharm/tools/index.cfm, retrieved 14.07.2025.
